# Effect of Sacubitril/Valsartan vs Valsartan on Left Atrial Volume in Patients With Pre–Heart Failure With Preserved Ejection Fraction: The PARABLE Randomized Clinical Trial

**DOI:** 10.1001/jamacardio.2023.0065

**Published:** 2023-03-08

**Authors:** Mark Ledwidge, Jonathan D. Dodd, Fiona Ryan, Claire Sweeney, Katherine McDonald, Rebecca Fox, Elizabeth Shorten, Shuaiwei Zhou, Chris Watson, Joseph Gallagher, Niall McVeigh, David J. Murphy, Kenneth McDonald

**Affiliations:** 1 St Vincent’s Screening to Prevent Heart Failure (STOP-HF) Unit, St Vincent’s University Healthcare Group, Dublin, Ireland; 2 School of Medicine, University College Dublin, Dublin, Ireland; 3 Department of Radiology, St Vincent’s University Hospital, Dublin, Ireland; 4 School of Medicine, Dentistry and Biomedical Sciences, Wellcome-Wolfson Institute for Experimental Medicine, Queens University, Belfast, Northern Ireland

## Abstract

**Importance:**

Pre–heart failure with preserved ejection fraction (pre-HFpEF) is common and has no specific therapy aside from cardiovascular risk factor management.

**Objective:**

To investigate the hypothesis that sacubitril/valsartan vs valsartan would reduce left atrial volume index using volumetric cardiac magnetic resonance imaging in patients with pre-HFpEF.

**Design, Setting, and Participants:**

The Personalized Prospective Comparison of ARNI [angiotensin receptor/neprilysin inhibitor] With ARB [angiotensin-receptor blocker] in Patients With Natriuretic Peptide Elevation (PARABLE) trial was a prospective, double-blind, double-dummy, randomized clinical trial carried out over 18 months between April 2015 and June 2021. The study was conducted at a single outpatient cardiology center in Dublin, Ireland. Of 1460 patients in the STOP-HF program or outpatient cardiology clinics, 461 met initial criteria and were approached for inclusion. Of these, 323 were screened and 250 asymptomatic patients 40 years and older with hypertension or diabetes, elevated B-type natriuretic peptide (BNP) greater than 20 pg/mL or N-terminal pro-b-type natriuretic peptide greater than 100 pg/mL, left atrial volume index greater than 28 mL/m^2^, and preserved ejection fraction greater than 50% were included.

**Interventions:**

Patients were randomized to angiotensin receptor neprilysin inhibitor sacubitril/valsartan titrated to 200 mg twice daily or matching angiotensin receptor blocker valsartan titrated to 160 mg twice daily.

**Main Outcomes and Measures:**

Maximal left atrial volume index and left ventricular end diastolic volume index, ambulatory pulse pressure, N-terminal pro-BNP, and adverse cardiovascular events.

**Results:**

Among the 250 participants in this study, the median (IQR) age was 72.0 (68.0-77.0) years; 154 participants (61.6%) were men and 96 (38.4%) were women. Most (n = 245 [98.0%]) had hypertension and 60 (24.0%) had type 2 diabetes. Maximal left atrial volume index was increased in patients assigned to receive sacubitril/valsartan (6.9 mL/m^2^; 95% CI, 0.0 to 13.7) vs valsartan (0.7 mL/m^2^; 95% CI, −6.3 to 7.7; *P* < .001) despite reduced markers of filling pressure in both groups. Changes in pulse pressure and N-terminal pro-BNP were lower in the sacubitril/valsartan group (−4.2 mm Hg; 95% CI, −7.2 to −1.21 and −17.7%; 95% CI, −36.9 to 7.4, respectively; *P* < .001) than the valsartan group (−1.2 mm Hg; 95% CI, −4.1 to 1.7 and 9.4%; 95% CI, −15.6 to 4.9, respectively; *P* < .001). Major adverse cardiovascular events occurred in 6 patients (4.9%) assigned to sacubitril/valsartan and 17 (13.3%) assigned to receive valsartan (adjusted hazard ratio, 0.38; 95% CI, 0.17 to 0.89; adjusted *P* = .04).

**Conclusions and Relevance:**

In this trial of patients with pre-HFpEF, sacubitril/valsartan treatment was associated with a greater increase in left atrial volume index and improved markers of cardiovascular risk compared to valsartan. More work is needed to understand the observed increased cardiac volumes and long-term effects of sacubitril/valsartan in patients with pre-HFpEF.

**Trial Registration:**

ClinicalTrials.gov Identifier: NCT04687111

## Key Points

**Question:**

Can neprilysin inhibition improve markers of cardiovascular structure and function in patients with pre–heart failure with preserved ejection fraction?

**Findings:**

In this randomized clinical trial of 250 asymptomatic patients, sacubitril/valsartan vs valsartan was associated with a reduction in blood pressure, pulse pressure, and N-terminal pro-b-type natriuretic peptide; an increase in maximal left atrial volume index measured by cardiac magnetic resonance imaging despite lower filling pressures; less decline in kidney function; and fewer serious adverse cardiovascular events.

**Meaning:**

These findings could reflect improved cardiac and vascular compliance or adverse cardiac remodeling, and more work is required to understand the long-term implications.

## Teaser

This randomized clinical trial evaluates the efficacy of sacubitril/valsartan vs valsartan in the treatment of left ventricular diastolic function in patients with pre–heart failure with preserved ejection fraction.

## Introduction

The 2022 American Heart Association/American College of Cardiology (AHA/ACC) guidelines for management of heart failure highlight the importance of community identification of pre–heart failure (pre-HF; also termed stage B HF) because of its association with increased heart failure and cardiovascular risk.^1^ This approach, supported by natriuretic peptide screening, was shown in the St Vincent’s Screening to Prevent Heart Failure (STOP-HF) randomized clinical trial^2^ to reduce major adverse cardiovascular events and progression of pre-HF, most of which was associated with pre-HF with preserved ejection fraction (pre-HFpEF). However, the STOP-HF trial^2^ lacked a specific pharmacological therapy, and the intervention was directed at a general population with cardiovascular risk factors. Studies of other pharmacological therapies in patients with cardiovascular and metabolic risk factors in whom pre-HF was present have shown reduced incident heart failure and cardiovascular events.^3,4,5,6,7,8,9^ In patients with pre-HF with reduced left ventricular ejection fraction, angiotensin-converting enzyme inhibitor therapy has been associated with a reduction in incident heart failure.^10^ However, the high prevalence of pre-HFpEF in community populations, an estimated 30% to 63% of older adults with hypertension or diabetes,^11,12,13^ underlines a need for more studies targeting this cohort.

The pre-HFpEF diagnosis requires the absence of symptoms of heart failure, which is highly subjective. Nonetheless, the 2022 AHA/ACC guidelines say it can be diagnosed in asymptomatic patients with preserved ejection fraction by the presence of at least 1 of the following: structural heart disease, including elevated left atrial size; increased filling pressures; cardiovascular risk factors with increased natriuretic peptides; or persistently elevated cardiac troponins, in the absence of competing diagnoses.^1^ Aging, long-standing hypertension, diabetes, obesity, and chronic inflammatory comorbidities predispose individuals to abnormalities in cardiac chamber and vascular compliance leading to tissue stiffness, increased filling pressures, hypertrophy, reduced stroke volume, and reactive myocardial fibrosis.^14,15,16,17,18,19,20,21,22^ The release of natriuretic peptides, predominantly in the myocardium, is an endogenous protective response, helping to preserve compliance and function by enhancing cardiac and vascular smooth muscle cyclic guanosine monophosphate.^23,24,25^ Sacubitril/valsartan is a first-in-class angiotensin neprilysin receptor inhibitor that reduces degradation of a range of vasoactive substances including natriuretic peptide. Along with other therapies,^26,27,28^ it has been investigated in symptomatic HFpEF.^29,30^ Yet, to our knowledge, no study to date has evaluated sacubitril/valsartan in a prespecified cohort with pre-HFpEF.

## Methods

### Hypothesis, Trial Design, and Oversight

The Personalized Prospective Comparison of ARNI [angiotensin receptor/neprilysin inhibitor] with ARB [angiotensin-receptor blocker] in Patients With Natriuretic Peptide Elevation (PARABLE) randomized, double-blind, double-dummy, active comparator trial was conducted from April 2015 to June 2021 at a single outpatient cardiology center in Dublin, Ireland. The trial was designed to investigate the hypothesis that sacubitril/valsartan vs valsartan would reduce left atrial volume index over 18 months using volumetric cardiac magnetic resonance imaging in patients with pre-HFpEF. Ethical approval was obtained by the St Vincent’s University Hospital Ethics Committee and competent authority approval was obtained from the Health Protection Regulatory Authority in Ireland (EudraCT: 2015-002928-53; ClinicalTrials.gov identifier: NCT04687111). The protocol is available in Supplement 1. Further details on trial oversight, supply of investigational medicinal products, and contractual arrangements with the funder are provided in eMethods 1 in Supplement 2.

### Trial Patients

Patients gave written informed consent to participate in the study before any study-related assessments were performed. Patients 40 years and older with systemic hypertension (medicated for more than 1 month) and/or type 2 diabetes were included and had the following at screening or within 6 months prior to screening: elevated blood B-type natriuretic peptide (BNP; 20 pg/mL to 280 pg/mL) or N-terminal pro b-type natriuretic peptide (NT-proBNP; 100 pg/ml to 1000 pg/mL); and enlarged transthoracic left atrial volume index (LAVI; >28 mL/m^2^ obtained using echocardiography).^22^ As detailed in the protocol, we reduced the entry BNP and LAVI thresholds early in the study from 35 pg/mL and 34 mL/m^2^ respectively to include a low-risk population, based on the observation that almost 3 in 10 major adverse cardiovascular events in the original STOP-HF trial^2^ occurred in patients with baseline BNP in the range of 20 to 49 pg/mL. Detailed inclusion and exclusion criteria are provided in eTable 1 in Supplement 2. Patients were ineligible if they had a history of or any features of symptomatic heart failure, left ventricular systolic dysfunction (ejection fraction <50%), or serious valvular disease or kidney dysfunction.

### Trial Procedures and Interventions

The study design is presented in eFigure 1 in Supplement 2. The trial consisted of a screening period, a washout period of 36 hours (if participants were previously taking an angiotensin-converting enzyme inhibitor), and a randomized, double-blind, double-dummy, treatment period including dose titration in 2 arms. The intervention arm received sacubitril/valsartan starting at 49 mg/51 mg twice daily, titrated after 2 weeks to 97 mg/103 mg twice daily in addition to usual medical care and a valsartan dummy. The active control arm received valsartan, 80 mg twice daily, titrated after 2 weeks to 160 mg twice daily in addition to usual medical care and a sacubitril/valsartan dummy.

Lower starting doses were used for patients with low systolic blood pressure (SBP; ≥100 mm and <110 mm Hg) or on low or no dose of angiotensin-converting enzyme inhibitor or an angiotensin receptor blocker at the baseline visit. Full details of the medication manufacture, randomization, blinding, allocation concealment, and titration protocol are in eMethods 2 and eTable 2 in Supplement 2.

### Study Assessments

Full details of the study assessments and schedule are included in eMethods 3 and eTable 3 in Supplement 2. Routine assessment involved clinical examination, biochemistry, and evaluation of adverse events and was supplemented with natriuretic peptide levels, electrocardiography monitoring, 24-hour ambulatory blood pressure monitoring and Doppler echocardiography at baseline, 9 months, and the final study visit (18 months). Cardiac magnetic resonance imaging (MRI) was performed at 2 centers (St. Vincent’s University Healthcare Group and Blackrock Clinic) at baseline and at 18 months using 1.5 Tesla scanners (Aera; Siemens Healthcare; and Signa HD; GE Healthcare, respectively). The imaging protocol included a balanced steady-state free precession cine stack of the entire left atrium and left ventricle using a 6- or 8-mm slice thickness and 2-mm slice gap. All scans were reported by 2 independent fellowship-trained cardiothoracic attending radiologists blinded to the randomization arm and all clinical details (intracorrelation and intercorrelation coefficients of 0.95 and 0.96, respectively). Further details regarding the cardiac MRI protocol and analysis are presented in eMethods 3 and eTable 3 in Supplement 2.

### Trial Outcomes

The primary end point was the adjusted change in maximal LAVI measured by volumetric cardiac MRI, indexed to body surface area using the DuBois formula. Secondary outcomes were changes in NTproBNP, pulse pressure (PP), cardiac MRI measurements of structure (left ventricular end diastolic volume index, minimal LAVI, and left ventricular mass index) and function (left atrial and left ventricular stroke volume index, left atrial emptying fraction, and left ventricular ejection fraction), indirect assessment of left ventricular filling pressure using Doppler echocardiography, average ratio of early transmitral flow velocity to early diastolic mitral annular tissue velocity (E/e′), and time to first major adverse cardiovascular event over the course of the study. Major adverse cardiovascular event was defined as cardiovascular death or a serious adverse cardiovascular event due to arrythmia (including atrial fibrillation/flutter), transient ischemic attack, stroke, valvular heart disease, myocardial infarction, deep vein thrombosis or pulmonary embolus, or heart failure requiring hospital admission. Serious adverse events related to the cardiac or vascular system were defined using European Medicines Agency guidelines as events that cause death, are life threatening, require inpatient hospitalization or prolongation of existing hospitalization, or result in serious disability/incapacity.^31^

### Sample Size and Statistical Methods

Details on sample size calculations and statistical methods are presented in eMethods 4 in Supplement 2. Based on previous work in patients with HFpEF using Doppler echocardiography^29^ and the use of a more precise imaging technique (volumetric cardiac MRI) for the primary end point, the expected effect size (2.0/5.0 = 0.40) required 96 patients in each group, using a 2-tailed α value of 5% and β of 20%. The study also had at least 80% power with a 2-tailed α of 5% to detect a 6 g/m^2^–difference in left ventricular mass index change by cardiac MRI and a 2-unit difference in E/e′ or e′ using tissue Doppler measurements. Primary and secondary outcome measures were analyzed with adjustment for the following variables, unless otherwise specified: age, sex, diabetes, hypertension, obesity, and vascular disease and for baseline measures of the outcome of interest. Categorical end points (eg, major adverse cardiovascular event) were analyzed through hazard ratios by deploying Cox proportional hazards modeling. COVID-19–related procedures are outlined in eMethods 3 in Supplement 2. Before the completion of the trial, data lock, and unblinding of the final data set, we prespecified a per-protocol analysis of the primary end point and analysis of the major adverse cardiovascular event end point prior to the first COVID-19 pandemic lockdown in Ireland (March 14, 2020).

## Results

**Figure 1.**
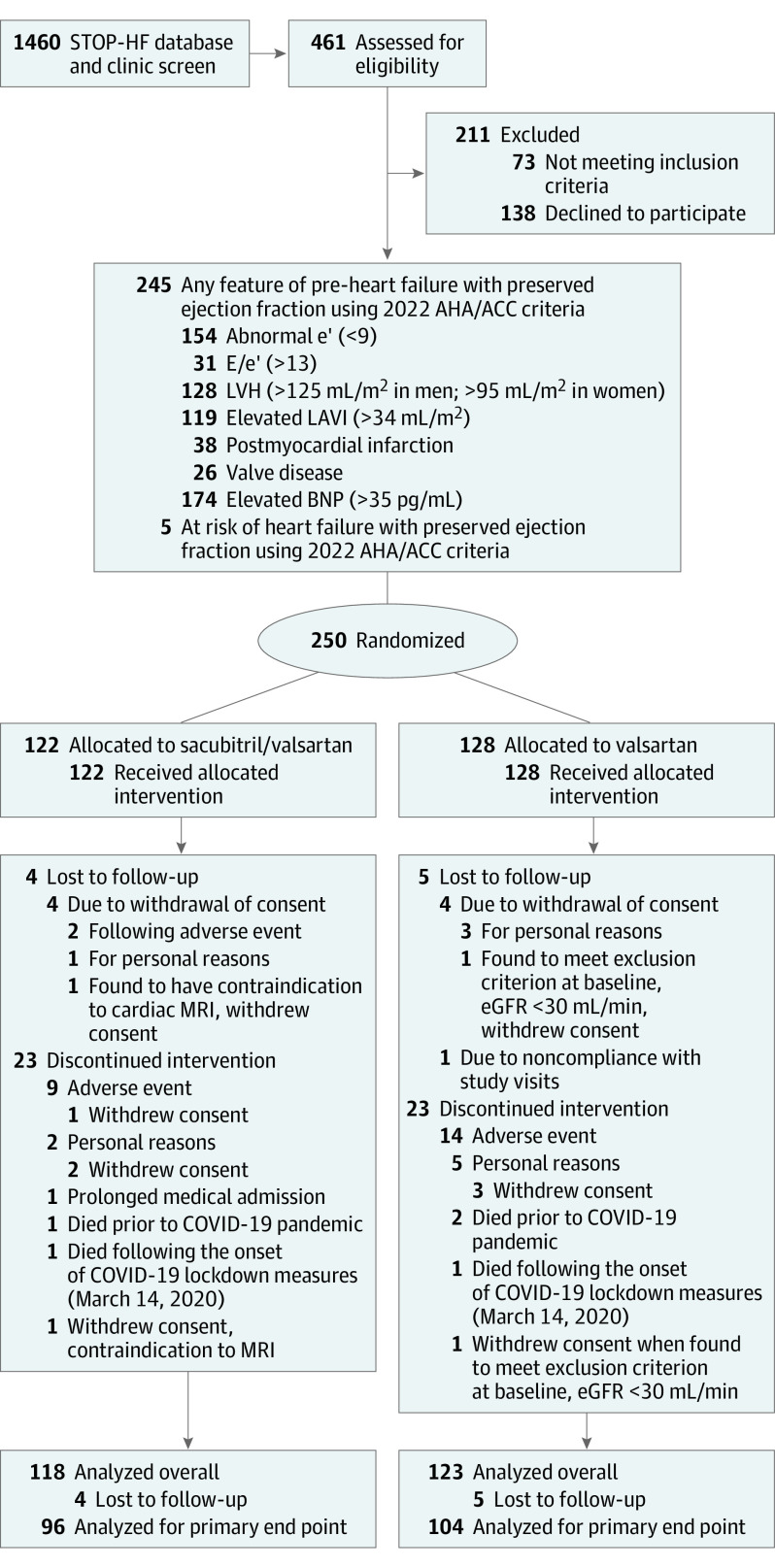
CONSORT Diagram AHA/ACC indicates American Heart Association/American College of Cardiology; BNP, B-type natriuretic peptide; e′, mitral annular early diastolic velocity; E/e′, the ratio between early mitral inflow velocity and e′; eGFR, estimated glomerular filtration rate; LAVI, left atrial volume index; LVH, left ventricular hypertrophy; MRI, magnetic resonance imaging; STOP-HF, St Vincent’s Screening to Prevent Heart Failure.

From April 5, 2015, to December 12, 2018, among 1460 patients with available BNP and Doppler echocardiography attending the St Vincent’s University Healthcare Group STOP-HF program^2^ or outpatient cardiology clinics, 461 (31.6%) had BNP greater than 20 pg/mL, ejection fraction greater than 50%, and LAVI greater than 28 mL/m^2^. Of these, 323 were willing to participate in screening for eligibility and inclusion in the PARABLE trial. After exclusions, 250 were randomly assigned to double-blind, double-dummy treatment with sacubitril/valsartan (n = 122) or valsartan (n = 128). In total, 245 of these patients (98%) had at least 1 feature of pre-HFpEF according to the 2022 AHA/ACC guidelines. The CONSORT diagram, including a breakdown of these data, is presented in Figure 1.

Baseline characteristics by treatment group are given in Table 1. The median (IQR) population age was 72.0 (68.0-77.0) years; 154 participants (61.6%) were men and 96 (38.4%) were women. A total of 207 patients (82.8%; 102 in sacubitril/valsartan and 105 in valsartan) were overweight, defined as body mass index greater than 25 kg/m^2^. Population blood pressure control, dyslipidemia management, glucose control, and kidney function was generally good. Almost all participants (n = 245 [98.0%]) had hypertension and 60 (24.0%) had type 2 diabetes. Median (IQR) blood levels of BNP and echocardiographic LAVI at baseline were 57 (33-100) pg/mL and 33.2 (30.7-38.0) mL/m^2^, respectively. Tricuspid regurgitation velocity was measurable in less than half of the population. Similar numbers of patients with sacubitril/valsartan (n = 33) and valsartan (n = 28) had abnormal (ie, >35 mm Hg) pulmonary artery systolic pressure and 34 patients total (13.6%) had high-risk H2FPEF scores (≥6) (Table 1). More detailed information on titration and cardiometabolic medications at baseline and follow-up are shown in eTables 4 and 5 in Supplement 2, respectively. Forty-six patients (17.6% of the study population) discontinued study medication prematurely (Figure 1). The trial terminated when the last patient completed the follow-up period (June 11, 2021). Paired MRI studies were available for analysis of the primary end point in 200 patients. Of the 241 patients monitored to completion, only 1 patient in the valsartan arm had a confirmed diagnosis of COVID-19.

**Table 1.** Characteristics of the Patient Population at Baseline

Characteristic	No. (%)	
	Sacubitril/valsartan (n = 122)	Valsartan (n = 128)
Age, median (IQR), y	72.0 (68.0-78.0)	72.0 (67.8-76.0)
Female	43 (35.2)	53 (41.4)
Male	79 (64.8)	75 (58.6)
Body weight, kg	83.2 (75.2-93.8)	82.0 (71.1-91.5)
BMI, median (IQR)	29.0 (26.3-31.4)	28.8 (26.0-32.1)
Heart rate, median (IQR), bpm^a^	61.0 (56.0-70.0)	64.0 (57.0-70.0)
Blood pressure, mm Hg		
Systolic	135 (125-147)	137 (123-149)
Diastolic	74.0 (68.0-82.0)	75.0 (68.0-83.0)
Total cholesterol, median (IQR), mg/dL^b^	160.5 (135.3-194.5)	170.1 (147.0-197.2)
LDL cholesterol, median (IQR), mg/dL^c^	85.1 (61.9-100.5)	85.1 (68.8-112.1)
Triglycerides, median (IQR), mg/dL^d^	124.0 (79.7-168.3)	119.6 (79.7-168.3)
HbA1c, median (IQR), %^e^	5.7 (5.4-6.1)	5.6 (5.4-6.1)
Hb, median (IQR), g/dL^f^	13.4 (12.5-14.4)	13.5 (12.5-14.4)
eGFR, median (IQR), mL/min^g^	78.0 (64.0-90.0)	77.0 (65.8-90.0)
BNP, median (IQR), pg/mL^h^	59.0 (33.5-109)	52.4 (32.7-91.2)
NTproBNP, median (IQR), pg/mL^i^	136 (88.2-278)	138 (84.0-247)
Hypertension	120 (98.4)	125 (97.7)
Dyslipidemia	109 (89.3)	113 (88.3)
Diabetes	35 (28.7)	25 (19.5)
Angina	10 (8.20)	11 (8.59)
Myocardial infarction	18 (14.8)	20 (15.6)
Coronary artery disease	49 (40.2)	57 (44.5)
Paroxysmal atrial fibrillation	15 (12.3)	10 (7.81)
Stroke/transient ischemic attack	8 (6.56)	15 (11.7)
Peripheral vascular disease	2 (1.64)	4 (3.12)
Chronic kidney disease	23 (18.9)	24 (18.8)
H2FPEF score, median (IQR)^j^	3.50 (3.00-5.00)	3.00 (2.00-5.00)
High-risk H2FPEF score (≥6)^j^	21 (17.2)	13 (10.2)
Pretrial ACE inhibitor	60 (49.2)	63 (49.2)
Pretrial ARB	52 (42.6)	49 (38.3)
α-Blocker	21 (17.2)	25 (19.5)
β-Blocker	70 (57.4)	69 (53.9)
Calcium channel blocker	49 (40.2)	61 (47.7)
Statin	98 (80.3)	102 (79.7)
Thiazide diuretic	33 (27.0)	41 (32.0)
Aspirin	74 (60.7)	86 (67.2)
Nonaspirin antiplatelet	8 (6.56)	11 (8.59)
DOAC	11 (9.02)	8 (6.25)
Warfarin	3 (2.46)	0 (0.00)
Oral antidiabetic	28 (23.0)	23 (18.0)
Insulin	6 (4.92)	5 (3.91)

Abbreviations: ACE, angiotensin-converting enzyme; ARB, angiotensin receptor blocker; BMI, body mass index, calculated as weight in kilograms divided by height in meters squared; BNP, B-type natriuretic peptide; bpm, beats per minute; DOAC, direct-acting oral anticoagulant; eGFR estimated glomerular filtration rate; H2FPEF, heart failure with preserved ejection fraction; Hb, hemoglobin; HbA1c, glycated hemoglobin; LDL, low-density lipoprotein; NTproBNP, N-terminal pro B-type natriuretic peptide.

^a^ Missing 1 in the sacubitril/valsartan group because of data recording error.

^b^ Missing 10 in the sacubitril/valsartan group because of phlebotomy difficulties (3) and laboratory errors (7) and 4 in the valsartan group because of laboratory errors. Phlebotomy difficulties included venous access and volume obtained.

^c^ Missing 13 in the sacubitril/valsartan group because of phlebotomy difficulties (3), laboratory errors (7), and high triglycerides (3) and 8 in the valsartan group because of laboratory errors (4) and high triglycerides (4).

^d^ Missing 10 in the sacubitril/valsartan group because of phlebotomy difficulties (3) and laboratory errors (7) and 4 in the valsartan group because of laboratory errors.

^e^ Missing 3 in the sacubitril/valsartan group because of phlebotomy difficulties (2) and a laboratory error (1) and 7 in the valsartan group because of laboratory errors.

^f^ Missing 2 in the sacubitril/valsartan group because of phlebotomy difficulties and 1 in the valsartan group because of laboratory error.

^g^ Missing 2 in the sacubitril/valsartan group because of phlebotomy difficulties.

^h^ Missing 1 in the valsartan group because of laboratory error (NTproBNP obtained).

^i^ Missing 4 in the sacubitril/valsartan group and 3 in the valsartan group because of laboratory errors (3 in each group) and 1 phlebotomy difficulty in the sacubitril/valsartan group. BNP was obtained in all 7 patients.

^j^ The H2FPEF score comprises 1 point for each of the following: older than 60 years, treatment with 2 or more antihypertensive drugs, E/e′ greater than 9, and pulmonary artery systolic pressure greater than 35 mm Hg; 2 points for obesity (BMI >30 kg/m^2^); 3 points for atrial fibrillation. A score of 0-1 is considered low risk, 2-5 intermediate risk, and 6 or greater high risk.^32^

### Effect of Treatment on Cardiac Structure

**Figure 2.**
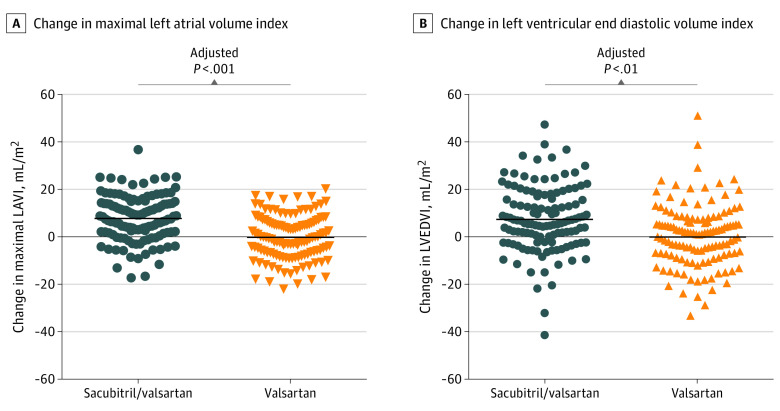
Change in Maximal Left Atrial Volume Index (LAVI) and Left Ventricular End Diastolic Volume Index (LVEDVI) Cardiac magnetic resonance imaging–estimated change in maximal LAVI was greater in patients assigned to receive sacubitril/valsartan (6.9 mL/m^2^; 95% CI, 0.0 to 13.7) vs valsartan (0.7 mL/m^2^; 95% CI, −6.3 to 7.7) (*P* < .001). Similarly, sacubitril/valsartan treatment showed greater increase in LVEDVI (7.1 mL/m^2^; 95% CI, −1.7 to 15.9) vs the valsartan group (1.4 mL/m^2^; 95% CI, −7.2 to 10.0) (*P* = .02). *P* value indicates between-group comparison of change values over time, adjusted for age, sex, hypertension, diabetes, obesity, vascular disease, and baseline measures of the outcome measure. Bars indicate means.

Detailed Doppler echocardiography and cardiac MRI imaging data are presented in Table 2. Cardiac MRI-estimated change in maximal LAVI was greater in patients assigned to receive sacubitril/valsartan (6.9 mL/m^2^; 95% CI, 0.0 to 13.7) vs valsartan (0.7 mL/m^2^; 95% CI, −6.3 to 7.7; *P* < .001) (Figure 2A). Similarly, sacubitril/valsartan treatment was associated with greater increase in left ventricular end diastolic volume index (7.1 mL/m^2^; 95% CI, −1.7 to 15.9) vs the valsartan group (1.4 mL/m^2^; 95% CI, −7.2 to 10.0; *P* = .02) (Figure 2B).

**Table 2.** Cardiac Magnetic Resonance Imaging, Doppler Echocardiography, 24-Hour Blood Pressure, and Natriuretic Peptide Outcome Measures^a^

Variable	Sacubitril/valsartan				Valsartan				*P* value, between-group change
	Baseline		Change		Baseline		Change		
	Median (IQR)	No.	Median (IQR)	No.	Median (IQR)	No.	Median (IQR)	No.	
Body surface area, m^2^	1.97 (1.81 to 2.08)	122	−0.00 (−0.03 to 0.02)	115	1.91 (1.76 to 2.04)	128	0.00 (−0.02 to 0.02)	121	.47
Body weight, kg	83.2 (75.2 to 93.8)	122	−0.5 (−2.5 to 1.5)	115	82.0 (71.1 to 91.5)	127	0.0 (−2.3 to 2.1)	121	.31
Heart rate, bpm	60.0 (54.0 to 70.0)	117	−3.0 (−10.0 to 2.0)	99	61.0 (53.0 to 68.5)	119	0.0 (−6.8 to 7.0)	106	.006
LA volume (maximum), mL	89.9 (77.30 to 109.2)	114	13.6 (3.8 to 30.0)^b^	96	95.4 (80.5 to 114.1)	118	−0.2 (−12.6 to 10.5)	104	<.001
LA volume index (maximum), mL/m^2^	46.7 (40.5 to 52.2)	114	6.8 (1.8 to 14.5)^b^	96	51.5 (44.2 to 60.0)	118	−0.7 (−6.8 to 5.3)	104	<.001
LA volume (minimum), mL	46.5 (39.1 to 61.8)	114	7.6 (0.1 to 17.3)^b^	96	52.3 (38.9 to 64.5)	118	0.4 (−8.4 to 9.3)	104	<.001
LA volume index (minimum), mL/m^2^	25.0 (20.5 to 30.6)	114	4.0 (−0.3 to 9.5)^b^	96	26.6 (20.9 to 33.7)	118	0.2 (−4.2 to 4.4)	104	<.001
LA stroke volume, mL	41.8 (34.7 to 48.3)	114	7.1 (−1.5 to 13.8)^b^	96	44.7 (37.3 to 52.8)	118	−1.5 (−8.5 to 4.8)^c^	104	<.001
LA stroke volume index, mL/m^2^	21.3 (17.7 to 25.0)	114	3.6 (−1.0 to 7.6)^b^	96	24.1 (20.4 to 27.8)	118	−0.7 (−4.0 to 2.9)	104	<.001
LA emptying fraction, %	47.5 (37.7 to 52.3)	113	0.6 (−4.1 to 5.0)	95	47.0 (40.7 to 53.3)	118	−0.5 (−4.5 to 3.8)	104	.31
LA diastolic stiffness index, mm Hg/mL/m^2^	0.40 (0.34 to 0.47)	110	−0.06 (−0.12 to −0.03)^b^	89	0.35 (0.30 to 0.43)	113	0.00 (−0.04 to 0.06)	97	<.001
LV end diastolic volume, mL	134.5 (109.8 to 163.1)	116	12.1 (−2.2 to 29.7)^b^	97	142.6 (114.8 to 170.6)	119	1.6 (−14.1 to 11.1)	104	<.001
LV end diastolic volume index, mL/m^2^	68.3 (60.5 to 79.0)	116	6.8 (−2.2 to 13.7)^b^	97	74.2 (63.1 to 88.1)	119	1.4 (−7.0 to 5.9)	104	<.001
LV end systolic volume, mL	51.4 (38.8 to 61.5)	116	3.9 (−5.3 to 11.8)^d^	97	51.9 (39.2 to 65.6)	119	0.0 (−6.3 to 6.6)	104	.02
LV end systolic volume index, mL/m^2^	25.5 (20.8 to 31.1)	116	2.1 (−2.5 to 6.1)^d^	97	27.1 (21.7 to 33.2)	119	0.3 (−3.3 to 3.7)	104	.02
LV stroke volume index, mL/m^2^	43.8 (37.6 to 50.6)	116	3.1 (−0.87 to 9.5)^b^	97	47.9 (39.8 to 54.1)	119	−0.6 (−5.8 to 5.1)	104	<.001
LV ejection fraction, %	61.8 (57.1 to 67.2)	116	0.2 (−3.0 to 4.6)	97	62.0 (58.9 to 66.9)	119	0.4 (−3.3 to 3.0)	104	.56
LV mass, g	136.5 (110.7 to 162.0)	116	−2.6 (−10.2 to 4.9)^c^	97	134.3 (106.0 to 172.3)	119	−2.7 (−13.5 to 6.1)	104	.96
LV mass index, g/m^2^	70.1 (59.5 to 80.7)	116	−1.1 (−4.8 to 2.2)	97	69.3 (59.3 to 83.8)	119	−0.9 (−7.1 to 4.0)	104	.88
LV end diastolic pressure, mm Hg	18.6 (17.4 to 19.7)	118	−0.8 (−1.8 to 0.5)^b^	107	17.8 (16.8 to 19.3)	122	−0.4 (−1.3 to 0.5)^c^	112	.17
LV diastolic stiffness index, mm Hg/mL/m^2^	0.27 (0.23 to 0.32)	112	−0.04 (−0.06 to 0.00)^b^	89	0.25 (0.21 to 0.29)	114	0.00 (−0.03 to 0.02)	97	<.001
LA volume index (echocardiography), mL/m^2^	33.50 (30.60 to 37.40)	122	0.59 (−2.91 to 4.26)	115	33.20 (30.80 to 38.50)	128	1.08 (−2.07 to 5.65)	120	.26
E, ms	70.6 (61.7 to 82.3)	121	1.0 (−14.1 to 13.8)	111	67.1 (56.2 to 79.4)	128	2.0 (−6.2 to 9.9)	118	.39
e′, ms	6.6 (5.9 to 7.5)	118	0.5 (−0.5 to 1.9)^d^	109	7.0 (5.9 to 8.4)	121	0.5 (−0.5 to 1.4)^c^	112	.38
E/e′, mean	11.1 (9.1 to 13.0)	118	−1.3 (−3.0 to 0.9)^b^	107	9.9 (8.1 to 12.3)	122	−0.6 (−2.2 to 0.8)^c^	112	.17
24-h SBP, mm Hg	128.0 (122.0 to 134.0)	120	−5.0 (−12.5 to 1.0)^b^	111	130.0 (121.3 to 138.0)	126	−2.0 (−9.3 to 7.0)	116	.03
24-h DBP, mm Hg	68.0 (63.8 to 73.0)	120	−2.0 (−5.0 to 1.0)^d^	107	69.0 (65.0 to 75.0)	126	−1.0 (−5.8 to 4.0)	114	.14
24-h PP, mm Hg	60.0 (54.0 to 65.0)	120	−3.0 (−7.0 to 0.5)^b^	111	60.5 (52.0 to 66.0)	126	−1.0 (−6.0 to 4.0)	116	.01
24-h heart rate, bpm	60.0 (56.0 to 67.0)	120	1.0 (−2.0 to 4.0)	111	61.0 (56.0 to 69.0)	126	1.0 (−2.3 to 5.0)^c^	116	.62
Arterial elastance, mm Hg/mL/m^2^	1.6 (1.3 to 2.1)	116	−0.2 (−0.5 to 0.1)^b^	97	1.5 (1.3 to 2.0)	119	0.0 (−0.3 to 0.3)	104	<.001
SBP/stroke volume index, mm Hg/mL/m^2^	2.60 (2.30 to 3.20)	114	−0.30 (−0.80 to −0.01)^b^	92	2.50 (2.10 to 2.90)	119	0.00 (−0.40 to 0.30)	102	<.001
PP/stroke volume index, mm Hg/mL/m^2^	1.4 (1.2 to 1.7)	114	−0.2 (−0.5 to 0.0)^b^	92	1.3 (1.1 to 1.6)	119	0.0 (−0.2 to 0.2)	102	<.001
Total arterial compliance, mL/mm Hg	1.4 (1.1 to 1.8)	116	0.1 (−0.1 to 0.5)^c^	97	1.5 (1.1 to 1.9)	119	0.0 (−0.4 to 0.2)	104	.006
Systemic vascular resistance, mm Hg/mL/min	1541 (1206 to 1941)	116	−102 (−335 to 113)^c^	97	1394 (1147 to 1722)	119	−10 (−211 to 249)	104	<.001
BNP, pg/mL	59.1 (33.5 to 109.0)	122	8.0 (−20.2 to 49.4)^c^	116	52.4 (32.7 to 91.3)	127	6.2 (−13.0 to 34.5)^d^	120	.89
N-terminal BNP, pg/mL	136.5 (88.3 to 278.5)	118	−22.0 (−70.0 to 30.5)	111	138.0 (84.0 to 247.0)	125	25.5 (−15.0 to 104.5)^b^	116	<.001

Abbreviations: BNP, B-type natriuretic peptide; bpm, beats per minute; DBP, diastolic blood pressure; E/e′, the ratio between early mitral inflow velocity (E) and mitral annular early diastolic velocity (e′); LA, left atrium; LV, left ventricle; PP, pulse pressure; SBP, systolic blood pressure.

^a^ Among patients randomized to sacubitril/valsartan, 6 did not attend for baseline cardiac magnetic resonance imaging (MRI; 1 was found to have recent surgery clips contraindicating MRI, 1 had a physical disability, and 4 refused for personal reasons) and 23 did not attend follow-up (4 were lost to follow-up, 2 died, 1 had a prolonged medical admission, and 16 refused for personal reasons, including concerns about attending due to COVID-19). Among those randomized to valsartan, 9 did not attend for baseline cardiac MRI (all refused for personal reasons) and 24 did not attend follow-up (5 were lost to follow-up, 3 died, 1 had a non-MRI compatible pacemaker inserted, and 15 for personal reasons). Differences in LV and LA absolute numbers relate to incomplete left atrial acquisitions.

^b^ Within-group change, *P* < .001.

^c^ Within-group change, *P* < .05.

^d^ Within-group change, *P* < .01.

In post hoc analyses, we found that sacubitril/valsartan was associated with a reduction in left atrial and left ventricular diastolic stiffness index compared to valsartan. PP, arterial stiffness, and systemic vascular resistance were also reduced, while arterial compliance was increased in the sacubitril/valsartan vs valsartan treatment groups (Table 2). Change in maximal LAVI also remained significant when adjusted for changes in PP or SBP. However, there was a significant interaction between the changes in maximal LAVI and PP (odds ratio, 0.98; 95% CI, 0.97-0.99; *P* = .002) as well as SBP (odds ratio, 0.97; 95% CI, 0.96-0.99; *P* = .002). The LVEDV standardized to a filling pressure of 30 mm Hg (EDV30) was increased by a median (IQR) 13.8 mL (0.36 to 30.0) in the sacubitril/valsartan group vs 3.85 (−12.76 to 12.3); 30.0 in those treated with valsartan (*P* < .001). Left ventricular mass index was reduced in both groups without between-group differences. An analysis of the primary end point including only those patients who completed the study cardiac MRI per-protocol in the same institution prior to onset of COVID-19 restrictions showed the same result as the primary analysis (eFigure 2 in Supplement 2). LAVI measured by echocardiography did not demonstrate any significant within- or between-group changes at 18 months (Table 2).

### Effect of Treatment on Cardiac Function

Sacubitril/valsartan was associated with increased change in left atrial stroke volume index and left ventricular stroke volume index compared with valsartan. There were no between-group differences in the change in left ventricular ejection fraction or left atrial emptying fraction. Doppler echocardiography–measured E/e′ was significantly reduced in both treatment groups with no between-group difference (Table 2).

### Effect of Treatment on 24-Hour Ambulatory Blood Pressure, Pulse Pressure, and Estimated Glomerular Filtration Rate

Ambulatory blood pressure data over 24 hours showed significant reductions in SBP, diastolic blood pressure, and PP in patients treated with sacubitril/valsartan vs those treated with valsartan (Table 2). Using repeated measures analysis, the adjusted estimated marginal mean change in PP over the study was −4.2 mm Hg (95% CI, −7.2 to −1.21) in patients assigned to sacubitril/valsartan vs −1.2 mm Hg (95% CI, −4.1 to 1.7) in those assigned to valsartan (*P* < .001). More detail on biochemistry measures at baseline and follow-up are presented in eTables 6 and 7 in Supplement 2. There were no changes in body weight (Table 1) or hemoglobin (eTable 7 in Supplement 2) in either group. The reduction in mean (SD) estimated glomerular filtration rate in patients assigned to valsartan (−4.9 [11.4] mL/min/1.72 m^2^) was significantly greater than in those assigned to sacubitril/valsartan (−0.9 [10.6] mL/min/1.72 m^2^; *P* = .005).

### Effect of Treatment on Natriuretic Peptide

There was a significant reduction in NTproBNP over time observed in the sacubitril/valsartan vs valsartan group, which was sustained throughout the study (Table 2). Using repeated measures analysis, N-terminal pro-BNP decreased by −17.7% (95% CI, −36.9 to 7.4) in patients treated with sacubitril/valsartan vs an increase of 9.4% (95% CI, −15.6 to 4.9) in patients treated with valsartan (*P* < .001) over the study period. BNP levels were maintained at baseline levels throughout the study.

In post hoc analyses, small but significant differences between the 2 groups at baseline were noted in the mean (SD) body surface area among those who attended for cardiac MRI (sacubitril/valsartan 1.96 [0.22] m^2^ vs valsartan 1.90 [0.21] m^2^; *P* = .04), median (IQR) Doppler echocardiographic E/e′ (sacubitril/valsartan 11.1 [9.10-to 13.0] vs valsartan 9.87 [8.05 to 12.3], *P* = .01) and mean (SD) cardiac MRI measured LAVI max (sacubitril/valsartan 47.4 [9.59] vs valsartan 52.4 [11.3]; *P* = .01).

### Adverse Cardiovascular Events

**Figure 3.**
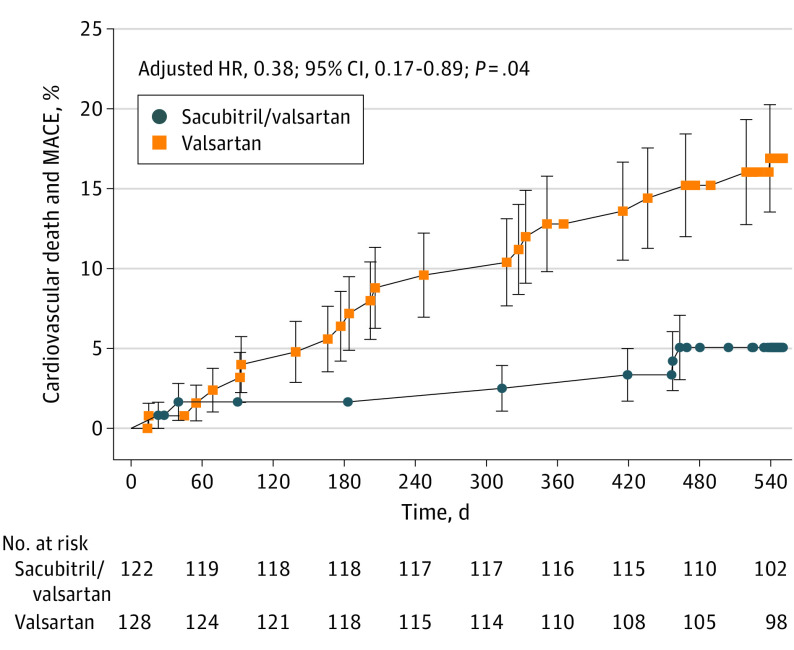
Time to Cardiovascular Death and First Major Adverse Cardiovascular Event (MACE) Sacubitril/valsartan vs valsartan was associated with an unadjusted hazard ratio (HR) for cardiovascular death and major adverse cardiovascular events of 0.35 (95% CI, 0.17 to 0.74; *P* = .006). Error bars indicate 95% CIs.

Overall, both treatments appeared to be well tolerated (all serious adverse event data are presented in eTable 8 in Supplement 2). There were 55 serious adverse events reported in patients assigned to sacubitril/valsartan vs 69 in those assigned to valsartan treatment (time-to-event analysis in eFigure 3 in Supplement 2). Four people developed symptomatic HFpEF (2 in each treatment group). The most common category of serious adverse cardiovascular event was atrial fibrillation or flutter, which occurred in 5 people treated with sacubitril/valsartan and 10 people treated with valsartan, 4 and 6 of whom, respectively, did not have any history of atrial fibrillation or flutter at baseline. There were 4 deaths that occurred during the 18-month study period, 3 in the valsartan group (2 cardiovascular deaths and 1 cancer death) and 1 (cancer death) in the sacubitril/valsartan group. Major adverse cardiovascular events (including cardiovascular deaths) occurred in 6 patients (4.9%) in the sacubitril/valsartan group and in 17 (13.3%) in the valsartan group. The time to first major adverse cardiovascular event is presented in Figure 3 and showed reduced risk in those treated with sacubitril/valsartan (adjusted hazard ratio, 0.38; 95% CI, 0.17 to 0.89; adjusted *P* = .04).

## Discussion

Pre-HFpEF is common, has no specific therapy aside from cardiovascular risk factor management, and is growing due to aging populations, increased prevalence of diabetes, persistent hypertension, and obesity. The 2022 AHA/ACC guidelines for heart failure^1^ and the 2021 Universal Definition and Classification of Heart Failure^33^ recommend increased focus on presymptomatic HF, citing the STOP-HF trial,^2^ despite concerns raised about large numbers of patients with the condition.^34^ However, the STOP-HF trial lacked a specific therapy. The PARABLE trial builds on this and other work to date in pre-HFpEF^35^ by targeting a population in which neprilysin inhibition and downstream preservation of natriuretic peptide may be of benefit, despite some evidence that circulating neprilysin levels are not elevated in patients who are presymptomatic.^36^

The effect of neprilysin inhibition on the primary end point in PARABLE was unexpected. Increases in maximal LAVI and left ventricular end diastolic volume index are ordinarily associated with a poorer outlook. While treatment with sacubitril/valsartan vs valsartan in a post–myocardial infarction population did not alter cardiac volumes,^37^ sacubitril/valsartan in symptomatic HFpEF was associated with a small decrease in LAVI measured by echocardiography over 9 months.^29^ However, cardiac chamber volume increases with sacubitril/valsartan in PARABLE were seen using more precise volumetric cardiac MRI. This occurred in the setting of reduced NT-proBNP, SBP, and PP as well as preservation of kidney function relative to valsartan. Could the evidence of reduced time to major adverse cardiovascular event, albeit in small numbers, suggest this LA enlargement is beneficial?

The aging myocardium of sedentary adults is associated with cardiac and vascular stiffness, reduced cardiac chamber size and stroke volumes.^20,38^ An increase in LVEDV due to afterload reduction, reduced cardiac stiffness and improved vascular compliance in older adults with stiff hearts and normal ejection fraction may be a marker of healthier or more successful aging.^39^ Exercise training augments cyclic guanosine monophosphate signaling^40^ and can reverse myocardial stiffness and increase LVEDV in patients with pre-HFpEF.^41,42^ LAVI is known to increase in endurance athletes.^43^ In PARABLE, the observed reduction in filling pressures, vascular stiffness, and cardiac chamber stiffness could explain a change in the pressure-volume relationship, with increased EDV30 in those treated with sacubitril/valsartan vs valsartan therapy. Interactions were seen between cardiac volume changes and beneficial PP changes. Similarly, natriuretic peptide augmentation of cyclic guanosine monophosphate and reversal of cardiomyocyte stiffness, mediated by titin hypophosphorylation, has been shown to increase cardiac chamber volumes in animal models.^18^

Conversely, increased cardiac chamber volumes in PARABLE could reflect adverse effects of neprilysin inhibition, which is known to preserve proremodeling vasoconstrictor peptides.^44^ Atrial natriuretic peptide, augmented by sacubitril/valsartan, can promote natriuretic peptide receptor-A–mediated adipogenesis, potentially increasing epicardial adipose tissue,^45^ in turn associated with worsening left atrial reservoir function in patients with HFpEF but not in those with HFrEF.^45,46^ More work is needed to understand the unexpected results in PARABLE and to balance them with the consistent improvement with sacubitril/valsartan vs valsartan in important surrogate markers of cardiovascular risk, such as SBP, PP, estimated glomerular filtration rate, and NTproBNP.

### Limitations

There are a number of important limitations to the present study. PARABLE is a phase II, single-center randomized clinical trial in a selected population of predominantly European ancestry. The follow-up time was limited to 18 months and overlapped with the COVID-19 pandemic. Nine patients were lost to follow-up, 46 withdrew from the treatment phase, not all of whom were willing to return for the follow-up cardiac MRI, and a further 49 had delayed cardiac MRI examination. There were differences in the baseline characteristics of the population, including in the primary end point. While the primary analysis adjusted for these baseline differences and there were no baseline differences in left ventricular end diastolic volume index, we cannot exclude a contribution of regression to the mean in the imaging results. Confirmation of asymptomatic HFpEF at baseline was carried out by an experienced cardiology fellow and heart failure nurse specialist. However, 34 patients (13.6%) had high H2FpEF scores, and we did not carry out formal functional assessments, such as the Kansas City Cardiology Questionnaire or 6-minute walk test, nor did we carry out cardiac MRI assessments of patients at an interim 9-month period. Accordingly, we cannot comment on the time course of the observations reported. PARABLE is not designed or powered to provide definitive evidence on the cardiovascular outcomes over longer follow-up. Moreover, the generalizability of the PARABLE study maybe limited by the exclusion criteria adopted and the homogenous ancestry of the participating population.

## Conclusions

The results of the PARABLE trial showed that sacubitril/valsartan can increase left atrial volume index in the setting of reduced markers of filling pressure in hypertension or diabetes in patients with pre-HFpEF. This may reflect improved vascular compliance and reduced cardiac chamber stiffness mediated pharmacologically by natriuretic peptide modulating therapy. However, more work is required to exclude adverse effects of increased chamber volumes and to understand the long-term benefits and risks of sacubitril/valsartan in treating pre-HFpEF.

## Notes

Supplement 1. Trial protocol

Supplement 2. eMethods 1. Trial design, Oversight, Investigational Medicinal Product and Randomisation eTable 1. PARABLE Inclusion and Exclusion Criteria eFigure 1. Design of the PARABLE trial eMethods 2. Medication Titration Protocol eTable 2. Study Titration Schedule eMethods 3. Description of Study Assessments eTable 3. Overview of Study Assessments eMethods 4. Detailed Description of Sample Size Calculations and Statistical Methods eTable 4. Detailed Cardiometabolic Medications at Baseline eTable 5. Detailed Cardiometabolic Medications at Follow up eTable 6. Additional Biochemistry at Baseline eTable 7. Additional Biochemistry at Follow Up eFigure 2. Change in per-protocol maximal left atrial volume index using cardiac MRI eFigure 3. Time to first serious adverse cardiovascular event eTable 8. Serious Adverse Event (SAE) over the study data by System-Organ-Class

Supplement 3. Data sharing statement
