# Supplementary material for: Effect of Sacubitril/Valsartan vs Valsartan on Left Atrial Volume in Patients With Pre–Heart Failure With Preserved Ejection Fraction: The PARABLE Randomized Clinical Trial
Source: JAMA Cardiol. 2023 Mar 8;8(4):366–75. doi: 10.1001/jamacardio.2023.0065 (PMC9996460; doi:10.1001/jamacardio.2023.0065)
Supplement: Supplement 1. — Trial protocol [file jamacardiol-e230065-s001.pdf]

CLINICAL TRIAL PROTOCOL

1. STUDY TITLE

Personalised prospective comparison of ARni with ArB in patients with natriuretic peptide eLEvation

The PARABLE study.

HBT-PTCL-01. Version 10.0

Effective date: 18/02/2021

2. STUDY SPONSOR

The Heartbeat Trust
3 Crofton Terrace,
Dun Laoghaire,
Co Dublin

3. APPLCIATION DETAILS

Principal Investigators:

Kenneth McDonald MD, FRCPI
Consultant Cardiologist
Heart Failure Unit
St. Vincent's University Hospital
Dublin 4

Mark Ledwidge PhD, MPSI
Research Director
Heart Failure Unit
St. Vincent's University Hospital
Dublin 4

Co-Investigators:

Joe Gallagher, MB BCh BAO BA
Heart Failure Unit
St. Vincent's University Hospital
Dublin 4

Rory O'Hanlon, MD, FRCPI
Consultant Cardiologist
Blackrock Clinic,
Dublin 4

Chris Watson
School of Medicine and Medical Science,
The Conway Institute,
University College Dublin
Dublin 4

Fiona Ryan, PhD, MPSI
Research Pharmacist
St Michael's Hospital,
Dun Laoghaire
Co Dublin

Jonathan Dodd, MD, FRCPI
Consultant Radiologist
Radiology Department, St Vincent's
University Hospital, Dublin 4.

Document Type: Protocol

Document Number: HBT-PTCL-01**.** Version 10

Preparation Date: 17 February 2021

Approval Date (Investigator and Sponsor): 18/02/2021

Effective Date: 18/02/2021

EudraCT Number: 2015-002928-53

4. SIGNATURES

INVESTIGATOR APPROVAL SIGNATURES

I agree to conduct the study outlined above according to the terms and conditions of the protocol, GCP and applicable regulatory requirements. All information pertaining to the study will be treated in a confidential manner.

Prof Ken McDonald (M.D., F.R.C,P.I)	Consultant Cardiologist and Director of Cardiovascular Research, St Vincent's University Hospital, Clinical Professor, School of Medicine, University College, Dublin, Ireland		18/02/2021
**Name**	**Position**	**Signature**	**Date**
Prof Mark Ledwidge (BSc (Pharm), PhD, MPSI)	Research Director, Heart Failure Unit, St Vincent's University Hospital, UCD Professor, School of Medicine, University College, Dublin, Ireland		18/02/2021
**Name**	**Position**	**Signature**	**Date**

SPONSOR APROVAL SIGNATURES

I agree to support the study outlined above according to the terms and conditions of the protocol, GCP and applicable regulatory requirements. All information pertaining to the study will be treated in a confidential manner.

Prof Mark Ledwidge (BSc (Pharm), PhD, MPSI)	Co-founder and Research Director The Heartbeat Trust		18/02/2021
**Name**	**Position**	**Signature**	**Date**

5. CONFIDENTIALITY STATEMENT

This document contains confidential information that must not be disclosed to anyone other than the Sponsor, the funder, the investigative team, regulatory authorities and member of the Research Ethics Committee.

TABLE OF CONTENTS

1. STUDY TITLE ... 1
2. STUDY SPONSOR ... 1
3. APPLCIATION DETAILS ... 2
4. SIGNATURES ... 3
5. CONFIDENTIALITY STATEMENT ... 3
6. DOCUMENT HISTORY ... 7
7. SYNOPSIS ... 16
8. ABBREVIATIONS ... 20
9. INTRODUCTION ... 22
9.1 Overview and hypothesis ... 22
9.2 Background information ... 22
9.3 Rational for the study ... 23
10. OBJECTIVES AND ENDPOINTS ... 26
10.1 Objectives ... 26
10.2 Endpoints ... 27
11. TRIAL DESIGN ... 32
11.1 General considerations ... 32
11.2 Selection of study population ... 33
11.2.1 Inclusion criteria ... 33
11.2.2 Exclusion criteria ... 34
12. STUDY ASSESSMENTS AND PROCEDURES ... 35
12.1 Screening visit (Visit 1) ... 35
12.2 Baseline visit (Visit 2) ... 36
12.3 Follow up visits (Visits 3-11) ... 37
12.4 Assessments ... 40
12.4.1 Laboratory parameters ... 40
12.4.2 eGFR assessments ... 40
12.4.3 Second messenger cGMP (urine) ... 40
12.4.4 Biomarkers ... 40
12.4.8 Medical history ... 41
12.4.9 Demographics ... 41
12.4.10 Physical exam ... 42
12.4.11 Vital signs ... 42
12.4.12 Height, weight and waist/hip circumference ... 42
12.4.13 Ambulatory blood pressure monitoring (ABPM) ... 42
12.4.14 Pregnancy test ... 42

12.4.15 Electrocardiography (ECG) 43

12.4.16 Echo-Doppler studies 43

12.4.17 Cardiac magnetic resonance imaging (cMRI) 44

12.4.18 Concomitant medications. Potential drug interactions with LCZ696 and/or valsartan therapy 45

12.4.19 Questionnaires 48

13. STUDY DRUG 48

13.1 Blinding 48

13.2 Study drug supply and storage 49

13.3 Accountability of the study drug 50

13.4 Dispensing the study drug 50

13.5 Assessment of compliance 51

13.6 Overdose of study treatment 51

13.7 Permitted study drug dose adjustment and interruptions 52

13.8 Study drug restart after temporary treatment interruption 52

13.9 Study drug discontinuation and premature withdrawal of subjects 53

13.10 Emergency unblinding of treatment assignment 54

13.11 Definition of end-of-trial 54

14. SAFETY REPORTING 55

14.1 Definitions 55

14.2 Adverse event assessment 57

14.3 Emergency unblinding procedures 58

14.4 Follow up of adverse events 58

14.5 Documentation of adverse events by the Investigator 59

14.6 Reporting procedures for SAEs to the Sponsor 60

14.7 Reporting procedures for SUSARs to the EC and Regulatory authorities 60

14.8 SAE reporting to Novartis 61

14.9 Annual reports and line listings 62

15. PROTOCOL ADHERENCE 62

15.1 Protocol Amendments 62

16. STATISTICS 63

16.1 Description of statistical methods 63

16.2 Determination of sample size 63

17. DIRECT ACCESS TO SOURCE DATA/DOCUMENTS 64

18. DATA HANDLING AND RECORD KEEPING 64

18.1 Data collection, source documents and case report forms (CRFs) 64

18.2 Data reporting 65

19. RETENTION OF ESSENTIAL DOCUMENTS 65

20. QUALITY CONTROL AND QUALITY ASSURANCE PROCEDURES 65

20.1 Before the study 65
20.2 During the study 65
21. AUDITS AND INSPECTIONS 67
22. ETHICAL CONSIDERATIONS 67
22.1 Ethical conduct of the study 67
22.2 Declaration of Helsinki 68
22.3 Good Clinical Practice 68
22.4 Approvals 68
22.5 Informed consent 68
22.6 Subject confidentiality 70
23. FINANCING AND INSURANCE/INDEMNITY 70
24. CLINICAL STUDY REPORT AND PUBLICATION POLICY 70
24.1 Clinical study report 70
24.2 Publication policy 70
25. STUDY PLANNING AND SCHEDULE 71
25.1 Timelines and study duration 71
26. APPENDICES 72
Appendix 1. Montreal Cognitive Assessment (MoCA) 72
Appendix 2. Standardised Mini-Mental State Examination SMMSE) 72
Appendix 3. EuroQoL (EQ-5D-5L) Health Questionnaire 73
Appendix 4. Montreal Cognitive Assessment (MoCA) Version 3. Administration and Scoring Instructions 75
Appendix 5. Adverse Event Handling. Investigator Responsibilities. Flow Diagram 79
Appendix 6. Adverse Event Handling. Sponsor Responsibilities. Flow Diagram 80
Appendix 7. Clinically notable laboratory values and vital signs 81
Appendix 8. Treatment guidelines 82
REFERENCES 86

6. DOCUMENT HISTORY

Document	Approval date	Summary of Change
Version 1.0	27 August 2015	New document
Version 2.0	16 October 2015	Protocol has been updated to reflect the recommendations/queries of the HPRA and EC review of the initial CTA. The changes are as follows:<br>Exclusion criteria to include women of childbearing potential not using adequate contraception. Definition of postmenopausal added. Contraception must be continued for one week following discontinuation of study drug.<br>A pregnancy test (urinary dipstick) will be carried out in women of childbearing potential at all timepoints<br>Exclusion criteria has been extended to exclude patients with SBP <100mmHg<br>MMSE and MoCA questionnaires previously referred to as QOL questionnaires. This has been updated to refer to them as cognitive assessments.<br>MMSE and MoCA will also be administered at 5 years<br>Concomitant medication section (12.4.18) has been updated with details of furosemide, PDE inhibitors, metformin and statin interactions<br>Details of SAE reporting to Novartis has been updated (Appendix 8 and Section 14.8)<br>Section 22.5 (Informed Consent) updated to include IC for biobanking blood samples<br>Appendix 10 has been updated with more precise instruction for hypotension management<br>Section 12.4.17 updated to state that if a patient had a cMRI in the past 6 weeks there is no need to repeat the MRI at baseline.<br>Reference to Blackrock Clinic is Section 12.4.17 has been removed.<br>Section 14.2 updated to cross-reference to the section of the IB that should be used to evaluate expectedness of an adverse reaction.<br>Section 12.4.19 updated to state that any illnesses identified following the administration of a questionnaire should be managed appropriately.

Version 3.0	22 Jan 2016 (Substantial amendment)	Reference to the Blood Pressure Unit has been changed to the STOP-HF Service throughout the document.<br><br>Section 11.2.2. Exclusion criteria no 11 updated to 'malignancy or presence of any other disease with a life expectancy of < 2 years<br><br>Section 12.2. LCZ696 and valsartan administration. 'Twice daily' inserted.<br><br>Table 2 updated. Glucose monitoring deleted; cGMP measurement at 4/52 and 3/12 also<br><br>Section 12.4.1. Glucose testing deleted.<br><br>Section 12.4.3. Additional two time points added for cGMP at 4/52 and 3/12<br><br>Section 12.4.5. NT-proBNP will be measured by St Vincent's Hospital Group laboratory as per standard hospital policy and not using a point-of-care assay.<br><br>Section 12.4.12. Description of how to measure waist/hip ratio inserted.<br><br>Section 12.4.19. Standardised MMSE used instead of MMSE<br><br>Section 13. Reference to HBT-GCP-SOP-17 'Study Drug Management for the PARABLE Study' inserted. Reference to the distribution company Almac inserted. Reference to clinical trials pharmacist replaced by investigator site.<br><br>Section 13.10. Reference to HBT-GCP-SOP-17 'Study Drug Management for the PARABLE Study' inserted which describes the unblinding process<br><br>Section 25.1. Timelines updated<br><br>Appendix 2. Standardised MMSE inserted
Version 4.0	10 May 2016 (Substantial amendment).	Section 7.0 and section 11.2.1. NP cut-off values for inclusion criteria have been amended as follows:<br><br>Elevated NP: BNP between 35 and 280pg/ml or NT-proBNP values between >125 pg/ml and 1,000 pg/ml within 6 months prior to screening<br><br>Section 11.2.1, 12.1, 12.4.5 and Table 2. An elevated NP level in the six month period prior to screening is acceptable.<br><br>Section 11.1, 12.1, 12.2, Figure 1a and 1b. Requirement for a 36 hour washout period for patients on current ARB therapy is not required. The requirement for 36 hour washout period for an ACEI remains the same.<br><br>Section 12.1 and 22.5. Informed consent may only be taken by a qualified medical doctor.<br><br>Table 2. Requirement for measurement of alk phos at baseline has been removed.<br><br>Section 12.4.3 and Table 2. Measurement of cGMP changed from 4 week timepoint to 6 week timepoint.<br><br>Section 12.1. Patients may also contact the STOP-HF clinic directly in response to an advertisement. Patients may also be referred from other clinics, hospital departments or healthcare professionals.

		Table 2. Additional row added for Natriuretic peptide (previously included with biomarkers)<br>Table 2. Biomarker measurement at 2 weeks and 4 weeks has been removed.
**Version 5.0**	**01 Nov 2016**	
**Substantial amendments (HPRA approval: 19 Dec 2016; EC approval: 08 Dec 2016**		
**Section 11.2.1.** Key Exclusion Criteria updated with new BNP and NT-proBNP cut-offs as follows:<br>Elevated NP: BNP between 20 and 280pg/ml or NT-proBNP values between 100 pg/ml and 1,000 pg/ml within 6 months prior to screening.<br>Asymptomatic left ventricular systolic dysfunction defined as LVEF <50% on most recent measurement.		
**Non-substantial amendments**		
**Section 7.0.** Eligibility criteria No 3. Added ‘or at screening’<br>**Section 11.1.** Added ‘discontinuation of ARB (no washout necessary)’.<br>**Section 11.2.1.** Inclusion criteria.<br>Change to BNP and NT-proBNP cut-offs<br>Added ‘or at screening’ to criteria no 3.<br>**Section 12.1.** Screening visit<br>Added that initial contact with patient must be documented in the medical notes. Updated NP cut-off values.<br>**Section 12.2.** Changes made to the titration dosages.<br>Clarification that a 36h washout is needed for ACEI only.<br>**Section 12.3.** Clarified that a ‘paper’ CRF is being used.<br>**Table 2.** Randomisation changed from Visit 1 to visit 2.<br>Column added ‘Where to record source data’<br>Row added to allow for visit ‘windows’<br>**Section 12.4.1.** Added reference to Laboratory SOP<br>**Section 12.4.5.** Added ‘or at screening’<br>**Section 13.** Added ‘LCZ696, Valsartan and matching placebos are provided by Novartis Ireland Ltd to the distributor company, Almac’. Clarified scope of SOP-17.<br>**Section 13.2.** Added that the investigator will notify sponsor when stocks of study drug are running low and that it is the responsibility of the sponsor (not investigator) to re-order study drug. Study drug will be delivered directly to STOP-HF service, and not pharmacy department.<br>**Section 13.3.** Replaced ‘drug accountability ledger’ with HBT-GCP-FM-22; ‘Study Drug Inventory and Subject Assignment Log’.<br>Added that ‘All patient returns should be recorded in the CRF and in the Study Drug Returns Log (HBT-GCP-FM-23)’.<br>**Section 13.4.** Added that study drug is provided to the site labelled. Added that dispensing records should be maintained in the drug accountability log (HBT-GCP-FM-22; ‘Study Drug Inventory and Subject Assignment Log’<br>**Section 13.10.** Added that the CTPM should be contacted in the event of an unblinding requested, and WebEz Helpdesk as a backup.		

Section 13.11. Changed 'Chief' investigator to 'Principal investigator'.

Section 14. The Adverse Event Report Form is no longer an appendix in this protocol. Therefore, reference is made to the Form in the CRF and not appendix 5.

Update made to the location of the EMA IME list.

The Serious Adverse Event Report Form is no longer an appendix in this protocol. Therefore, reference is made to the QMS number of the form.

Section 14.2. Causality should be made assuming study drug is LCZ696.

Terms for causality modified slightly to reflect terminology used in AE Form (Possible and Probably instead of possibly and probably).

Added 'SmPC for valsartan' to the assessment of expectedness section.

Changes made to the 'action taken' terminology to reflect terminology in SAE form.

Section 14.6. No need to notify the sponsor immediately of an SAE.

Responsible Person for Pharmacovigilance replaced with Clinical Trial Project Manager.

Section 14.7. Back-up measure to SUSAR reporting during periods of non-availability of CTPM added (i.e. direct reporting to HPRA)

Section 14.9. Email address for Novartis notification of SAEs updated.

Section 15. SOP on Deviation and Protocol Violation referenced.

Section 22.5. Last paragraphs added to state that the informed consent process must be documented in the patients' medical notes.

Section 25.1. Timelines of study updated.

Appendices:

Adverse Event Report Form removed from Appendix.

Serious Adverse Event Report Form removed from Appendix.

Appendix 5. Immediate reporting to sponsor removed. Medical sign-off of SAE form added.

**Version 6.0**	**Approval date: 04 Sept 2017. Effective date: 31 Oct 2017**

Substantial amendments

Section 10.1. Safety has been removed as an exploratory objective and added as a secondary objective.

Section 10.2. Safety has been removed as an exploratory endpoint and added as a secondary endpoint.

Section 10.2. The first endpoint (relating to cMRI LAVI and 9 months) has been removed.

Section 10.2. The second endpoint (relating to cMRI parameters of left ventricular structure and function) has been adjusted to remove assessment at 9 months.

Section 10.2. Secondary endpoint to also include change in BNP.

Section 12.4.17. MRI will only be performed at baseline and 18 months (9 month MRI removed).

Section 12.4.17. St Vincent's University Hospital has been added as a site for cMRI.

Section 12.4.17. 'Use of gadolinium containing contrast' has been removed and reference to gadolinium has been deleted.

Non-substantial amendments

Section 12.1. The following text was deleted as it does not relate to the screening visit 'If the patients is taking an ACE inhibitor, they will be instructed to stop taking this medication 36 hours before visit 2)'.

Table 2. The 'Where to record source data' column was updated (to reflect that the CRF can be used as the source document for some data).<br>Table 2. Footnote added 'Baseline echo must be performed within 90 days prior to baseline'.<br>Section 12.4.4. The speed, duration and temperature settings on the centrifuge were updated.<br>Section 12.4.16. Text added 'A baseline echo must be performed within 90 days prior to baseline visit'.<br>Section 13.10. The WebEz Helpdesk must be contacted with a request for emergency unblinding (previously the clinical trial project manager could be contacted).<br>Section 14.1. The following text was added 'Abnormal signs and symptoms identified during the physical and cardiac exam may also constitute an adverse event it they were not present at baseline'.<br>Section 14.1. The note to refer to the EMA IME list for adverse event assessment of seriousness was deleted.<br>Section 14.8. The email address of the Novartis pharmacovigilance department was updated.<br>Section 15. All deviations will now be recorded in a Deviation Log according to SOP (GBT-GCP-FM-28).<br>Section 25.1. The study timelines were updated.	
**Version 7.0**	**Approval date: 27 November 2018. Effective date: 01 March 2019**
**Substantial amendments**	
Section 10.1. Secondary objectives. The following objective was added:<br>To assess response to LVZ696 based on genetic variants of the NPPB, NPRA and NPRC genes (e.g. rs5068, rs198389).<br>Section 10.1. Exploratory objectives. The following objectives were added:<br>To assess the difference between groups in terms of atrial fibrillation measured non-invasively over 5-7 days using Holter monitoring<br>To assess response of immune cells to sacubitril/valsartan<br>Section 10.2. Secondary endpoints.<br>The following was added to bullet point 4. e.g. ejection fraction, Lateral/medial e' and lateral/medial/average E/e'.<br>The following were added as secondary endpoints:<br>Response to sacubitril/valsartan based on genetic variants of the NPPB, NPRA and NPRC genes (e.g. rs5068, rs198389);<br>Change in the numbers of patients with progression of left ventricular dysfunction, defined as: ejection fraction <50% with change of >5%; and/or lateral E/e' >13 or average E/e' >14 (with change >2); and/or lateral e'<9, with reduction >2.<br>Section 10.2. Exploratory endpoints: The following were added:<br>Difference between groups in terms of immune system response, e.g. CD14+, CD16+/- monocyte RNA expression profile;<br>Difference between groups in terms of atrial fibrillation measured non-invasively over 2-7 days using Holter monitoring.<br>Section 11.2.2. Exclusion criteria No. 14 amended so that raised LFTs are limited to AST and ALT.<br>Section 16.1. Description of statistical methods. The tests to be used to analyse continuous variables are described depending on their ability to be transformed or not. A plan for missing data is included. A description of how demographic and clinical characteristics will be presented is outlined. Further detail is	

given on how to analyse primary and secondary outcomes and the variables to be considered when adjusting.<br><br>Section 16.2. Determination of sample size. The following was added: In a review of the expected effect size, once blinded echocardiography was available for 125 patients at the 9-month time point, it was noted that the actual standard deviation of baseline to 9-month change scores was 4.7. This means an expected effect size of at least (2.0/5.0) = 0.40. An effect size of 0.4 would require 96 patients in each group, suggesting that the study is adequately powered on LAVI assessed by echocardiography. In addition, the study has at least 80% power with a two-tailed alpha = 5% to detect a 6 g/m2 difference in LVMI change by CMRi, a 2.5 mL/m2 in LAVI change by CMRi, a 2-unit difference in E/e' or e' using Tissue Doppler measurements.<br><br>Section 25.1. Timelines and study duration. The LPFV and LPLV dates were updated.<br><br>Section 7.0. Synopsis. Updated to reflect the changes to the various sections of the protocol.	
**Non-substantial amendments**	
Section 4.0. Signatures. Affiliation added to KMD and ML positions. ML title changed.<br><br>Section 9.2. Background. The full meaning of the STOPHF acronym was included. The AHA guideline was updated from 2014 to 2017. The following was added "In the STOP-HF follow up study, we show that the minor C allele of genetic variant rs198389 of the *Nppb* gene is associated with sustained elevated circulating levels of BNP and reduced incidence of left ventricular dysfunction over a five year follow up period".<br><br>Section 9.3. Rationale. Added 'This may be mediated through impacts on the innate immune system and monocyte-derived-macrophage phenotypes in the myocardium'.<br><br>Section 12.1. Screening visit. The interval between the screening visit and baseline visit has been clarified and wording changed to "An appointment for Visit 2 is scheduled for up to two weeks later".<br><br>Section 12.4.11. Vital signs. Clarification that the cuff BP (and not 24h ABPM) will be used to confirm eligibility and inform dosing decisions.<br><br>Section 16.2. Determination of sample size. A reference for the sample size was added.<br><br>Table 2. The second column from the left (Where to record source data) was deleted as there is a Source Documentation SOP that details this information.	
**Version 8.0**	**Approval date: 17 April 2019. Effective date: 01 July 2019**
**Substantial amendments (9-month follow-up study)**	
Table 2. An additional column was added for the 9-month follow-up visit (visit 12)<br><br>Appendix 9 added. Details of sub-study described	
**Minor amendments**	
Amend typographical error from "To assess response to LVZ696 based on genetic variants of the NPPB, NPRA and NPRC genes (e.g. rs5068, rs198389)" in Protocol Version 7.0 Summary, page 13, and Objectives section, page 23, to reflect the endpoint defined in Protocol Version 7.0: i.e. "To assess response to LVZ696 based on genetic variants associated with the expression and metabolism of BNP (e.g. neprilysin, *Nppb, Npra and Nprc genes)."*	

Version 9.0	Approval date: 06 October 2020. Effective date: 06 October 2020
**Substantial amendments**	
Section 9 'Background'. Inserted new publication (reference 6) supporting the study hypothesis showing potential benefit of NP augmentation on atrial electrophysiology, structure and function.<br><br>Section 10.2 'Endpoints'. Final changes, clarifications and additions to secondary and exploratory endpoints, linked to new publication (reference 6) have been made in this section of the protocol prior to completion of the study.<br><br>Section 12.4.4. The list of blood biomarkers of fibrosis, inflammation, metabolism, platelet function and coagulation have been updated.<br><br>The following biomarkers have been added to the table in this section: Col1A1, MMP-3, TIMP-4, PRA, aldosterone, IL-1R1, IL-R2, IL-2RA, IL-6RA, ANP, IL-17, GP6, uPAR, TR-AP, CD163, E/P-Selectins, uPA, vWF, TRAP, Cystatin B, PON3, TR-P1.<br><br>Sections 12.4.6 (MMP2 and TIMP-1) and 12.5.7 (other biomarkers) were deleted. These sections described in detail the assays to be used to analyse the biomarkers. This was replaced with the following statement: [Biosamples]…will be transported on dry ice to the Conway Institute, University College Dublin for simultaneous analysis of selected biomarkers from the table below using validated analytical techniques.	
**Version 10.0**	**Approval date: 17/02/2021. Effective date: 18/02/2021**
**Substantial amendments**	
<u>Section 10.1 'Objectives' amended as follows:</u><br><br>The list of objectives has been numbered (previously bullet points were used). The following changes were made to the list of <u>secondary objectives:</u><br><br>• Secondary objective #2 was added "To assess the impact of LCZ696 versus valsartan therapy on cardiovascular and non-cardiovascular adverse events, including prior to and post onset of Covid19 pandemic".<br>○ Justification: The impact of the interventions on clinical adverse events has previously been designated a secondary endpoint measure. Furthermore, the overall cardiovascular adverse clinical event rate per participant/year is 4 times higher in PARABLE than the original STOP-HF study, justifying its separate evaluation. Finally, it is important to evaluate adverse clinical events prior to the onset of Covid19 and post-onset of Covid19.<br><br><u>Exploratory objectives:</u><br><br>• Exploratory objective #2 updated to include any atrial or ventricular arrythmia (and not just atrial fibrillation)<br>• Exploratory objective #4 was removed to avoid duplication as this is now incorporated into secondary objective #2.<br>• Exploratory objective #4 added to evaluate if the treatment effects observed during the 18-month period persist nine-months after study drug discontinuation (9 month follow-up substudy)<br><br><u>Section 10.2 'Endpoints' (EP) amended as follows:</u><br><br>The list of EPs has been numbered (previously bullet points were used).<br><br>The following <u>secondary endpoints</u> were updated:<br><br>Secondary endpoints:<br><br>• EP #1 order updated: Left ventricular function using Doppler Echocardiography (average E/e') between baseline and 18 months	

- EP #2 order updated: Left atrial volume index (Doppler Echocardiography LAV)/BSA* between baseline and 9 months
- EP #3 order updated: Left atrial function measured as total cMRI LAEF ((LAVimax-LAVimin)/LAVimax) over 18 months
- EP #4 order updated Left atrial function measured as cMRI left atrial stroke volume index (LAVmax-LAVmin)/BSA*, or LAVimax-LAVimin over 18 months
- EP #5 order updated Left ventricular structure (cMRI LVMi indexed to BSA*) over 18 months
- EP #6 order updated Left ventricular function (cMRI LVEF) over 18 months
- EP #7 updated Measures of vascular compliance (ABPM pulse pressure) between baseline and 18 months
- EP #8 order updated Change in log transformed NT-proBNP between baseline and 18 months
- EP #9 updated Time to first all cardiovascular death and major adverse cardiac events (MACE) requiring hospitalisation over 18 months. MACE includes arrthymia (including atrial fibrillation/flutter), transient ischaemic attack, stroke, valvular heart disease, myocardial infarction, peripheral or pulmonary thrombosis/embolus or heart failure
- EP #10 added primary endpoint analysed per protocol

The following <u>exploratory endpoints</u> were updated/reordered:

Exploratory endpoints:

- EP #11 added to include left atrial function measured as left atrial cardiac index (LAVimax-LAVimin) x heart rate
- EP #12 updated Left atrial function as cMRI myocardial strain measured using feature tracking between baseline and 18 months
- EP #13 added Left ventricular structure (as cMRI LVESV, LVEDV, Fractal Analysis), all indexed to BSA* between baseline and 18 months
- EP#14 Left ventricular function, measured as cMRI left ventricular stroke volume index (LVESVi-LVEDVi) between baseline and 18 months
- EP#15 Left ventricular function, measured as cMRI left ventricular cardiac index ((LVESVi-LVEDVi) x heart rate) between baseline and 18 months
- EP#16 Left ventricular function as cMRI myocardial strain using feature tracking between baseline and 18 months
- EP#17 Left ventricular function as cMRI e'
- EP#18 Evaluation of treatment effects on cMRI measures of right atrial and structure and function (RAVI, RAEF, RVEF, SV, CI, E', E/e')
- EP#19 Left atrial function using Doppler Echocardiography (E/A ratio)
- EP#20 Left ventricular structure using Doppler Echocardiography (LVMI, LVESVI, LVEDVI)
- EP#21 Left ventricular function using Doppler Echocardiography (LVEF, stroke volume (SV), cardiac index (CI), Lateral or medial e' and lateral or medial E/e')
- EP#22 Change in log transformed BNP, NTproBNP/BNP ratio and other related peptides, e.g. ANP, urinary cGMP between baseline and 9 months and baseline and 18 months
- EP#23 Change in the following parameters of 24-hour ABPM between baseline and 9 months and baseline and 18 months: Systolic blood pressure, diastolic blood pressure and heart rate
- EP#24 Change in resting (clinic measured) systolic blood pressure, diastolic blood pressure and heart rate between baseline and 9 months and baseline and 18 months.
- EP#25 Incidence of all cardiovascular death and MACE requiring hospitalisation over 18 months.
- EP#26 Incidence of all cardiovascular related serious adverse events (SAEs) over 18 months
- EP#27 Incidence of all-cause death and SAEs requiring hospitalisation over 18 months
- EP#28 Incidence of all SAEs over 18 months
- EP#29 Incidence of all-cause adverse events (AEs) by system organ class over 18 months

- EP#30 Time to first event analysis using Kaplan Meier survival curves for cardiovascular death or MACE requiring hospitalisation, cardiovascular-related SAEs, death or all-cause emergency hospitalisations, all-cause SAE.
- EP #33. Impact of LCZ696 on endpoints dependent on BNP above or below 50pg/ml has been added to this endpoint which already includes median BNP, NTproBNP and NTproBNP/BNP ratio at baseline
- EP #34. Correlations between Echo and MRI measures of atrial and ventricular structure and function added
- EP #36. CES, CYP2C9, NPRA and NPRB added to the list of genetic variants
- EP #37. Additional biomarkers of inflammation added
- EP #38. Change in creatinine, albumin creatinine ratio and cGMP added to list of urinary biomarkers
- EP #39. Two additional correlations added to this section: Measures of left atrial and structure and function (LAVI, LAEF, LVEF, SV, CI, E', E/e') and pericardial and epicardial fat; Measures of right atrial and structure and function (RAVI, RAEF, RVEF, SV, CI, E', E/e') and pericardial and epicardial fat.
- EP #40,41 added (MoCA, MMSE and EuroQol data separated out into three separate EPs)
- EP #45. Atrial fibrillation/flutter, paroxysmal supraventricular tachycardia and other atrial or ventricular arrthymias added to this EP instead of paroxysmal atrial fibrillation and atrial tachycardia (clarification of language)
- EP #46. Polygenic risk score of atrial fibrillation and atrial dilatation added to this EP. KLKB1 added to the list of gene variants
- EP #47. Sub-group analyses prespecified
- EP #48. Use of circulating metabolomics to identify responders to LCZ696 added
- EP #50. An endpoint was added to evaluate all primary and secondary endpoints in the total follow-up period and prior to Covid19 restrictions implemented (13 March 2020).
- EP #52. An endpoint to capture health economics (cost-benefit and cost-effectiveness analysis) added

Section 12.4.3. ANP added to cGMP in this section on urinary biomarkers.

Section 25.1. Study end date updated

Appendix 9. This list of endpoints for the 9-month follow-up study have been deleted and replaced with a reference to the objectives and endpoints outlined in Section 10.1 and 10.2 of the protocol.

7. SYNOPSIS

**Title of study**	Personalised prospective comparison of ARni with ArB in patients with natriuretic peptide eLEvation (PARABLE)
**Name of Sponsor**	The Heartbeat Trust
**Phase of development**	Phase II
**Hypothesis**	We hypothesise that, in a phase II study design, the relative impact of LCZ696 and valsartan on left atrial volume index will be different in at-risk, asymptomatic patients with elevated natriuretic peptide over 18 months.
**Objectives**	Primary:<br>To assess the impact of LCZ696 versus angiotensin II receptor blocker (ARB) (valsartan) on left ventricular diastolic function over 18 months.<br><br>Secondary:<br>1. To assess the impact of LCZ696 versus valsartan therapy on atrial and ventricular structure and function over 9 and 18 months.<br>2. To assess the impact of LCZ696 versus valsartan therapy on cardiovascular and non-cardiovascular adverse events, including prior to and post onset of Covid19 pandemic.<br>3. To assess the impact of LCZ696 versus valsartan on biochemical markers of fibro-inflammation and myocardial damage at 9 and 18 months.<br>4. To assess the response to LCZ696 based on genetic variants of associated with the expression and metabolism of BNP (e.g. neprilysin, Nppb, Npra and Nprc genes)<br>5. To assess the impact of LCZ696 versus valsartan on biochemical markers of renal function.<br>6. To correlate the impact on left ventricular dysfunction and biochemical markers in order to understand the mechanism of benefit of LCZ696 versus ARB therapy in modulating fibro-inflammation, myocardial damage, and cardiac function.<br>7. To provide further information in the target population on the safety and tolerability of LCZ696 versus valsartan.<br><br>Exploratory:<br>1. To assess the impact of LCZ696 versus valsartan on Health-Related Quality of Life and cognitive function in the target population.<br>2. To assess the response to therapy in terms of atrial and ventricular arrthymia measured non-invasively over 5-7 days using Holter monitoring<br>3. To assess the response of immune cells to sacubitril/valsartan<br>4. To evaluate if the treatment effects observed during the 18-month PARABLE study persist nine-months after study drug discontinuation
**Trial design**	This is a single centre, parallel design, randomised, double blind, double dummy, phase II trial in asymptomatic patients at risk of heart failure with elevated levels of natriuretic peptide.

**Key inclusion criteria**	To be eligible for inclusion, each subject must meet the following criteria at the screening visit:<br>1. Age > 40yrs with cardiovascular risk factor(s) including at least one of:<br>a. History of hypertension (medicated for greater than one month);<br>b. History of diabetes;<br>2. Elevated NP: BNP between 20 and 280pg/ml or NT-proBNP values between 100 pg/ml and 1,000 pg/ml within 6 months prior to screening or at screening<br>3. LAVI > 28 mL/m^2 obtained during Doppler Echocardiography within 6 months prior to screening or at screening<br>4. Subjects must give written informed consent to participate in the study and before any study related assessments are performed.
**Key exclusion criteria**	1. A history of heart failure<br>2. Asymptomatic left ventricular systolic dysfunction defined as LVEF <50% on most recent measurement<br>3. Systolic blood pressure <100mmHg<br>4. Persistent atrial fibrillation<br>5. Subjects' with a history of hypersensitivity, allergy or intolerance to LCZ696, ARB or neprilysin therapy or to any of the excipients or other contraindication to their use<br>6. Previous history of intolerance to recommended target doses for ARBs<br>7. Subjects who require treatment with both an ACE inhibitor and an ARB<br>8. Presence of haemodynamically significant mitral and /or aortic valve disease<br>9. Presence of hemodynamically significant obstructive lesions of left ventricular outflow tract, including aortic stenosis<br>10. Conditions that are expected to compromise survival over the study period<br>11. Serum potassium level > 5.2 mmol/L<br>12. Severe renal insufficiency (eGFR <30 mL per minute per 1.73 m^2).<br>13. Hepatic dysfunction (AST or ALT values exceeding 3 x Upper Limit of Normal (ULN)]<br>14. Concomitant use of aliskiren<br>15. History of angioedema<br>16. History or evidence of drug or alcohol abuse within the last 12 months<br>17. Malignancy or presence of any other disease with a life expectancy of < 2 years<br>18. Women who are pregnant, breast-feeding, or women of childbearing potential not using estro-progestative oral or intra-uterine contraception or implants, or women using estro-progestative oral or intra-uterine contraception or implants but who consider stopping it during the planned duration of the study. A postmenopausal state is defined as no menses for 12 months without an alternative medical cause. (Contraception must be continued for one week following discontinuation of study drug).<br>19. Concomitant participation in other interventional clinical trials<br>20. Participation in any investigational drug trial within one month of visit 1<br>21. Refusal to provide informed consent<br>22. Subjects with contraindications to MRI

	23. Any surgical or medical condition which might significantly alter the pharmacokinetics of study drugs
**Number of subjects**	Planned: A total of 250 subjects are to be included in the treatment period
**Test product, dose and mode of administration**	Included subjects will be randomised into one of two groups:<br>• LCZ696 (and matching placebo)<br>• Valsartan (and matching placebo)<br><br>All treatments will be administered in tablet form, orally, twice daily
**Duration of treatment**	18 months
**Statistical methods**	If continuous variables are transformable to normal, they will be power-transformed and independent, two sample t-tests will be used for analysis of continuous variables. If data are not transformable to normal, non-parametric t-test equivalents (e.g. Wilcoxon signed rank and rank sum test, Mann-Whitney test and analysis of covariance (ANCOVA)) will be used. Chi-squared (or Fisher Exact) analyses will be used to compare categorical variables. If required, missing data will be addressed using multiple imputation using chained equations as previously described (Ledwidge et al. JAMA. 2013;310(1):66-74) and results will be presented (primary and secondary end points) as pooled results from the multiple imputed data sets using a last-observation carried-forward analysis.<br><br>Demographics and clinical characteristics will be summarized with descriptive statistics (counts and percentages for categorical variables, or mean value ± the standard deviation for normally distributed continuous variables, or medians and interquartile ranges for non-normally distributed continuous variables). Repeated marker changes from baseline to 18 months will be analysed using ANOVA with repeated measures models.<br><br>Primary and secondary outcome measures will be performed both with and without adjustment for the effects of age, sex, diabetes, hypertension, obesity, and vascular disease. Further models will include adjustment for baseline measures of the outcome of interest (e.g. LAVI, BNP). Categorical endpoints will be analyzed using generalized linear modeling with a binomial outcome distribution for prevalence and a Poisson outcome distribution for incidence rate (adjusted for patient-years). The multivariable models will include both prespecified covariates (age, sex, diabetes, hypertension, obesity, and vascular disease) and those variables with univariate P values ≤ 0.25. The likelihood ratio test and the Nagelkerke R Square will be used to identify independent variables with low explanatory power and to assess the fit of each specification of the multiple regression models.
**Sample size**	Based on the endpoint of change in LAVI over 18 months, the PARABLE trial aims to recruit 250 subjects (125 per arm). Sample size estimates were calculated from the current STOP-HF subject database (n=1499). A subsample of subjects with BNP > 50 pg/mL, LAVI> 28 mL/m^2, with normal ejection fraction (≥ 50%) and in normal sinus rhythm was drawn. This resulted in a population of 163 (11%) of the total with a population BNP of 125 pg/mL and a baseline mean LAVI of 36.2 mL/m^2 (standard deviation of 6.9 mL/m^2) determined by Doppler Echocardiography. This LAVI is reassuringly similar to the baseline level observed by Solomon et al. (Lancet 2012; 380: 1387–95) in the PARAMOUNT study, which observed a 36-week drop in LAVI of 2.6 mL/m^2. Assuming the control group-shows no decrease in LAVI (as in Solomon et al. 2012 paper), the expected effect size in a similar study is 2.6/6.5 = 0.38. Setting alpha = 5% (two-tailed),

	beta = 20% and using an independent t-test to assess the change in LAVI over follow-up, the required number of subjects to detect this effect is 222 (111 per study arm), or 248 after accounting for a 10% dropout rate.<br><br>In a review of the expected effect size, once blinded echocardiography was available for 125 patients at the 9-month time point, it was noted that the actual standard deviation of baseline to 9-month change scores was 4.7. This means an expected effect size of at least (2.0/5.0) = 0.40. An effect size of 0.4 would require 96 patients in each group, suggesting that the study is adequately powered on LAVI assessed by echocardiography. In addition, the study has at least 80% power with a two-tailed alpha = 5% to detect a 6 g/m^2 difference in LVMI change by CMRi, a 2.5 mL/m^2 in LAVI difference by CMRi, a 2-unit difference in E/e' or e' using Tissue Doppler measurements.

8. ABBREVIATIONS

**ABPM**	Ambulatory Blood Pressure Monitoring
**AC**	Atrial Cardiomyopathy
**ACE**	Angiotensin Converting Enzyme
**AE**	Adverse Event
**ALVDD**	Asymptomatic Left Ventricular Diastolic Dysfunction
**ANCOVA**	Analysis of Covariance
**ANOVA**	Analysis of Variance
**ARB**	Angiotensin Receptor Blocker
**ARNI**	Angiotensin Receptor Neprilysin Inhibitor
**BID**	Twice Daily
**BNP**	Brain Type Natriuretic Peptide
**BP**	Blood Pressure
**cGMP**	Cyclic Guanosine Monophosphate
**CRF**	Case Report Form
**EC**	Ethics Committee
**ECG**	Electrocardiography
**ECM**	Extracellular Matrix
**eGFR**	Estimated Glomerular Filtration Rate
**ELISA**	Enzyme-Linked Immunosorbent Assay
**EOS**	End of Study
**EQ-5D**	EuroQOL-5D
**EVCTM**	EudraVigilance Clinical Trial Module
**FBC**	Full Blood count
**FPFV**	First Patient First Visit
**GCP**	Good Clinical Practice
**GFR**	Glomerular Filtration Rate
**HPRA**	Health Products Regulatory Authority
**IL**	Interleukins
**IME**	Important Medical Events
**LAVI**	Left Atrial Volume Index
**LFT**	Liver Function Test
**LV**	Left Ventricular
**LVEF**	Left Ventricular Ejection Fraction
**MCP**	Monocyte Chemoattractant Protein
**MMP**	Matrix Metalloproteinase
**SMMSE**	Standardised Mini Mental State Examination
**MoCA**	Montreal Cognitive Assessment
**MRI**	Magnetic Resonance Imaging
**NP**	Natriuretic Peptide
**NSAID**	Non-Steroidal Anti-Inflammatory Drug
**NT-proBNP**	N-terminal of the prohormone brain natriuretic peptide
**PICP**	Procollagen Type 1 Carboxy-Terminal Propeptide

**PIIINP**	Procollagen III N-Terminal Propeptide
**PINP**	Procollagen Type I N-terminal Propeptide
**POC**	Point of Care
**RAAS**	Renin Angiotensin Aldosterone System
**SAE**	Serious Adverse Event
**SmPC**	Summary of Product Characteristics
**SOP**	Standard Operating Procedure
**SPSS**	Statistical Package for the Social Sciences
**SSAR**	Suspected Serious Adverse Reactions
**STOP-HF**	St Vincent's Screening TO Prevent Heart Failure
**SUSAR**	Serious Unexpected Serious Adverse Reaction
**TIMP**	Tissue Inhibitor of Matrix Metalloproteinase
**TNF**	Tumour Necrosis Factor
**U&E**	Urea and Creatinine
**ULN**	Upper Limit of Normal

9. INTRODUCTION

9.1 Overview and hypothesis

Elevated natriuretic peptide (NP) in asymptomatic patients is a protective, diuretic, natriuretic and anti-fibrotic response in patients with left ventricular dysfunction. The St Vincent's Screening TO Prevent Heart Failure (STOP-HF) study showed that NP screening and collaborative primary-secondary care can prevent cardiovascular diseases amongst at-risk patients. However, the intensification of pharmacological therapy applied in the STOP-HF study was largely focused on renin-angiotensin-aldosterone-system (RAAS) blockade and was non-specific to NP elevation.

LCZ696 (sacubitril/valsartan), is a first-in-class angiotensin receptor neprilysin inhibitor (ARNI). In addition to RAAS blockade, LCZ696 inhibits neprilysin resulting in reduced degradation of biologically active NPs. Previous work over 36 weeks has shown that LCZ696 in comparison with valsartan can provide greater reduction in left atrial volume index (LAVI), a continuous imaging surrogate of left ventricular diastolic dysfunction, when given to patients with heart failure.

We hypothesise that, in a phase II study design, the relative impact of LCZ696 and valsartan on LAVI will be different in at-risk, asymptomatic patients with elevated NP over 18 months.

9.2 Background information

An effective prevention strategy is critical if we are to curb the established epidemic of heart failure and cardiovascular disease. This is particularly important in the context of increasing community prevalence of left ventricular diastolic abnormalities associated with hypertension and diabetes and requires community diagnostics and targeted preventative therapies. New data demonstrate that grading risk beyond the presence of established risk factors can be individualized with NP assessment. Elevated NP in a population with established cardiovascular disease defines a group more prone to ventricular dysfunction, heart failure and other cardiovascular events and can be used to risk stratify asymptomatic populations, targeting those most likely to need intensive intervention and follow-up.

We have demonstrated in a prospective, randomised trial that NP-based screening and collaborative care with general practice can help target cardiovascular prevention and improve outcome in the St Vincent's Screening TO Prevent Heart Failure (STOP-HF) study [1]. This multi-faceted intervention involved community NP screening, improved use of RAAS modifying therapy, collaborative care with general practice and cardiovascular coaching to encourage adherence and attention to risk factor management. It demonstrated reduced development of left ventricular dysfunction, heart failure and other cardiovascular events requiring hospitalisation. This first of type study indicated that a biomarker driven strategy based on NP screening for heart failure prevention is feasible and has an impact across the wider expression of cardiovascular disease and has been incorporated into 2017 American Heart Association/American College of Cardiology guidelines.

As a unique, biomarker directed strategy for prevention of cardiovascular disease in patients without heart failure, the STOP-HF approach was ambitious and successful. It has been followed by a smaller, supportive study in diabetes [2]. New data from the study show that the intervention is cost-effective, potentially providing an important public health impetus for the STOP-HF biomarker driven approach to prevention.

However, while successful, the STOP-HF biomarker strategy lacks a specific pharmacological intervention linked to the screening biomarker, NP. In the STOP-HF follow up study, we show that the minor C allele of genetic variant rs198389 of the *Nppb* gene is associated with sustained elevated circulating levels of BNP and reduced incidence of left ventricular dysfunction over a five year follow up period. These promising data support the use of LCZ696, a first in class angiotensin receptor neprilysin inhibitor, to pharmacologically raise BNP and provide cardioprotection in at-risk patients. As neprilysin degrades biologically active NP, LCZ696 increases myocardial cyclic guanosine monophosphate (cGMP) while reducing myocardial stiffness and hypertrophy. NPs also stimulate natriuresis, diuresis, vasodilation and have been shown to have anti-fibrotic and anti-sympathetic benefits which could augment the STOP-HF preventative strategy [1, 3-5]. New data from our unit show that atrial tissue gene expression of BNP is strongly associated with atrial fibrosis, procollagen expression and presence of M2 monocyte-derived-macrophage marker CD163 [6]. Data from the STOP-HF follow-up study show that BNP strongly associates with the presence of atrial cardiomyopathy, which in turn is independently predictive of new onset major adverse cardiovascular events. Taken together, these data support the study hypothesis and a role for sacubitril/valsartan versus valsartan alone in favourably modulating progression of atrial cardiomyopathy, including left atrial volume index (LAVI), electrophysiological measures and fibrosis [6]. If left atrial structure and function is also associated with new onset major adverse cardiovascular events, the intervention could modulate propensity for thrombosis, which will be assessed using biomarkers. Finally, new CMRI imaging measures of atrial and ventricular structure and function, such as CMR e' will allow full characterisation of the cardiovascular impact of the intervention.

9.3 Rational for the study

Elevated NP in an at-risk population independently identifies cardiovascular risk, which can be specifically targeted by LCZ696. In a small number of patients (4%) with cardiovascular risk factors and elevated NP, significant asymptomatic LV systolic dysfunction will be present and for these RAAS modifying therapy is mandated.

However, there is a larger group of patients with elevated NP who have normal systolic function of the left ventricle but who have significant isolated diastolic dysfunction. These patients have asymptomatic left ventricular diastolic dysfunction (ALVDD), atrial cardiomyopathy (AC) or both and are at heightened risk for heart failure and other cardiovascular events[6].

The increase in NP in ALVDD is likely a fibro-inflammatory signal, which in-turn is contributing to tissue remodelling, vascular disease, myocardial stiffening and left ventricular dysfunction. For example, hypertension, a common risk factor for ALVDD, is associated with an adverse accumulation of fibrous tissue[6-8] and studies have demonstrated a strong relationship between ventricular stiffness, myocardial collagen content and plasma levels of myocardial collagen turnover markers[7, 8].

Moreover, there is a larger group of patients with elevated NP who have normal systolic function of the left ventricle but who have significant isolated diastolic dysfunction. These are at heightened risk for heart failure and other cardiovascular events. We, and others, have shown that cardiac inflammation, fibrosis and hypertrophy drive the pathophysiology [6,9]. Importantly, there is currently no specific therapy for these patients, beyond conventional risk factor control.

Since the increase in NP in this cohort reflects haemodynamic abnormalities and a fibro-inflammatory signal, which in turn is contributing to inflammation, tissue remodelling, vascular disease, myocardial stiffening, hypertrophy as well as left atrial and ventricular dysfunction,[3] interrupting this pathophysiological process at an early stage before the development of ventricular dysfunction, may prevent/slow development to heart failure and also have an impact on the development of other cardiovascular events driven by this pathophysiological process. Accordingly, suppressing the RAAS will reduce the pro-fibrotic impact of angiotensin II. Addition of LCZ696, which reduces degradation of endogenous, cardio-protective NPs, will augment the beneficial anti-inflammatory and anti-fibrotic effects of NPs beyond conventional RAAS modifying therapy. This may be mediated through impacts on the innate immune system and monocyte-derived-macrophage phenotypes in the myocardium. Therefore, we hypothesise that the ARNI compound, LCZ696, will demonstrate superior cardio-protection in this at-risk patient cohort and provide a novel way to modify cardiovascular risks at an earlier stage in the natural course of disease, in a targeted and individualized manner.

Collagen is a stable protein and its balanced turnover is estimated to be in the order of 80 to120 days. Alteration of collagen turnover balance by various mechanisms can lead to adverse accumulation of collagen in the myocardial interstitium leading to fibrosis, increased tissue stiffness and reduced myocardial compliance. As a result, diastolic function can become impaired and left ventricular atrial volume increases. Several studies have shown that the interplay between the myocardium and extracellular matrix (ECM) can now be evaluated via analysis of serum samples of markers of collagen turnover.[8, 9] The enzymatic steps involved in ECM metabolism present therapeutic opportunities to modulate myocardial remodelling in order to prevent the progression from ALVDD to heart failure, elevated serum levels of CITP, matrix metalloproteinase (MMP)-2 and MMP-9 suggest increased degradation of myocardial collagen and other components of the ECM. Associated elevated levels of PICP, PIIINP and a trend towards an increase in PINP levels also indicate increased collagen synthesis in diastolic heart failure[10] [11].

Other biomarkers of cardiac structure and function of relevance in AC/ALVDD include cardiac Troponin, a biomarker of cardiac damage, Galectin3 and ST-2, biomarkers of cardiac remodelling and tissue fibrosis. Finally, cGMP, which blunts activation pathways, diminishes hypertrophy, fibrosis, cellular toxicity, and maladaptive remodelling in the myocardium.

Importantly, there is currently no specific therapy for AC/ALVDD, beyond conventional cardiovascular risk factor control. Interrupting this pathophysiological process at an early stage before the development of ventricular dysfunction, may prevent/slow development to heart failure and also have an impact on the development of other cardiovascular events driven by this pathophysiological process. This represents a novel way to modify risks at an earlier stage in the natural course of cardiovascular disease, in a targeted and individualized manner. It is known that suppressing the RAAS will reduce the pro-fibrotic impact of angiotensin II. Addition of neprilysin inhibitor sacubitril in LCZ696, will reduce degradation of endogenous, cardio-protective, biologically active NPs and cGMP and will augment the beneficial impact on fibro-inflammation beyond conventional RAAS modifying therapy.

There are a number of emerging biochemical and imaging surrogates of left ventricular dysfunction, which can be evaluated in a relatively small population over a short time frame in a prospective, phase II study design. Of these, one of the most reliable, continuous markers of diastolic function in the heart is LAVI. There was a dramatic impact of LCZ696 on LAVI in comparison with valsartan in the PARAMOUNT study [12]. The reduction of 2.6 mL/m^2 with LCZ696 compared with an increase of 0.3 mL/m^2 with valsartan (p=0.007 for difference) from a baseline of 36 mL/m^2 over 36 weeks is clinically meaningful. Furthermore, this endpoint can be precisely defined using cardiac magnetic resonance imaging (cMRI) in a phase II design. We will collect Doppler Echocardiographic images to correlate with cMRI images. Doppler Echocardiography will be more feasible in follow on studies with larger populations.

In summary, it has been demonstrated that asymptomatic patients with elevated NP levels are at higher risk of heart failure and other cardiovascular events. At-risk patients with C allele of genetic variant rs198389 of the *Nppb* gene have sustained elevated BNP. Elevated NP in this setting represents a protective, endogenous response to fibro-inflammation and is associated with beneficial effects on progression of left ventricular dysfunction. The predominant cardiac abnormality in these patients is ALVDD, which can be tracked using LAVI. We hypothesise that augmenting this protective fibro-inflammatory response pharmacologically with LCZ696 could demonstrate superiority over the current state of art therapy in patients with hypertension or diabetes and elevated NP in a phase II study design and, in doing so, PARABLE may deliver a major clinical innovation in the prevention of cardiovascular disease.[3]

10. OBJECTIVES AND ENDPOINTS

The overall purpose of the PARABLE trial is to assess the impact of LCZ696 on functional, structural and biochemical abnormalities as surrogates of cardiovascular disease in an asymptomatic cohort with cardiovascular risk factors and elevated NP (brain type natriuretic peptide (BNP) and N-terminal of the prohormone BNP (NT-proBNP).

10.1 Objectives

Primary objective:

1. To assess the impact of LCZ696 (sacubitril/valsartan) versus angiotensin II receptor blocker (ARB) (valsartan) on left ventricular diastolic function over 18 months.

Secondary objectives:

1. To assess the impact of LCZ696 versus valsartan therapy on atrial and ventricular structure and function over 9 and 18 months.
2. To assess the impact of LCZ696 versus valsartan therapy on cardiovascular and non-cardiovascular adverse events, including prior to and post onset of Covid19 pandemic.

Exploratory objectives:

1. To assess response to LCZ696 based on genetic variants associated with the expression and metabolism of BNP (e.g. neprilysin, Nppb, Npra and Nprc genes).
2. To assess the impact of LCZ696 versus valsartan on biochemical markers of fibro-inflammation and myocardial damage at 9 and 18 months.
3. To assess the impact of LCZ696 versus valsartan on biochemical markers of renal function
4. To correlate the impact on left ventricular dysfunction and biochemical markers in order to understand the mechanism of benefit of LCZ696 versus ARB therapy in modulating fibro-inflammation, myocardial damage, and cardiac function.
5. To provide further information in the target subject population on the safety and tolerability of LCZ696 versus valsartan.
6. To assess the impact of LCZ696 versus valsartan on Health-Related Quality of Life and cognitive function (through the administration of questionnaires) in the target subject population.
7. To assess the difference between groups in terms of atrial and ventricular arrthymia measured non-invasively over 5-7 days using Holter monitoring
8. To assess response of immune cells to sacubitril/valsartan
9. To evaluate if the treatment effects observed during the 18-month PARABLE study persist nine-months after study drug discontinuation

10.2 Endpoints

Primary endpoint:

Change in left atrial volume index (LAVI) measured by cMRI over 18 months.

Secondary endpoints:

1. Left ventricular function using Doppler Echocardiography (average E/e') between baseline and 18 months
2. Left atrial volume index (Doppler Echocardiography LAV)/BSA* between baseline and 9 months
3. Left atrial function measured as total cMRI LAEF ((LAVimax-LAVimin)/LAVimax) over 18 months
4. Left atrial function measured as cMRI left atrial stroke volume index (LAVmax-LAVmin)/BSA*, or LAVimax-LAVimin over 18 months
5. Left ventricular structure (cMRI LVMi indexed to BSA*) over 18 months
6. Left ventricular function (cMRI LVEF) over 18 months
7. Measures of vascular compliance (ABPM pulse pressure) between baseline and 18 months
8. Change in log transformed NT-proBNP between baseline and 18 months
9. Time to first all cardiovascular death and major adverse cardiac events (MACE) requiring hospitalisation over 18 months. MACE includes arrythmia (including atrial fibrillation/flutter), transient ischaemic attack, stroke, valvular heart disease, myocardial infarction, peripheral or pulmonary thrombosis/embolus or heart failure
10. Primary endpoint analysed per protocol.

*BSA calculated using the DuBois formula

Exploratory endpoints:

11. Left atrial function measured as cMRI left atrial cardiac index ((LAVimax-LAVimin) x heart rate) between baseline and 18 months

12. Left atrial function as cMRI myocardial strain measured using feature tracking

13. Left ventricular structure (as cMRI LVESV, LVEDV, Fractal Analysis), all indexed to BSA* between baseline and 18 months

14. Left ventricular function, measured as cMRI left ventricular stroke volume index (LVESVi-LVEDVi) between baseline and 18 months

15. Left ventricular function, measured as cMRI left ventricular cardiac index ((LVESVi-LVEDVi) x heart rate) between baseline and 18 months

16. Left ventricular function as cMRI myocardial strain using feature tracking between baseline and 18 months

17. Left ventricular function as cMRI e'

18. Evaluation of treatment effects on cMRI measures of right atrial and structure and function (RAVI, RAEF, RVEF, SV, CI, E', E/e')

19. Left atrial function using Doppler Echocardiography (E/A ratio)

20. Left ventricular structure using Doppler Echocardiography (LVMI, LVESVI, LVEDVI)

21. Left ventricular function using Doppler Echocardiography (LVEF, stroke volume (SV), cardiac index (CI), Lateral or medial e' and lateral or medial E/e')

22. Change in log transformed BNP, NTproBNP/BNP ratio and other related peptides, e.g. ANP, urinary cGMP between baseline and 9 months and baseline and 18 months

23. Change in the following parameters of 24-hour ABPM between baseline and 9 months and baseline and 18 months: Systolic blood pressure, diastolic blood pressure and heart rate

24. Change in resting (clinic measured) systolic blood pressure, diastolic blood pressure and heart rate between baseline and 9 months and baseline and 18 months

25. Incidence of all cardiovascular death and MACE requiring hospitalisation over 18 months

26. Incidence of all cardiovascular related serious adverse events (SAEs) over 18 months

27. Incidence of all-cause death and SAEs requiring hospitalisation over 18 months

28. Incidence of all SAEs over 18 months

29. Incidence of all-cause adverse events (AEs) by system organ class over 18 months

30. Time to first event analysis using Kaplan Meier survival curves for cardiovascular death or MACE requiring hospitalisation, cardiovascular-related SAEs, death or all-cause emergency hospitalisations, all-cause SAE

31. Impact of LCZ696 on endpoints in a subset of patients with or without baseline atrial cardiomyopathy, using EHRA/HRS/APHRS/SOLAECE 2016 consensus definition; a complex of electrophysiological (e.g. atrial fibrillation, atrial flutter), structural/architectural (e.g. LAVI >34ml/m^2) or contractile (e.g. LAEF, elevated E/A ratio) on cMRI or Doppler Echocardiography, at 9 and 18 months

32. Impact of LCZ696 on prevalent and new onset atrial cardiomyopathy using EHRA/HRS/APHRS/SOLAECE 2016 consensus definition (defined as a complex of electrophysiological (e.g. atrial fibrillation, atrial flutter), structural/architectural (e.g. LAVI >34ml/m2) or contractile (e.g. LAEF, elevated E/A ratio) on cMRI or Doppler Echocardiography) at 9 and 18 months

33. Impact of LCZ696 on primary and secondary endpoints, at 9 and 18 months dependent on BNP above or below 50pg/mL, median BNP, NTproBNP and NTproBNP/BNP ratio at baseline

34. Correlation between Doppler Echocardiography and cMRI measures of the following at baseline and 18 months:
    - Left atrial structure and function (LAVi, LAEF)
    - Left ventricular structure and function (e.g. LVMi, LVSVi, LVCi, LVEF, e')
    - Correlation of cMRI-e' with left atrial and left ventricular feature tracking analysis

35. Change in the numbers of patients with progression of left ventricular dysfunction, defined using Doppler Echocardiography as: ejection fraction <50% with change of >5%; and/or lateral E/e' >13 or average E/e' >14 (with change >2); and/or lateral e'<9, with reduction >2 over 9 and 18 months

36. Pharmacogenetic response to therapy (i.e. change in primary and secondary endpoints) dependent on status of common genetic variants (or proxies in high linkage disequilibrium) associated with the expression and metabolism of natriuretic peptide, sacubitril and valsartan (e.g. CES, CYP2C9, NPRA, NPRB, NPPB, NPPA, NPRC (NPR3), MME, FURIN, CLCN, DPP4) at 9 and 18 months

37. Change in blood biomarkers of collagen turnover (e.g. Col1A1, PICP, PIIINP), ECM turnover (e.g. MMP-2, MMP-3, MMP-9, TIMP-1, TIMP-4), myocardial damage (hsTroponin), fibrosis (e.g. Galectin-3, sST-2), inflammation (e.g. IL-1R1, IL-R2, IL-2RA, IL-6RA, IL-6, TNF-αR1/R2, MCP1, CT1, GDF15 and RAAS (e.g. PRA, aldosterone), GP6, creatinine, ANP, uPAR, CD163, uPA, PON3, TR-AP, E/P-selectins, vWF, cystatin B, TF-PI between baseline and 18 months

38. Change in eGFR, creatinine, albumin creatinine ratio and urinary biomarkers of natriuretic peptide (e.g. cGMP, ANP) between baseline and 9 months and baseline and 18 months

39. Correlation between Doppler Echocardiography and cMRI measures of the following:
    - Left atrial structure and function (LAVI, LAEF, LVEF, SV, CI, E', E/e') and blood/urinary biomarkers
    - Left atrial and structure and function (LAVI, LAEF, LVEF, SV, CI, E', E/e') and pericardial and epicardial fat.
    - Right atrial and structure and function (RAVI, RAEF, RVEF, SV, CI, E', E/e') and pericardial and epicardial fat.

40. Change in cognitive function as measured by the Standardised Mini-Mental State Examination (MMSE) between baseline and 9 months and baseline and 18 months

41. Change in cognitive function as measured by the Montreal Cognitive Assessment tool (MoCA) between baseline and 9 months and baseline and 18 months

42. Change in quality of life as measured using the EuroQoL-5D-5L instrument (overall summary score and individual domain score) between baseline and 9 months and baseline and 18 months

43. Difference between groups in terms of CD14+, CD16+/- monocyte RNA expression profile, in a sub-set of subjects, between baseline and 9 months and baseline and 18 months

44. Impact of LCZ696 on biomarkers of angiogenesis, hypoxia, cardiac remodelling, cell adhesion, platelet activation, coagulation, chemotaxis, catabolic stress, inflammation and fibrosis which will be assessed using Olink CVIII proseek panel, between baseline and 18 months

45. Difference between treatment groups in incidence of atrial fibrillation/flutter, paroxysmal supraventricular tachycardia and other atrial or ventricular arrthymias measured non-invasively in a sub-set of subjects over 5-7 days using continuous ECG monitoring

46. Pharmacogenetic responses to therapy dependent on polygenic risk score of blood pressure, atrial fibrillation, atrial dilatation associated gene variants and established pharmacogenetic gene variants of natriuretic peptide pathway and sacubitril (e.g. NPPB, NPPA, NPRC (NPR3), MME, FURIN, CLCN, DPP4, KLKB1, CES1 gene variants) at 18 months

47. Subgroup analyses on primary outcome and secondary endpoints (pre-specified subgroups include above and below median age and BMI, eGFR (above and below 90mls/min); gender, obesity, vascular disease, hypertension, diabetes, atrial fibrillation/flutter, valve disease)

48. Use of circulating metabolomics profile to identify responders to LCZ696 (based on primary and secondary endpoints)

49. Evaluation of secondary and exploratory endpoints in a sub-set of subjects, 9 months after completing therapy with IMP (extension analysis/9 months follow-up sub-study)

50. Evaluation of primary and secondary endpoints in total follow up period and prior to Covid19 restrictions implemented (13 March 2020)

51. Additional novel and established biomarkers outlined above measured in serum, plasma, or urine profiling using proteomic, transcriptomic, epigenomic, and metabolomic approaches

52. Evaluation of health economic analysis using cost-benefit analysis and cost-benefit analysis.

11. TRIAL DESIGN

11.1 General considerations

PARABLE is a proof of concept, randomised, double-blind, double-dummy, phase II, parallel group, controlled trial. It will enrol 250 patients with cardiovascular risk factors, elevated NP and LAVI above 28 mL/m^2.

Standard dosing regimen: Following selection, discontinuation of existing angiotensin converting enzyme (ACE) inhibitor and a washout period of 36 hours, or angiotensin receptor blocker (ARB) (no washout necessary), PARABLE will randomly assign subjects to LCZ696 100mg twice daily titrated to LCZ696 200mg twice daily or valsartan 80mg twice daily titrated to valsartan 160 mg twice daily for 18 months (see Figure 1a).

Lower dosing regimen: A starting dose of LCZ696 50 mg twice daily or valsartan 40mg twice daily will be used for subjects not currently taking an ACE inhibitor or an ARB, and for subjects previously taking low doses of these agents (as per Investigator judgement). A lower dosing regimen should also be used for subjects with a systolic BP of ≥100mm to 110mmHg at screening or baseline. The dose of LCZ696 and valsartan will be doubled every 2 to four weeks to the target dose of 200mg and 160mg twice daily for LCZ696 and valsartan respectively, as tolerated by the subject. (See Figure 1b)

(LCZ696 200mg twice daily delivers similar exposure of valsartan (assessed by AUC) as Diovan 160mg twice daily. This dose delivers approximately 90% of its maximal neprilysin inhibition (Novartis Company Information)).

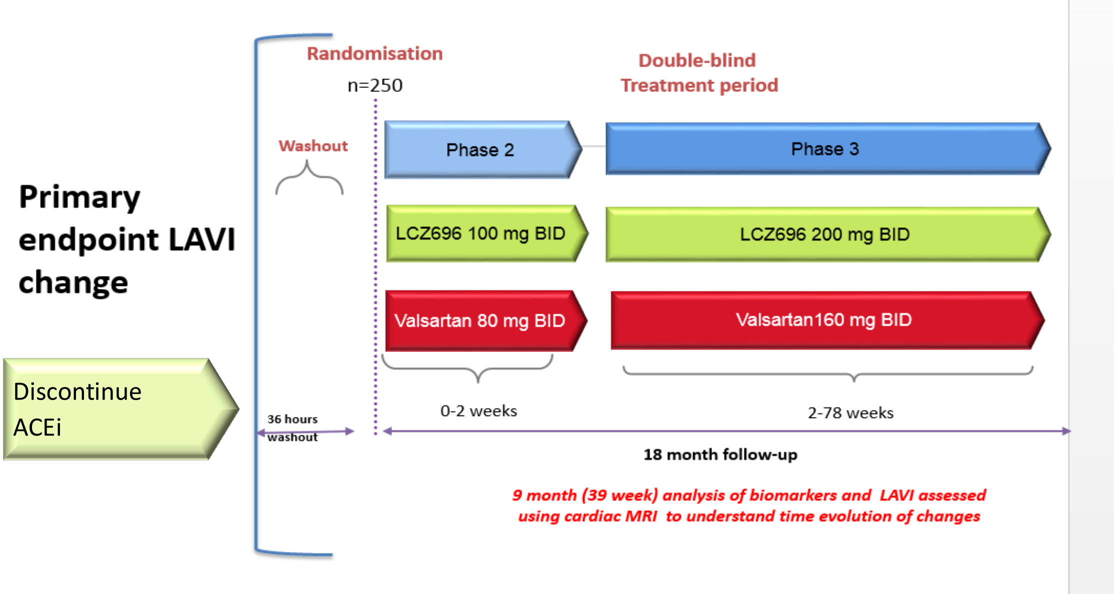

Figure 1a. Study design for subjects titrated with the standard starting dose of study drug

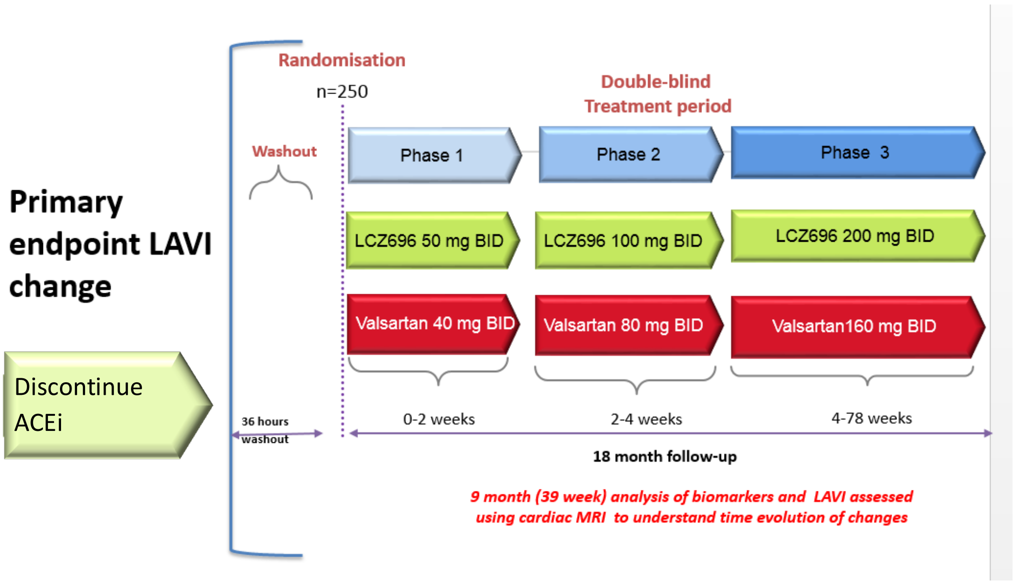

Figure 1b. Study design for subjects titrated with the lower starting dose of study drug

11.2 Selection of study population

Eligible patients will be male or female, over 40 years old, with cardiovascular risk factors, elevated NP and LAVI above 28 mL/m^2.

11.2.1 Inclusion criteria

To be eligible for inclusion, each patient must meet each of the following criteria at screening (Visit 1) and must continue to fulfil these criteria at baseline (Visit 2).

1. Age > 40yrs with cardiovascular risk factor(s) including at least one of:

    a. History of hypertension (medicated for greater than one month);
    b. History of diabetes;

2. Elevated NP: Elevated NP: BNP between 20 and 280pg/ml or NT-proBNP values between 100 pg/ml and 1,000 pg/ml within 6 months prior to screening or at screening

3. LAVI > 28 mL/m^2 obtained during Doppler Echocardiography within 6 months prior to screening or at screening

4. Subjects must give written informed consent to participate in the study and before any study related assessments are performed.

11.2.2 Exclusion criteria

1. A history of heart failure.
2. Asymptomatic left ventricular systolic dysfunction defined as LVEF <50% on most recent measurement.
3. Systolic blood pressure <100mmHg
4. Persistent atrial fibrillation.
5. History of hypersensitivity, allergy or intolerance to LCZ696, ARB or neprilysin therapy or to any of the excipients or other contraindication to their use.
6. Previous history of intolerance to recommended target doses for ARBs
7. Subjects who require treatment with both an ACE inhibitor and an ARB
8. Presence of haemodynamically significant mitral and /or aortic valve disease.
9. Presence of hemodynamically significant obstructive lesions of left ventricular outflow tract, including aortic stenosis.
10. Conditions that are expected to compromise survival over the study period.
11. Serum potassium level > 5.2 mmol/L at screening.
12. Severe renal insufficiency (eGFR <30 mL per minute per 1.73 m^2).
13. Hepatic dysfunction (AST or ALT values exceeding 3x ULN)
14. Concomitant use of aliskiren
15. History of angioedema.
16. History or evidence of drug or alcohol abuse within the last 12 months
17. Malignancy or presence of any other disease with a life expectancy of < 2 years
18. Women who are pregnant, breast-feeding, or women of child bearing potential not using estro-progestative oral or intra-uterine contraception or implants, or women using estro-progestative oral or intra-uterine contraception or implants but who consider stopping it during the planned duration of the study. A postmenopausal state is defined as no menses for 12 months without an alternative medical cause. (Contraception must be continued for one week following discontinuation of study drug).
19. Concomitant participation in other intervention trials
20. Participation in any investigational drug trial within one month of visit 1.
21. Refusal to provide informed consent
22. Subjects with contraindications to MRI
    a) Brain aneurysm clip
    b) Implanted neural stimulator
    c) Implanted cardiac pacemaker or defibrillator
    d) Cochlear implant
    e) Ocular foreign body (e.g. metal shavings)
    f) Other implanted medical devices: (e.g. Swan-Ganz catheter)
    g) Insulin pump
    h) Metal shrapnel or bullet.
23. Any surgical or medical condition which might significantly alter the absorption, distribution, metabolism, or excretion of study drugs, including but not limited to any of the following:
    a) History of major gastrointestinal tract surgery including gastrectomy, gastroenterostomy, or bowel resection.
    b) Inflammatory bowel disease during the 12 months prior to Visit 1.

c) Any history of pancreatic injury, pancreatitis or evidence of impaired pancreatic function/injury as indicated by abnormal lipase or amylase.
d) Evidence of hepatic disease as determined by any one of the following: SGOT or SGPT values exceeding 3 x ULN at Visit 1, a history of hepatic encephalopathy, a history of oesophageal varices, or a history of portocaval shunt.

No additional exclusions may be applied by the Investigator in order to ensure that the study population will be representative of all eligible patients.

12. STUDY ASSESSMENTS AND PROCEDURES

Informed consent will be obtained prior to any study-related procedures being undertaken.

12.1 Screening visit (Visit 1)

Prior to Visit 1, the Investigator shall perform a 'pre-screening' exercise and review the STOP-HF database to identify subjects that may potentially meet the inclusion/exclusion criteria (e.g. age, risk factors for heart failure and other co-morbidities).

The Investigator shall contact those subjects that meet the inclusion and exclusion criteria based on the information available in the STOP-HF database and provide information to them about the clinical trial and ascertain their interest in participating in the trial. (Prior to any contact, www.rip.ie should be checked to identify deceased patients). This contact must be documented in the patients' medical notes. Patients may also contact the STOP-HF clinic directly in response to an advertisement. Patients may also be referred from other clinics, hospital departments or healthcare professionals.

If the subject expresses interest in participating in the trial, they will be invited to attend a screening visit (Visit 1). They will be given a Subject Information Leaflet and Informed Consent Form. They will be given sufficient time to consider their participation in the study and to ask any questions concerning the study (See section 22.5 for further information on the informed consent process). Informed consent must be received before any study related procedures are carried out. Only a qualified medical doctor may obtain informed consent.

Following informed consent, various clinical laboratory assessments will be carried out to assess/confirm eligibility (e.g. potassium, liver function, eGFR). The Investigator should review the subject's medical history to confirm that no other exclusion criteria related to the study or contraindications to study medication are known. For NP, any elevated value (BNP >20pg/ml or NT-proBNP >100 pg/ml) obtained in the previous 6 months is acceptable. For LAVI, any elevated value (> 28 $mL/m^{2)}$ obtained in the previous six months is acceptable.

An appointment for Visit 2 is scheduled for up to two weeks later.

Following Visit 1 and following a review of laboratory assessment to confirm eligibility, patient randomisation will take place. The patient will be assigned to one of the two treatment arms in accordance with the randomisation scheme.

Table 1. Study interventions

Treatment arm	# of subjects randomised	Type	Compound	Min dose	Max dose (target dose)	Frequency	Route
Arm 1	~125	Active	LCZ696	50 mg*	200 mg	Twice daily	Oral
Arm 2	~125	Comparator	Valsartan	40 mg*	160 mg	Twice daily	Oral

12.2 Baseline visit (Visit 2)

Eligible patients will undergo a 36-hour washout period if they were previously taking an ACE inhibitor† and be randomized to one of two arms for 18 months:

[1] Intervention arm: LCZ696 100mg twice daily, titrated after two weeks to 200mg twice daily on top of usual medical care or

[2] Control arm: ARB (valsartan) 80mg twice daily, titrated after two weeks to 160 twice daily on top of usual medical care.

†LCZ696 must not be initiated until 36 hours after taking the last dose of ACE inhibitor.

*A starting dose of LCZ696 50mg twice daily or valsartan 40mg twice daily will be used for subjects not currently taking an ACE inhibitor or an ARB, and/or for subjects previously taking low doses of these agents (as per Investigator judgement). A lower dosing regimen should also be used for subjects with a systolic BP of ≥100mm to 110mmHg at screening or baseline. The dose of LCZ696 and valsartan will be doubled every 2 to four weeks to the target dose of 200mg and 160mg twice daily for LCZ696 and valsartan respectively, as tolerated by the subject.

All subjects will be reviewed two weeks after dose titration for tolerability issues (e.g. symptomatic hypotension, hyperkalaemia, renal dysfunction). In cases of poor tolerability, temporary down-titration of study drug, co-medication or discontinuation will be considered (see Appendix 10).

Additional medications or interventions will be administered at the discretion of the attending physician. The use of an ACE inhibitor an ARB concomitantly with the study drug after randomisation is strictly prohibited.

The Investigator shall complete a 'Wallet Card' for each patient. Patients will be advised to carry the card with them at all times and to present the card to medical personnel should they require any medical care outside the study while participating in the study.

12.3 Follow up visits (Visits 3-11)

Table 2 outlines the schedule of clinical assessments, blood sampling, imaging and follow-up that will apply in PARABLE. Follow-up visits will take place in the STOP-HF Service, St Michael's Hospital, Dun Laoghaire.

A follow-up visit will also take place two weeks after each dose titration (i.e. at week two and four and six.

If any subject refuses to return for these assessments or is unable to do so, every effort should be made to contact him/her by telephone and these attempts should be recorded in the subject's paper case report form (pCRF). If one visit is postponed or brought forward, it should not result in the next visit being postponed or brought forward. The next visit, if at all possible, should adhere to the original time schedule.

Table 2. Study assessments and procedures

Visit number	1	2	3	4	5	6	7	8	9	10	11	12
**Assessment**	**Wk-2 Screening**	**D0 Baseline**	**Wk 2**	**Wk 4**	**Wk 6**	**Wk 13 / Mth 3**	**Wk 26 / Mth 6**	**Wk 39 / / Mth 9**	**Wk 52 / Mth 12**	**Wk 65 / Mth 15**	**Wk 78 / Mth 18 (EOS)**	**Mth 27 (Optional sub-study)**
			**± 3d**	**± 3d**	**± 3d**	**± 7d**	**± 7d**	**± 7d**	**± 7d**	**± 7d**	**± 7d**	**± 28**
Assessment of eligibility	x											x
Pregnancy test (WOCBP)	x	x	x	x	x	x	x	x	x	x	x	x
Informed consent	x											x
Randomisation		x										
Medical history	x											
Physical exam	x	x	x	x	x	x	x	x	x	x	x	x
Height (H) and weight (W)		H/W						W			W	H/W
Waist/hip circumference		x						x			x	x
Vital signs (BP and pulse)	x	x	x	x	x	x	x	x	x	x	x	x
Natriuretic peptide	x[#]	x	x	x	x	x	x	x	x	x	x	x
Biomarkers		x			x	x	x	x	x	x	x	x
U&E, LFT, Alk phos	x (no alk phos)	x	x	x	x	x	x	x	x	x	x	x
Haematology	x	x						x			x	x
eGFR	x	x						x			x	x
HbA1c, lipid profile, iron studies		x						x			x	x
cGMP (urinary)		x			x	x		x			x	x
ABPM		x						x			x	x
Electrocardiography (ECG)		x						x			x	x
Doppler echocardiography	X[¥]	x[†]						x			x	x
Cardiac MRI[ƶ]		x									x	
Questionnaires**		x						x			x	x
Concomitant medications	x	x	x	x	x	x	x	x	x	x	x	
Adverse events			x	x	x	x	x	x	x	x	x	x
Dispense medication		x	x	x	x	x	x	x	x	x		
Drug accountability			x	x	x	x	x	x	x	x	x	
Holter monitor (optional)											x	

*Source: results of these assessments may be filed in the Subject Study File on the original source document (questionnaires) or a copy of the source document (e.g. laboratory report, Echo report, ECG (this must be a copy of the original), ABPM printout etc.)

¥ Echo may be needed at Visit 1 if one hasn't been performed in the previous six months.

† If an Echo is performed at screening, it does not have to be repeated at baseline, unless the investigator decides otherwise. Baseline echo must be performed within 90 days prior to baseline.

** The SMMSE and MoCA cognitive assessment questionnaire will also be administered five years after the patient's end of study visit.

Ƶ If the patient had an MRI in the previous six weeks, then there is no need for a repeat MRI at baseline

NP levels may be needed at Visit 1 if one hasn't been performed in the previous six months.

12.4 Assessments

12.4.1 Laboratory parameters

Peripheral venous blood samples will be drawn at the timelines shown in Table 2. Laboratory examinations are analysed in St Michael's Hospital laboratory or St Vincent's Hospital laboratory. Methods for sampling and handling are detailed in the PARABLE laboratory manual (HBT-GCP-SOP-16 'Handling Laboratory Samples for the PARABLE Study'). Evaluations will include the following:

- Biochemistry
  - Urea & Electrolytes (U&E)
  - Liver Function Tests (LFT)
  - Alkaline Phosphatase
  - Lipid profile
  - HbA1c
  - Iron studies
- Haematology
  - Full blood count

Sample processing and analysis will be done as per local standard operating procedures. The local hospital laboratory should provide the report to the Investigator who is responsible for the clinical assessment of the results. Any abnormality should be assessed for its clinical significance. Any clinically significant value should be recorded as an AE in the CRF and followed up.

12.4.2 eGFR assessments

The eGFR to determine eligibility of the subject for screening into the trial will be calculated at Visit 1 (screening) from the serum creatinine concentration. The eGFR will be further measured at Visit 8 (month 9) and Visit 11 (EOS).

eGFR will only be calculated using the following formula: eGFR (mL/min/1.73 m^2) = 175 × (standardized SCr in mg/dL)-1.154 × (age in years)-0.203 × (0.742 if female) × (1.212 if black), where SCr is the standardized serum creatinine value.

12.4.3 Urinary biomarkers

A spot office urine sample (random sample) will be collected from subjects at Visit 2 (baseline), Visit 5 (week 6), Visit 6 (month 3), Visit 8 (month 9) and Visit 11 (EOS/18 month) for assessment of cGMP and atrial natriuretic peptide (ANP).

12.4.4 Biomarkers

Peripheral blood samples and plasma for biomarker analysis will be drawn at the timelines shown in Table 2. Samples will undergo immediate serum separation by centrifuge at 1500 rcf (relative centrifugal force) for 15 minutes at 21°C. The serum and plasma will then be separated into aliquots and stored at -80°C. They will be

transported on dry ice to the Conway Institute, University College Dublin for simultaneous analysis of selected biomarkers from the Table below using validated analytical techniques:

- Col1A1
- PICP
- PIIINP
- MMP-2
- MMP-3
- MMP-9
- TIMP-1
- TIMP-4
- GP6
- sST-2
- hsTroponin
- Galectin-3
- PRA
- uPAR
- CD163
- uPA
-
- PON3

- Aldosterone
- Creatinine
- IL-6
- IL-1R1
- IL-R2
- IL-2RA
- IL-6RA
- IL-17RA
- TNF-alpha R1/R2
- ANP
- MCP1
- CT1
- GDF15
- TR-AP
- E/P-Selectins
- vWF
- Cystatin B
- TF-P1

12.4.5 Serum BNP and NT-proBNP

NP measurements required for eligibility will be based on levels measured within the last 6 months or at screening.

Serum BNP from the peripheral circulation will be measured using the Alere Triage® BNP test, a POC fluorescence immunoassay. Several drops of EDTA anticoagulated whole blood or plasma is added to the sample port of the test device which is then inserted in to the meter. The concentration of BNP in the specimen is directly proportional to the fluorescence detected. The results are displayed on the meter screen approximately 15 minutes from the addition of the specimen.

Serum NT-proBNP from the peripheral circulation will be measured by the St Vincent’s Healthcare Group Laboratory according to standard hospital policy.

12.4.6 Medical history

The subject’s medical history will be recorded at the screening visit.

12.4.7 Demographics

The subject’s date of birth, gender and race will be recorded at the screening visit.

12.4.8 Physical exam

A short physical exam will include the examination of general appearance and a cardiac exam at every visit. Information will be recorded in the CRF. Significant findings that are present prior to the start of study drug must be included in the relevant section of the subject's CRF. Significant findings made after the start of study drug which meet the definition of an AE must be recorded on an AE form in the subject's CRF.

12.4.9 Vital signs

Vital signs will be assessed at every visit. This will include BP and pulse measurements. BP will be measured by using a standard sphygmomanometer with an appropriate size cuff and the non-dominant arm in the sitting position after 5 minutes of rest. This BP (and not 24h ABPM) will be used to confirm eligibility and inform dosing decisions.

12.4.10 Height, weight and waist/hip circumference

Height in centimetres (cm) will be measured at Visit 2 (baseline). Body weight (to the nearest 0.1 kilogram [kg] in indoor clothing without shoes) and waist/hip circumference (at natural waistline, which is located above the belly button and below the rib cage; start at one hip and wrap the tape measure around the rear, making sure the tape is over the largest part of the buttocks) (to the nearest centimetre [cm] in indoor clothing) will be measured at Visit 2 (baseline), Visit 8 (month 9) and Visit 11 (EOS).

12.4.11 Ambulatory blood pressure monitoring (ABPM)

ABPM will be measured at Visit 2 (baseline), Visit 8 (month 9) and Visit 11 (EOS).

The device employed for ABPM analysis will be the SpaceLab 90207 oscillometric device. Depending on arm circumference, two standard cuff sizes will be used with application to the non-dominant arm. Subjects will be instructed to go about their usual activities with minimal restrictions. The systolic blood pressure (BP), diastolic BP, and heart rate of each subject will be automatically measured every 30 minutes for 24 consecutive hours with a properly calibrated SpaceLab 90207 device. The device will be fitted and removed by a cardiovascular technician/nurse blinded to study treatment allocation. If quality criteria are not met, one repeat measurement will be allowed during the following 1–2 month period. Nocturnal dipping is defined as subjects with a more than 10% fall in nocturnal BP relative to daytime values. Non-dipping is defined as a less than 10% nocturnal fall in BP.

12.4.12 Pregnancy test

A dipstick urinary pregnancy test will be carried out on all women of childbearing potential at each study visit.

12.4.13 Electrocardiography (ECG)

A standard 12-lead ECG (after 5 minutes rest) will be performed (at Visit 2 (baseline), Visit 8 (month 9) and Visit 11 (EOS)) using appropriate procedures (i.e. paper speed 25 mm/sec, amplitude 10 mm/mV, at least 10 seconds recording available for measurement) and the use of an automatic recorder with the capacity for digital signal processing. The heart rate will be measured, and relevant abnormalities regarding cardiac rhythm, conduction and repolarisation parameters such as QT prolongation or atrio-ventricular block, will be recorded. A copy of the print-out will be kept in the subjects study file. The results will be assessed within the CRF as within normal limits, abnormal but not clinically significant or abnormal and clinically significant.

12.4.14 Echo-Doppler studies

Echocardiography will be performed at Visit 2 (baseline), 8 (month 9) and 11 (EOS) to assess myocardial function. This will be performed by a designated experienced echocardiographer who will be blinded as to treatment assignment.

LAVI measurements required for eligibility (LAVI >28 mL/m^2) will be based on echocardiograms performed within the last 6 months. If a LAVI measurement from the last six months is not available, an additional echocardiography may be performed at the screening visit. If an echo is performed at the screening visit, this can be used for the baseline visit (unless using clinical judgement, the investigator decides that another echo is necessary). A baseline echo must be performed within 90 days prior to baseline visit.

The following parameters will be reported:

- Left ventricular ejection fraction
- LAVI
- E/A ratio
- E wave deceleration time
- E/E'
- Isovolumetric relaxation time
- Pulmonary venous flow analysis
- Tissue Doppler studies

Standard two-dimensional, targeted M-Mode and Doppler Echocardiographic measurements will be obtained with the subject lying in the left lateral position. M-mode measurements will be taken according to the guidelines from the American Society of Echocardiography. Left ventricular ejection fraction will be calculated using the Simpson's biplane method. The following pulsed Doppler measurements from 3 consecutive cardiac cycles will be obtained in the apical view with a cursor at mitral

valve inflow: maximal early (E) and late (A) transmitral velocities in diastole and E wave deceleration time. Isovolumetric relaxation time will be measured in the apical four chamber view by continuous wave Doppler placed between the mitral inflow area and left ventricular outflow tract. Left ventricular filling pressures were noninvasively assessed through analysis of the ratio of peak E-wave (E) to maximum velocity of lateral mitral valve annulus (E') ratio (E'/E) measured at the lateral mitral valve annulus. An experienced sonographer blinded to treatment allocation will carry out all echocardiography imaging and reporting.

Tissue Doppler imaging will be used to record longitudinal velocities of the mitral anulus. The velocities will be recorded from the apical four-chamber view by placing a 2-5mm sample volume of the lateral portion of the mitral anulus. The E' velocity will be recorded and the ratio between E and E' (E/E') calculated.

In assessing changes over 18 months of left ventricular structure and function as part of the secondary objectives of the study, a E/E'> 15 is taken as evidence of diastolic dysfunction. An E/E' < 8 excludes the diagnosis. An E/E' ratio ranging from 8 to 15 is considered suggestive but non-diagnostic evidence of left ventricular diastolic dysfunction and needs other imaging evidence of diastolic dysfunction. These can consist of blood flow Doppler of mitral valve or pulmonary veins, echo measures of LV mass index or left atrial volume index, electrocardiographic evidence of atrial fibrillation.

12.4.15 Cardiac magnetic resonance imaging (cMRI)

Overview of cMRI in PARABLE

MRI imaging will be performed at baseline and 18 months of the study to assess myocardial structure and function. Measures of interest will include LAVI, left ventricular mass index, ejection fraction and aortic distensibility. (If the patient had an MRI in the previous six weeks, then there is no need for a repeat MRI at baseline).

Changes in cMRI measures of LAVI will comprise the primary endpoint of the study. Imaging will be carried out in St Vincent's Private Hospital. See 'Cardiac MRI for the PARABLE study' Work Instruction (HBT-GCP-WI-01) for details on how to refer subjects. cMRI analysis will be performed by a designated radiologist assigned to this study who will be blinded to the treatment assignment.

Image acquisition and analysis

Imaging will be performed using a Phillips 1.5T scanner or a Siemens Avanto 1.5 T scanner equipped with a phased-array coil placed over the praecordium. Patients will be scanned in the supine position, and after localizing scans, ECG-gated steady state free precession (SSFP) cine images of the heart will be acquired at end expiration in three standard long planes (vertical and horizontal long axes and 3 chamber view). Subsequently, multiple short-axis (SA) slices will be acquired from the base to the apex of the heart, with slice thickness of 8 mm and interslice gap of 2 mm, creating atrial and ventricular SA stacks.

Images will be analysed offline by an experienced operator familiar with clinical CMR & with the analysis methods described. Analysis will be performed on each scan using CMR tools (Cardiovascular Imaging Solutions, London, UK) or Argus Syngo MR software (Siemens Medical Imaging, Erlangen, Germany) as follows:

The multislice, short-axis cine datasets will be analysed to calculate LV, LA and right ventricular (RV) volumes and masses. Endo- and epicardial borders will be traced manually on end-diastolic and end-systolic frames in contiguous short-axis slices. Left ventricular end-diastolic (EDV) and end-systolic volumes will be calculated using summation of area × (slice thickness + interslice gap) for each slice (Simpson's method), which will allow calculation of LVEF and LV mass. The end systolic frame is considered to be the frame in which the LV cavity visually appears smallest. At the base of the heart, slices are considered to be within the LV at end diastole and end systole if the cavity is surrounded by 50% or more of ventricular myocardium. If the basal slice contains both ventricular and atrial myocardium, contours will be drawn up to the junction and joined by a curved line through the blood pool. Papillary muscles will be excluded from LV volume measurements and included in mass calculations. The inter-ventricular septum is considered part of the LV. Left atrial and right ventricular volumes, mass, and EF will be calculated in a similar fashion. All measurements will be indexed to body surface area.

Further analysis of the left atrium will be performed in the long axis views. Left atrial maximum volume (the frame just before mitral valve opening) and minimum volume (the frame just after mitral valve closure) will be measured in both two- and four-chamber views by manually tracing the LA endocardial border. The anterior border is at the mitral annular plane and the posterior border at the ostia of the pulmonary veins, excluding the LA appendage. Left atrial volumes will be calculated using the area–length method (volume ¼ 0.85 × area^2/length) and LA emptying function (LAEF) will be calculated using the formula: [(LA max - LA min)/LA max] × 100%. We will also calculate LA total emptying volume (reservoir function) using (LA max - LA min) and LA conduit volume using (LV stroke volume – LA total emptying volume).

The imaging process will be synchronised with the R wave of the ECG. Images will be acquired during each heartbeat in mid-diastole during breath hold. A non-selective inversion prepulse will be used in order to accentuate the difference between T1 images in normal and abnormal myocardium. Segmented T1 weighted fast gradient echo sequence images will be conducted after inversion delay time. LV long axis and contiguous short axis slices is acquired using an inversion delay time of 200-380ms. Images will be analysed for the presence of abnormal enhancement by an experienced operator. A Radiology Report will be provided to the Investigator for filing in the Subject Study File.

12.4.16 Concomitant medications. Potential drug interactions with LCZ696 and/or valsartan therapy

At every visit the Investigator should question the study subject on any changes in concomitant medications (new medications or changes in dose of existing

medications). The following is a list of medications that have the potential to interact with LCZ696 and/or valsartan:

- *ACE Inhibitors.* Due to the risk of angioedema, LCZ696 or valsartan should not be used concomitantly with an ACE inhibitor. LCZ696 must not be initiated until 36 hours after taking the last dose of ACE inhibitor therapy. If LCZ696 is stopped, ACE inhibitor therapy must not be initiated until 36 hours after the last dose of LCZ696.
- *Angiotensin receptor blocker:* LCZ696 should be not co-administered with an ARB due to the angiotensin II receptor blocking activity of LCZ696.
- *Aliskiren:* LCZ696 or valsartan must not be administered with aliskiren
- *Lithium:* Reversible increases in serum lithium concentrations and toxicity have been reported during concurrent use of ACE inhibitors. Due to the lack of experience with concomitant use of valsartan and LCZ696 and lithium, this combination is not recommended. If the combination proves necessary, careful monitoring of serum lithium levels is recommended
- *Antihypertensive agents:* LCZ696 and valsartan may increase the effects of other agents with antihypertensive properties (e.g. guanethidine, methyldopa, vasodilators, ACE inhibitors, beta-blockers, calcium channel blockers, diuretics and direct renin inhibitors).
- *Pressor amines (e.g. noradrenaline, adrenaline).* Possible decreased response to pressor amines.
- *Non-steroidal anti-inflammatory drugs (NSAIDs):* In elderly subjects, volume depleted subjects (including those on diuretic therapy), or subjects with compromised renal function, concomitant use of LCZ696/valsartan and NSAIDs including selective cyclooxygenase 2 (COX-2) inhibitors, may lead to an increased risk of worsening of renal function. Concomitant use of valsartan and NSAIDs may also lead to an increase in serum potassium. Therefore, monitoring of renal function is recommended when initiating or modifying treatment in subjects in LCZ696 or valsartan who are taking NSAID concomitantly. Co-administration of valsartan and NSAIDs may attenuate the antihypertensive effect of valsartan.
- *Medications known to raise potassium levels:* Potassium-sparing diuretics (e.g., spironolactone, triamterene, amiloride), potassium supplements, aldosterone antagonists, salt substitutes containing potassium and other substances that may increase potassium levels (heparin etc.) should be used with caution due to the increased risk of hyperkalaemia. If a medicinal product that affects potassium levels is considered necessary, monitoring of potassium plasma levels is advised.
- *Bile acid sequestering agents (e.g. cholestyramine and colestipol):* use is prohibited to avoid interference with study drug absorption

- *Phosphodiesterase inhibitors:* Due to more than additive BP reduction, caution is recommended when co-administering LCZ696 with sildenafil or other PDE-5 inhibitors (tadalafil, vardenafil etc).
- *Statins:* Concomitant administration of LCZ696 and atorvastatin may increase the plasma concentration of atorvastatin due to the OATP1B1 and OATP1B3 inhibitory properties of sacubitril (following concomitant administration of LCZ696 and atorvastatin, the AUC of atorvastatin and its active metabolites, o-hydroxyatorvastatin and p-hydroxyatorvastatin was increased by up to 34%). Caution is recommended when co-administering LCZ696 with atorvastatin or other statins that are substrates of OATP1B1 and OATP1B3, such as simvastatin, pravastatin and/or pitavastatin. Monitor the patient carefully for adverse effects of statin therapy (e.g. muscular side-effects (e.g. myopathy, myositis, myalgia), GI disturbances, altered LFTs, sleep disturbance, headache, dizziness).
- *Furosemide:* LCZ696 may reduce the plasma levels of furosemide which may result in a reduced diuretic response. Monitor patient carefully.
- *Nitrates:* Co-administration of LCZ696 with nitroglycerine may be associated with an increased reduction in heart rate compared to the administration of nitroglycerine alone. In general, no dose adjustment is needed.
- *Metformin*: LCZ696 may reduce the plasma concentrations of metformin. The clinical relevance of this finding is unknown. Therefore, when initiating therapy with LCZ696 in patients receiving metformin, the clinical status of the patients sould be evaluated.
- *Other:* Co-administration of rifampicin, ciclosporin, tenofovir, cidofovir or ritonavir may increase the systemic exposure of these drugs to valsartan. Exercise appropriate care when initiating or ending concomitant treatment with such drugs.

12.4.17 Questionnaires

1. *EuroQOL*-5D (EQ-5D): EQ-5D is an instrument used to assess the current health status of subjects. It consists of five domains and one visual analogue scale. This instrument assesses morbidity, self-care, usual activity, pain and anxiety and depression of subjects. It is administered in less than 5 minutes (See Appendix 3).
2. *Standardised Mini-Mental State Examination (SMMSE):* The purpose of the SMMSE-2 is to screen for cognitive impairment. It is a 30 point test, administered in approximately 10 minutes, and measures various domains of cognitive function including orientation to time and place; registration; concentration; short-term recall; naming familiar items; repeating a common expression; and the ability to read and follow written instructions, write a sentence, construct a diagram, and follow a three-step verbal command.(See Appendix 2).
3. *Montreal Cognitive Assessment (MoCA) (Version 7.3):* MoCA is a 30 point test, administered in approximately 10 minutes to assess several cognitive domains including short-term memory recall, visuospatial abilities, executive functions, attention, concentration and working memory, language, orientation and place (See Appendix 1). The administration and scoring instructions are outlined in appendix 4).

All three questionnaires will be completed at Visit 2 (baseline), Visit 8 (month 9) and Visit 11 (EOS). The SMMSE and MoCA questionnaires shall also be administered five years after the end of study visit for each patient. A record shall be made in the clinic database to ensure follow-up of this requirement. Any illnesses identified following the administration of a questionnaire should be managed appropriately. Questionnaires are available in English only. Subjects who are not capable of completing the questionnaire in English are exempt from completing the questionnaires.

13. STUDY DRUG

LCZ696, valsartan and matching placebos are provided by Novartis Ireland Ltd to the distributor company, Almac. HBT-GCP-SOP-17 ‘Study Drug Management for the PARABLE Study’ outlines the procedure for the allocation/dispensing of the study drug.

13.1 Blinding

LCZ696, valsartan and matching placebos are provided by Novartis (via the distributor company, Almac). All tablets (LCZ696 50mg, 100mg and 200mg) and valsartan (40mg, 80mg and 160mg) have different shapes and colours. Therefore, this study is a double-blind, double dummy to ensure the blinding of patients and investigators during the entire course of the study. To maintain the blinding, subjects will be required to take their assigned active treatment tablet along with placebo matching the opposite

treatment twice daily (morning and evening) in addition to their conventional concomitant therapy (except for ACE inhibitor or ARB, which will be substituted with study drug).

The following are provided by Novartis (via Almac):

- **Phase 1**
  - LCZ696 50mg film-coated tablets (LCZ696 Phase 1)
  - Placebo to match LCZ696 50mg film-coated tablets (placebo matching LCZ696 Phase1)
  - Valsartan 40mg film-coated tablets (Valsartan Phase 1)
  - Placebo to match Valsartan 40mg film-coated tablets (placebo matching LCZ696 Phase 1)

- **Phase 2**
  - LCZ696 100mg film-coated tablets (LCZ696 Phase 2)
  - Placebo to match LCZ696 100mg film-coated tablets (placebo matching LCZ696 Phase 2)
  - Valsartan 80mg film-coated tablets (Valsartan Phase 2)
  - Placebo to match Valsartan 80mg film-coated tablets (placebo matching LCZ696 Phase 2)

- **Phase 3**
  - LCZ696 200mg film-coated tablets (LCZ696 Phase 3)
  - Placebo to match LCZ696 200mg film-coated tablets (placebo matching LCZ696 Phase 3)
  - Valsartan 160mg film-coated tablets (Valsartan Phase 3)
  - Placebo to match Valsartan 160mg film-coated tablets (placebo matching LCZ696 Phase 3)

13.2 Study drug supply and storage

The study drug and matching placebo are supplied by Novartis using Almac as the distributor. The investigators will monitor the stock of study drug and notify the sponsor when stocks are running low. It is the responsibility of the sponsor to re-order supplies from Almac when required. Study drug will be delivered directly to the investigator site (The STOP-HF Service) at a pre-arranged time.

The study drug will be stored in a secure, temperature monitored cabinet in the STOP-HF Service. It is the responsibility of the investigator staff to ensure the study drug is stored securely and correctly. Investigational products are for investigational use only are to be used only within the context of this study.

13.3 Accountability of the study drug

The investigator site will maintain an accurate record of the shipment and dispensing of each study drug in a drug accountability log (HBT-GCP-FM-22; 'Study Drug Inventory and Subject Assignment Log'). Monitoring of drug accountability will be performed by the monitor during site visits and at the completion of the trial. Subjects will be asked to return any unused study drug and packaging at each visit, at the time of study drug discontinuation and at the end of the study. All patient returns should be recorded in the CRF and in the Study Drug Returns Log (HBT-GCP-FM-23)

At the conclusion of the study, and as appropriate during the course of the study, the Investigator will return all unused study drug, including opened and unopened packaging, and a copy of the completed Study Drug Returns Log to the sponsor according to the instructions provided by them.

13.4 Dispensing the study drug

All study drug is provided to the investigator site labelled. Medication labels are in English and comply with the legal requirements for clinical trials in Ireland.

At each visit, study subjects will be provided with two medication 'kits'; one containing the study drug (LCZ696 or valsartan) and one containing placebo drug corresponding to their assigned treatment arms and dose level, sufficient to last until the next scheduled visit. Records of each dispensing shall be maintained using a drug accountability log (HBT-GCP-FM-22; 'Study Drug Inventory and Subject Assignment Log').

Table 2 and 3 below summarises the study drug that will be dispensed at the various clinic visits for subject on the standard dosing schedule and the lower dosing schedule respectively. (A lower starting dose of LCZ696 50mg twice daily or valsartan 40mg twice daily will be used for subjects not currently taking an ACE inhibitor or ARB, for subjects previously taking low doses of these agents (as per Investigator judgement) and for subjects with a systolic BP of ≥100mm to 110mmHg at screening or baseline).

The procedure for the dispensing/allocation of medication pack is described in HBT-GCP-SOP-17 'Study Drug Management for the PARABLE Study'.

Subjects will be required to take the study drug and matching placebo twice a day (morning and evening) for the duration of the study. Patients will be instructed to take their morning dose at approximately 08.00 and their evening dose at approximately 19.00. The medications should be taken with a glass of water with or without food. If the subject misses a dose, he/she should take it as soon as possible, unless if it is almost time for the following scheduled dose. In this case, the subject should skip the missed dose and return back to his/her regular study drug administration schedule. The patient should be instructed to contact the Investigator if he/she is unable to take the study drug as proscribed for any reason.

Table 2. Study drug dispensed (standard dosing schedule)

Visit #	Dose level	LCZ696	Valsartan
1 (screening)	-	-	-
2 (Day 0 /baseline)	2	• LCZ696 100mg tablets<br>• Placebo to match Valsartan 80mg tablets	• Valsartan 80mg tablets<br>• Placebo to match LCZ696 100mg tablets
3-11 (Day 14-EOS)	3	• LCZ696 200mg tablets<br>• Placebo to match Valsartan 160mg tablets	• Valsartan 160mg tablets<br>• Placebo to match LCZ696 200mg tablets

Table 3. Study drug dispensed (lower dosing schedule)

Visit #	Dose level	LCZ696	Valsartan
1 (screening)	-	-	-
2 (Day 0 / baseline)	1	• LCZ696 50mg tablets<br>• Placebo to match Valsartan 40mg tablets	• Valsartan 40mg tablets<br>• Placebo to match LCZ696 50mg tablets
3 (Day 14)	2	• LCZ696 100mg tablets<br>• Placebo to match Valsartan 80mg tablets	• Valsartan 80mg tablets<br>• Placebo to match LCZ696 100mg tablets
4-11 (Day 28-EOS)	3	• LCZ696 200mg tablets<br>• Placebo to match Valsartan 160mg tablets	• Valsartan 160mg tablets<br>• Placebo to match LCZ696 200mg tablets

13.5 Assessment of compliance

Compliance will be assessed by the Investigator at each visit using pill counts and information provided subject and recorded in the CRF. The Investigator will counsel the subject if compliance is below 80%.

13.6 Overdose of study treatment

Any accidental overdose with the study drug by a study subject or a person in his/her family must be notified to the Sponsor, whether or not it has medical consequences (serious or non-serious AE, or absence of signs and symptoms).

13.7 Permitted study drug dose adjustment and interruptions

Every attempt should be made to maintain subjects on the target dose level (i.e. LCZ696 200mg and Valsartan 160mg) for as long a duration as possible throughout the trial. If, however, in the opinion of the Investigator, the subject does not tolerate the target dose of study drug (Phase 3), the Investigator should consider whether non-disease-modifying medication (e.g., calcium channel blockers, diuretics, nitrates, alpha blockers) can be reduced, before considering reducing the dose of the study drug to the next lower dose phase.

If down titration is necessary, the subject should be down-titrated to the next lower dose phase. The subject may continue receiving the lower dose phase for a recommended period of 1 to 4 weeks before re-challenging the subject with the next higher dose phase. For example, a subject who encounters tolerability problems at the target dose level (dose phase 3), should receive the study drug at dose phase 2 for 1 to 4 weeks. Then, he/she should be re-challenged with up-titration back to dose phase 3. If the tolerability issues are not alleviated despite down-titration by one dose phase, the Investigator may lower the study drug dose further to the next lower phase for 1 to 4 weeks, up to temporary withdrawal of the study drug. Again, once stable, the subject should be rechallenged with up-titration to the next higher dose phase every 1 to 4 weeks in an attempt to bring back the subject gradually to the target study drug dose level (dose phase 3). The Investigator may choose the next dose phase for down- or up-titration according to his or her judgment. Dose phase 1 or 2 could be maintained if the Investigator considers that the subjects condition would not allow any further up-titration to the target dose of study medication (phase 3). In this case it would be acceptable to maintain the subject at dose phase 1 or phase 2, whichever is the higher and tolerated dose phase by the subject.

If needed, the study drug may be stopped completely, but the subject should continue to attend the study visits and be followed until the completion of the study. Ultimately the goal is to keep the subject on the highest study drug dose possible for as long as possible and to follow the subject in the study as long as possible. Study drug dose level adjustments should mainly be based on overall safety and tolerability with special focus on a) hyperkalaemia; b) symptomatic hypotension; and c) clinically significant decrease in eGFR/increase in serum creatinine (see Appendices 7-10 for treatment guidelines for hyperkalaemia, management of BP, and renal dysfunction). All dosage adjustments should be recorded in the subjects CRF.

13.8 Study drug restart after temporary treatment interruption

Study drug should be reintroduced in those who temporarily discontinue it as soon as medically justified in the opinion of the Investigator and at the most appropriate and allowable dose level per his/her medical judgment. Should the subject not tolerate the re-start study drug dose level, he/she may be down-titrated again (if appropriate) or discontinue the study medication again and a new attempt to up-titrate or reintroduce the study drug could be considered by the Investigator as soon as medically justified in his/her medical judgment.

13.9 Study drug discontinuation and premature withdrawal of subjects

After randomisation, study drug discontinuation for any reason does not constitute withdrawal from the study and should not lead to the subject being withdrawn from the entire study. On the contrary, even subjects who have stopped taking study drug are expected to attend all the protocol specified study visits and perform all assessments as stipulated in the visit schedule (Table 2). If the subject does not attend the study visits, follow-up should continue according to the specified schedule by telephone to determine if any of the health events/endpoint prespecified in the protocol has occurred, except in the case that the subject specifically refuses such follow-up and withdraws his/her consent. The emergence of the following circumstances will require study drug discontinuation:

- Subject wishes to withdraw from study with or without stated reason
- Pregnancy
- Investigator thinks that continuation would be detrimental to the subject's well-being
- Suspected occurrence of angioedema

Study medication may be discontinued at the Investigator's discretion if any of the following occurs:

- Any subject who experiences an AE may be withdrawn at any time from the study at the discretion of the Investigator. However, the following AE warrants immediate withdrawal;
    - Angioedema: if angioedema (including swelling of the larynx and glottis, causing airway obstruction and/or swelling of the face, lips, pharynx, and/or tongue) occurs, study drug should be immediately discontinued and appropriate therapy and monitoring should be provided until complete and sustained resolution of signs and symptoms has occurred. Study drug must not be re-administered.
    - Serious hypersensitivity reactions
- Depending on the serum potassium, blood pressure, or eGFR, subjects may need to have their study drug dose or the dose of another concomitant medication reduced or discontinued, or, if appropriate, have potentially contributing agents adjusted. Please refer to Appendices 7-10 for treatment guidelines for hyperkalaemia, hypotension, or renal dysfunction.

In the case of study drug discontinuation, the subject should continue to complete all scheduled study visits and procedures. If the subject refuses, he/she should be contacted by telephone in place of protocol-specified visits unless the subject expressly refuses such contacts. A record of all attempts to contact the subject should be recorded in the Telephone Contact Log in the CRF.

If a subject is withdrawn from the study due to an AE, the AE should be noted on the appropriate CRF and the subject's progress should be followed until the AE is resolved. This follow-up may occur by phone.

13.10 Emergency unblinding of treatment assignment

Emergency unblinding should only be undertaken if knowledge of the subject's trial treatment affects the immediate, urgent medical treatment of the subject. Most often, study drug discontinuation and knowledge of the possible treatment assignments are sufficient to treat a study subject who presents with an emergency condition.

If the treatment allocation needs to be revealed, contact the WebEz Helpdesk as per the process outlined in the SOP ‘Unblinding of the Study Drug during PARABLE by the Investigator’ (HBT-GCP-SOP-12)

Study drug must be discontinued after emergency unblinding. Study drug also must be discontinued for any subject whose treatment code has been broken inadvertently or for any non-emergency reason and the investigator becomes aware of the treatment allocation.

13.11 Definition of end-of-trial

The end-of-trial is the date of the last visit of the last subject enrolled in the 9-month follow-up sub-study.

Other end of study considerations

The Sponsor or Principle Investigator has the right at any time to terminate the study for clinical or administrative reasons. In terminating the study, the Sponsor and the Principle Investigator will ensure that adequate consideration is given to the protection of the best interests of the participants.

The end of the study will be reported to the Ethics Committee (EC) and Regulatory Authority within 90 days, or 15 days if the study is terminated prematurely. The Investigators will inform subjects and ensure that the appropriate follow-up is arranged for all involved.

A summary report of the study will be provided to the sponsor (Novartis), the EC and HPRA within 12 months of the end of the study. This is a legal requirement. The final study report shall include a full summary of safety and efficacy information from the study and shall comply with the established principles and standards for the corresponding format according to ICH-GCP.

14. SAFETY REPORTING

The safety and tolerability of the study drugs will be evaluated starting from the administration of the study drug (Visit 2) through to EOS visit. Information regarding all AEs will be collected at each study visit based on information provided spontaneously by the subject and/or through questioning of the subject.

14.1 Definitions

Adverse event (AE)

Any untoward medical occurrence in a patient or clinical trial subject administered a medicinal product and which does not necessarily have a causal relationship with this treatment. An AE can therefore be any unfavourable and unintended sign (including an abnormal laboratory finding, for example), symptom or disease temporally associated with the use of a medicinal product, whether or not considered related to the medicinal product.

Worsening of a pre-existing medical condition (e.g., cancer, diabetes, migraine headaches, gout) should be considered and AE if there is either an increase in severity, frequency, or duration of the condition or an association with significantly worse outcomes. Abnormal signs and symptoms identified during the physical and cardiac exam may also constitute an adverse event if they were not present at baseline. Interventions for pre-treatment conditions (e.g., elective cosmetic surgery) or medical procedures that were planned before study enrolment are not considered AEs.

The Investigator is responsible for reviewing laboratory test results and determining whether an abnormal value in an individual study subject represents a change from values before the study. In general, abnormal laboratory findings without clinical significance (based on the Investigator's judgment) should not be recorded as AEs; however, laboratory value changes requiring therapy or adjustment in prior therapy are considered AEs.

All AEs are recorded in the CRF and on the Adverse Event Report Form within the CRF.

Adverse reaction (AR)

All untoward and unintended responses to a medicinal product related to any dose. The phrase 'responses to a medicinal product' means that a causal relationship between a study medication and an AE is at least a reasonable possibility, i.e., the relationship cannot be ruled out. All cases judged by either the reporting medically qualified professional or the Sponsor as having a reasonable suspected causal relationship to the study medication qualify as adverse reactions.

All ARs are recorded in the CRF and on the Adverse Event Report Form within the CRF.

<u>Serious adverse event (SAE)</u>

Any untoward medical occurrence or affect that at any dose:

- results in death,
- is life-threatening*
- requires hospitalisation¥ or prolongation of existing hospitalisation
- results in persistent or significant disability or incapacity
- is a congenital anomaly or birth defect
- important medical events**

¥ A hospitalisation meeting the regulatory definition for "serious" is any inpatient hospital admission that includes a minimum of an overnight stay in a health care facility. Any AE that does not meet one of the definitions of serious (e.g., A & E visit, outpatient surgery, or requires urgent investigation) may be considered by the Investigator to meet the "other significant medical hazard" criterion for classification as a serious adverse event.

*Regarding a life-threatening event, this refers to an event in which the subject was at risk of death at the time of the event; it does not refer to an event which hypothetically might have caused death if it were more severe.

**Some medical events may jeopardise the subject or may require an intervention to prevent one of the above characteristics/consequences. Such events (hereinafter referred to as 'important medical events') should also be considered as 'serious' in accordance with the definition.

All SAEs are recorded in the patients' medical notes, the CRF, on the Adverse Event Report Form within the CRF and on a Serious Adverse Event Report Form (HBT-GCP-FM-21).

<u>Severe adverse events</u>

The term 'severity' is used here to describe the intensity of a specific event. This has to be distinguished from the term 'serious'.

The severity of the AE should be graded according to the following definitions:

- *Mild:* An event that is easily tolerated by the subject, causing minimal discomfort and not interfering with everyday activities
- *Moderate:* An event that is sufficiently discomforting to interfere with normal everyday activities
- *Severe:* An event that prevents normal everyday activities

<u>Suspected unexpected serious adverse reactions (SUSAR)</u>

An adverse reaction, the nature or severity of which is not consistent with the applicable product information (e.g. Investigator's brochure for an unauthorised investigational medicinal product or summary of product characteristics (SmPC) for an authorised medicinal product.

14.2 Adverse event assessment

Assessment of seriousness

The Investigator should make an assessment of seriousness (see definition of seriousness in section 14.1).

Assessment of casualty

All adverse events judged by either the Investigator or the Sponsor as having a reasonable suspected causal relationship to an investigational medicinal product qualify as adverse reactions.

As the PARABLE study is blinded, the case should be assessed for causality assuming that the study drug under investigation caused the reaction (i.e. LCZ696).

The causality assessment given by the Investigator should not be downgraded by the Sponsor.

The Investigator/Sponsor must make an assessment of whether the AE/SAE is likely to be related to treatment according to the following definitions:

- *Unrelated:* Where an event is not considered to be related to the study medication.
- *Possible:* Although a relationship to the study medication cannot be completely ruled out, the nature of the event, the underlying disease, concomitant medication or temporal relationship make other explanations possible.
- *Probable:* The temporal relationship and absence of a more likely explanation suggest the event could be related to the study medication.

All AEs/SAEs judged as having a reasonable suspected causal relationship (e.g. possible, probable) to the study medication will be considered as ARs/SARs.

All AEs/SAEs judged as being related (e.g. possibly, probably) to an interaction between the study medication and another medication will also be considered to be ARs/SAR.

Alternative causes such as natural history of the underlying disease, concomitant therapy, other risk factors and the temporal relationship of the event to the treatment should be considered.

Assessment of severity
The Investigator will make an assessment of severity for each AE/SAE and record this on the Adverse Event Report Form according to one of the following categories:

- *Mild:* An event that is easily tolerated by the subject, causing minimal discomfort and not interfering with every day activities.
- *Moderate:* An event that is sufficiently discomforting to interfere with normal everyday activities.
- *Severe:* An event that prevents normal everyday activities.

Assessment of expectedness
The expectedness of an adverse reaction will be determined by the Sponsor according to the reference document which in this study is the Investigator's brochure for LCZ696 and the SmPC for valsartan.

14.3 Emergency unblinding procedures

See section 13.10.

14.4 Follow up of adverse events

It will be left to the Investigators clinical judgement whether or not an AE is of sufficient severity to require the subject’s removal from treatment. A subject may also voluntarily withdraw from treatment due to what he or she perceives as an intolerable AE. If either occurs, the subject must undergo an EOS assessment and be given appropriate care under medical supervision until symptoms cease or the condition becomes stable.

All SAEs that are encountered during the reporting period will be followed to their resolutions, until the Investigator assesses them as stable.

AEs considered related to the study medication as judged by the Investigator or Sponsor will be followed until resolution or until the subject/event is considered stable.

All related AEs that result in a subject’s withdrawal from the study or are present at the end of the study, should be followed up until a satisfactory resolution occurs.

Any pregnancy occurring during the clinical study and the outcome of the pregnancy should be recorded and followed-up for congenital abnormality or birth defect.

14.5 Documentation of adverse events by the Investigator

All AEs occurring during the study, observed by the Investigator or reported by the subject, whether or not attributed to the study medication are recorded in the appropriate section of the CRF.

All AEs are also recorded on an Adverse Event Report Form within the CRF.

In addition to recording the AE in the CRF and on an Adverse Event Report Form, all SAEs must be recorded on a Serious Adverse Event Report Form (HBT-GCP-FM-21). and in the patients' medical file.

The following information shall be recorded on the Adverse Event Report Form.

- AE event term
    - Whenever possible, recognized medical terms will be used to describe AEs rather than colloquialisms (for example, "influenza" rather than "flu") and abbreviations will be avoided.
- A description of the event
- Dates of onset and resolution
- The maximum severity of the AE (i.e. mild, moderate or severe)
- The relationship (causality) of the event to the study drug should be determined by the Investigator (Unrelated, possibly related, probably related)
- Any drug action taken by the Investigator to resolve the event:
    - None
    - Dose reduced
    - Dose increased
    - Withdrawn permanently
    - Withdrawn temporarily
    - Unknown
    - Other (which can include concomitant medication given, non-drug therapy given or hospitalized etc).
- Event outcome at resolution or time of last follow-up will be recorded as:
    - Recovered without sequelae
    - Recovered with sequelae
    - Not yet recovered
    - Unknown
    - Fatal
- Seriousness. AEs shall be assessed as serious or non-serious according to the definition in section 14.1.

AEs occurring secondary to other AEs (e.g. sequelae or complications) will be identified by the primary cause. A primary AE if clearly identifiable, generally

represents the most accurate clinical term to record. The Investigator will provide his/her opinion of which is the primary AE.

14.6 Reporting procedures for SAEs to the Sponsor

See Appendix 7 for an overview of this procedure.

All SAE information must be recorded on an SAE Report form and forwarded (by fax, email or in person) to the sponsor (Clinical Trial Project Manager) within 24 hours. Under no circumstances should this exceed **24 hours** following knowledge of the SAE.

Additional information received for a case (follow-up or corrections to the original case) need to be detailed on a new SAE form and sent expeditiously to the Sponsor.

The Sponsor will keep detailed records of all adverse events which are reported to him by the Investigator or Investigators and perform an evaluation with respect to seriousness, causality and expectedness. If the Sponsor disagrees with the Investigators assessment, both the opinion of the Investigator and the Sponsor should be provided with the report. The Sponsor should NEVER downgrade any assessment made by the Investigator.

14.7 Reporting procedures for SUSARs to the EC and Regulatory authorities

The Sponsor will report all SUSARs in parallel to both the competent authorities (the Health Products Regulatory Authority (HPRA) in Ireland) and the Clinical Trials module of the EudraVigilance database (EVCTM) and to the EC(s) concerned. For HPRA reporting, an electronic transmission will be sent via EudraVigilance.

As a back-up measure during times of non-availability of the clinical trials project manager, the reporter shall report the SUSAR to the HPRA directly using the online report form at the following address:

https://www.hpra.ie/homepage/about-us/report-an-issue/form2

This arrangement has been agreed with the Pharmacovigilance Department of the HPRA. They have requested that all SUSARs be transmitted to them in a timely manner in order to facilitate internal HPRA review and onward reporting by the HPRA to EudraVigilance. The following timelines therefore, must be adhered to:

- Any fatal/life threatening reports must reach the HPRA by Day 2.
- All other SUSARs by Day 7.

During any planned absences, the CTPM shall confirm in writing to the Principal Investigator and the Investigator team the process to be followed, including who to contact to carry out an assessment of expectedness. During any unplanned absences, the Research Director of the Heartbeat Trust should be contacted.

For EC reporting to the St Vincent's EC, a copy of the SAE Report Form along with a cover letter should be emailed to joan.mcdonnell@ucd.ie.

Timelines for reporting

- Fatal or life-threatening SUSARs must be reported within **7 days**.
- SUSARs which are not fatal and not life-threatening are to be reported within **15 days**.

The Sponsor will also inform all Investigators concerned of relevant information about SUSARs that could adversely affect the safety of subjects.

If the initial report is incomplete, e.g. if the Sponsor has not provided all the information/assessment within seven days, the Sponsor will submit a completed report based on the initial information within an additional eight days.

If significant new information on an already reported case is received by the Sponsor, the clock starts again at day zero, i.e. the date of receipt of new information. This information will be reported as a follow-up report within **15 days.**

14.8 SAE reporting to Novartis

See Appendix 8 for an overview of this procedure.

The Sponsor shall provide to Novartis details of all SAEs irrespective of causality within 24 hours of first notification of the SAE or subsequent follow-up information to the Sponsor.

Initial and follow-up reports shall be emailed or faxed directly to the Novartis Safety Desk in Ireland:

Novartis Ireland Limited<br>Drug Safety and Epidemiology<br>The Vista Building, Elm Park Business Park,<br>Merrion Road,<br>Dublin 4, D04, A9N6<br>Tel: +353 1 2601255<br>Fax: +353 1 2838777<br>Email:<br>CC: ct_processing.gbfr@novartis.com

To facilitate Novartis' compliance with the regulatory requirements, all suspected unexpected SAEs will be unblinded for regulatory reporting.

The Sponsor shall support Novartis in the following-up of all SAEs. The Sponsor shall perform SAE reconciliation according to Novartis SOPs and processes on an ongoing basis throughout the study.

SAEs will be reviewed on an ongoing basis by the Sponsor or its designee, with a summary included in the annual reports to regulatory agencies and analysis will be provided in the final study report.

14.9 Annual reports and line listings

In addition to the expedited reporting above, the Sponsor shall submit once a year throughout the clinical trial or on request, a safety report to the competent authority (the HPRA in Ireland) and EC. The annual safety report will be presented in the DSUR format as per ICH guideline E2F - Note for guidance on development safety update reports. This is a legal requirement. (See also SOP: HBT-GCP-SOP-09)

The Sponsor shall provide the St Vincent's EC with six monthly line listings of all SAEs (whether suspected or not, or unexpected or not) which have occurred over the reporting period.

Line listings are not routinely required by the HPRA. However, it should be noted that copies of these line listings may be requested and, as such, should be made available to the HPRA when required.

15. PROTOCOL ADHERENCE

Investigators ascertain they will apply due diligence to avoid protocol deviations.

If the Investigator feels a protocol deviation would improve the conduct of the study this must be considered a protocol amendment, and unless such an amendment is agreed upon by the Sponsor and approved by the applicable Health Authorities and EC, it cannot be implemented. Therefore, all suggested protocol deviations must be communicated to the Sponsor who will decide whether or not to submit an amendment to the protocol.

Once identified, the investigator shall record the deviation in the Deviation Log (HBT-GCP-FM-28) as per SOP HBT-GCP-SOP-05; 'Deviation Management'.

15.1 Protocol Amendments

Any change or addition to the protocol can only be made in a written protocol amendment that must be approved by the Sponsor, Health Authorities and EC. Only amendments that are required for subject safety may be implemented prior to approval. Notwithstanding the need for approval of formal protocol amendments, the Investigator is expected to take any immediate action required for the safety of any subject included in this study, even if this action represents a deviation from the protocol. In such cases, the Sponsor should be notified of this action and the EC informed within 10 working days.

16. STATISTICS

16.1 Description of statistical methods

If continuous variables are transformable to normal, they will be power-transformed and independent, two sample t-tests will be used for analysis of continuous variables. If data are not transformable to normal, non-parametric t-test equivalents (e.g. Wilcoxon signed rank and rank sum test, Mann-Whitney test and analysis of covariance (ANCOVA)) will be used. Chi-squared (or Fisher Exact) analyses will be used to compare categorical variables. Two-sided p values <0.05 will denote significance, with adjustment for multiplicity using Holm procedure. If required, missing data will be addressed using multiple imputation using chained equations as previously described (Ledwidge et al. JAMA. 2013;310(1):66-74) and results will be presented (primary and secondary end points) as pooled results from the multiple imputed data sets using a last-observation carried-forward analysis.

Demographics and clinical characteristics will be summarized with descriptive statistics (counts and percentages for categorical variables, or mean value ± the standard deviation for normally distributed continuous variables, or medians and interquartile ranges for non-normally distributed continuous variables).Repeated marker changes from baseline to 18 months will be analysed using ANOVA with repeated measures models.

Primary and secondary outcome measures will be performed both with and without adjustment for the effects of age, sex, diabetes, hypertension, obesity, and vascular disease. Further models will include adjustment for baseline measures of the outcome of interest (e.g. LAVI, BNP). Categorical endpoints will be analyzed using generalized linear modeling with a binomial outcome distribution for prevalence and a Poisson outcome distribution for incidence rate (adjusted for patient-years). The multivariable models will include both prespecified covariates (age, sex, diabetes, hypertension, obesity, and vascular disease) and those variables with univariate P values ≤ 0.25. The likelihood ratio test and the Nagelkerke R Square will be used to identify independent variables with low explanatory power and to assess the fit of each specification of the multiple regression models.

16.2 Determination of sample size

Based on the endpoint of change in LAVI over 18 months, the PARABLE trial aims to recruit 250 subjects (125 per arm). The current STOP-HF database includes 1499 subjects with cardiovascular risk factors. An additional cohort of just under 1000 subjects has been recruited from the STOP-HF outreach service and is available for screening and recruitment into the study. The STOP-HF Investigators are also working with other clinical trial units and can incorporate additional centres if required to achieve timely recruitment.

Sample size estimates were calculated from the current STOP-HF subject database (n=1499). A subsample of subjects with BNP > 50 pg/mL, LAVI > 28 mL/m^2, with normal EF (≥ 50%) and in normal sinus rhythm was drawn. This resulted in a population of 163 (11%) of the total with a population BNP of 125 pg/mL and a baseline mean LAVI of 36.2 mL/m^2 (standard deviation of 6.9 mL/m^2) determined by Doppler

Echocardiography. This LAVI is reassuringly similar to the baseline level observed by Solomon et al. (Lancet 2012; 380: 1387–95) in the PARAMOUNT study, which observed a 36-week drop in LAVI of 2.6 mL/m^2, and assuming the control group-shows no decrease in LAVI (as in Solomon et al. 2012 paper), the expected effect size in a similar study is 2.6/6.5 = 0.38. Setting alpha = 5% (two-tailed), beta = 20% and using an independent t-test to assess the change in LAVI over follow-up, the required number of subjects to detect this effect is 222 (111 per study arm), or 248 after accounting for a 10% dropout rate.

In a review of the expected effect size, once blinded echocardiography was available for 125 patients at the 9-month time point, it was noted that the actual standard deviation of baseline to 9-month change scores was 4.7. This means an expected effect size of at least (2.0/5.0) = 0.40. An effect size of 0.4 would require 96 patients in each group, suggesting that the study is adequately powered on LAVI assessed by echocardiography. In addition, the study has at least 80% power with a two-tailed alpha = 5% to detect a 6 g/m2 difference in LVMI change by CMRi, a 2.5 mL/m2 in LAVI change by CMRi, a 2-unit difference in E/e' or e' using Tissue Doppler measurements.

17. DIRECT ACCESS TO SOURCE DATA/DOCUMENTS

Direct access will be granted to authorised representatives from the Sponsor, host institution and the regulatory authorities to permit trial-related monitoring, audits and inspections.

18. DATA HANDLING AND RECORD KEEPING

18.1 Data collection, source documents and case report forms (CRFs)

Source documents for this study will include hospital records, procedure reports and data collection forms (e.g. questionnaires). These documents will be used to enter data on the CRFs.

A paper CRF will be used for this study. All data entered on CRFs must be entered legibly. If an error is made, the error will be crossed through with a single line in such a way that the original entry can still be read. The correct entry will then be clearly inserted and the alterations will be initialled and dated by the Investigator.

Data reported on the CRF that are derived from source documents must be consistent with the source documents or the discrepancies must be explained. In some instances it is acceptable to file a copy of the source document (e.g. blood report, ABPM read-out) in the study file and to cross-reference to the source document in the CRF.

The monitor must make certain that all data are completed on the CRF. After comparing the data to the source documents, the monitor will request correction/clarification from the Investigator.

After the last visit of the subject, the Investigator must attest the authenticity of the data collected in the CRF by signing the appropriate section of the CRF.

All documents will be stored safely in a confidential manner. On all study-specific documents, other than informed consent, the subject will be referred to by the subject study number/code.

18.2 Data reporting

Subjects will be identified by a study specific subject number in the database. The name and any other identifying detail will not be included in any study data electronic file.

19. RETENTION OF ESSENTIAL DOCUMENTS

Essential documents will be retained until at least 15 years after the publication of the clinical study report. These documents will be retained for a longer period however, if required by the applicable regulatory requirements or by agreement with the Sponsor. The Investigator should retain the trial-related essential documents as required by the applicable regulatory requirements and until the Sponsor informs the Investigator these documents are no longer necessary.

20. QUALITY CONTROL AND QUALITY ASSURANCE PROCEDURES

The study will be conducted in accordance with the current approved protocol, ICH good clinical practice (GCP), relevant regulations and applicable SOPs.

20.1 Before the study

The Investigator will allow the monitor to visit the site and facilities where the study will take place in order to ensure compliance with the protocol requirements.

Training sessions will be organised for the Investigators/study team on the study protocol.

20.2 During the study

For all participants who give informed consent, regardless of whether or not they receive any study drug, the Investigator designee will record participant identification data in the "Participant Identification List" (full name, initials, date of birth, participant identification code) or other similar list. The participant identification list will allow for the definite identification of any research participant that takes part in the study. A statement acknowledging the participation of a research participant in this clinical study will be also documented in the participant's medical file/notes.

To protect the research participant's identity a unique identification code (randomisation number/study ID) will be assigned by WebEz to each study participant and used in lieu of the participant's name when AEs and/or other study-related data are reported. This coded form of identification, instead of the participant’s name, will appear on all documents/databases and will be cross-referenced by the participant's

date of birth. Documents not for submission to Sponsor, such as the confidential participant identification list and the signed Informed Consent Forms, will be kept in strict confidence on site and will not be removed from site.

Personal information will be treated as confidential but may need to be reviewed by authorised representatives of the Sponsor - such as monitors and auditor/inspector from the ECs and the regulatory authorities. The participant's consent to direct access to his/her original medical records for data verification purposes will be obtained, via the informed consent process prior to that participant’s participation in the study.

The Investigator will permit the Sponsor, authorised agents of the Sponsor (such as monitors, auditors, etc.), the Contract Research Organization, and regulatory agency employees to enter and inspect any site where the drug or records pertaining to the drug are held, and to inspect and copy all records relating to an investigation, including research participant records.

The Investigator will make available all CRFs for review by the Sponsor, study monitor, and the regulatory agencies as necessary. To ensure the accuracy of data submitted, it is mandatory that representatives of the Sponsor and of the regulatory agencies have access to source documents (i.e. participant medical records or notes, charts, laboratory reports, etc.). Participant confidentiality will be protected at all times. The Investigator will allow the monitor to:

- inspect the site, the facilities and the material used for the study
- meet all members of his/her team involved in the study
- consult all of the documents relevant to the study
- have access to the CRFs
- check that the CRFs have been filled out correctly
- directly access source documents for comparison of data therein with the data in the CRFs
- verify that the study is carried out in compliance with the protocol and local regulatory requirements

The study Investigators will adhere to hospital protocols pertaining to healthcare record use and storage. The Investigators and authorised designees will ensure that the confidentiality of the participants’ data is preserved. On CRFs or any other documents submitted to the Sponsor’s designee, the participant will be identified not by their names, but by their coded form of identification.

21. AUDITS AND INSPECTIONS

The trial may be subject to internal or external auditing or inspection procedure to ensure adherence to GCP. Access to all trial-related documents will be given at that time.

A quality assurance audit may be conducted by the Sponsor or its agent at any time during, or shortly after, the study. The Investigator will permit an independent audit by an auditor mandated by Sponsor, after reasonable notice. The purpose of an audit is to confirm that the study is conducted as per protocol, GCP and applicable regulatory requirements, that the rights and well-being of the patients enrolled have been protected, and that the data relevant for the evaluation of the IMP have been captured, processed and reported in compliance with the planned arrangements. The Investigator will permit direct access to all study documents, drug accountability records, medical records and source data.

Regulatory authorities may perform an inspection of the study up to several years after its completion. If an inspection is announced the Sponsor will be informed immediately.

22. ETHICAL CONSIDERATIONS

22.1 Ethical conduct of the study

This study will be carried out in compliance with the Study Protocol and in accordance with the Sponsors SOPs. These are designed to ensure adherence to GCP guidelines, as described in:

- International Conference on Harmonization (ICH) of Technical Requirements for Registration of Pharmaceuticals for Human Use Harmonized Tripartite Guidelines for Good Clinical Practice 1996
- EU CT Directive 2001/20/EC
- GCP Commission Directive 2005/28/EC
- Declaration of Helsinki, concerning medical research in humans (1964) including all amendments up to and including the 2013 revision
- National laws

The Investigator agrees, when signing the study protocol, to adhere to the instructions and procedures described in it and to the principles of GCP to which it conforms. The regulatory permission to perform the study will be obtained in accordance with applicable regulatory requirements. All ethical and regulatory approvals must be available before a patient is exposed to any study-related procedure, including screening tests to determine eligibility.

22.2 Declaration of Helsinki

The Sponsor will ensure that this study is conducted in accordance with the ethical principles that have their origins in the Declaration of Helsinki.

22.3 Good Clinical Practice

This study will be conducted in accordance with GCP, as defined by the ICH and in accordance with the ethical principles underlying EU Directive 2001/20/EC and 2005/28/EC.

22.4 Approvals

Before initiating this study, the protocol, SmPC, Investigators Brochure, Patient Information Leaflet and Informed Consent Form, and any other written information to be given to participants will be reviewed and approved by the competent authority and the EC.

A signed and dated statement that all documents submitted for review have been approved by the EC will be given to the Sponsor or designee before the study can commence at a site. The membership and the constitution of the EC who approved the documents will be also supplied to the Sponsor or designee.

The Sponsor/designee will submit and obtain approval from the above parties for substantial amendments to the original approved documents.

Modifications of the signed protocol are only possible by approved protocol amendments and with the agreement of all responsible persons. The procedure for approval of a protocol amendment is identical to that for approval of the protocol. All substantial protocol amendment must be submitted to the Regulatory Authority and EC for approval. The changes in the protocol must not be instituted until the amendment and the revised informed consent (if appropriate) has been reviewed and received approval / favourable response from both the Regulatory Authority and EC. A protocol amendment intended to eliminate an apparent immediate hazard to participants may be implemented immediately providing that both the Regulatory Authority and EC are notified as soon as possible, and an approval is requested. Non-substantial protocol amendments may be implemented immediately.

22.5 Informed consent

The Investigator or his/her designee shall obtain written informed consent from each participant before his/her participation in the study and before any study related procedure is undertaken. Therefore, informed consent shall be obtained at Visit 1 before any study related procedure is performed. Prior to this, the Investigator or his/her delegate must inform each participant of the objectives, benefits, risks and requirements imposed by the study, as well as the nature of the study drugs. Informed consent must also be obtained for each patient to allow biobanking of blood samples.

A separate signature page is included on the Participant Information and Consent Form for this purpose. Only a qualified medical doctor may obtain informed consent.

The participant will be provided with an information and consent form in clear, simple language. He/she must be allowed ample time to inquire about details of the study and to decide whether or not to participate in the study. The Investigator shall answer all questions the patient may have about the study.

Two original information and consent forms must be completed, dated and signed personally by the participant and by the person responsible for collecting the informed consent. The participant will be given one signed original information and consent form, the second original will be kept by the Investigator.

Any change to the information and consent form constitutes an amendment to this document and must be submitted for approval to the EC, and if applicable to the Regulatory Authorities. Such amendments may only be implemented after written approval of the EC has been obtained and compliance with the local regulatory requirements, with the exception of an amendment required to eliminate an immediate risk to the study participants.

Each participant affected by the amendment or an independent witness must complete, date and sign two originals of the new version of the information and consent form together with the person who conducted the informed consent discussion. He/she will receive one signed original amendment to the information and consent form.

The obtaining of informed consent and any pre-consent discussions should be documented in the patients' medical notes. The following should be recorded in the subject's medical file:

- The date that the Informed Consent Form was given to the subject
- The date written informed consent was obtained
- The name of the person obtaining informed consent
- Any other relevant information regarding the consent process

For every patient recruited into the PARABLE clinical trial, a notification should be placed on the inside of the front cover of the patients' medical notes in order to identify the clinical trial. (The notification should record the study name, patients study ID, consent date, the 'end of patient participation date' which should be completed at visit 11, the name of the Principal Investigator and contact number of the research team).

22.6 Subject confidentiality

The trial staff will ensure that the participants' anonymity is maintained. The participants will be identified only by initials and a subject's identification number on the CRF and any database. All documents will be stored securely. The study will comply with the Data Protection Act.

23. FINANCING AND INSURANCE/INDEMNITY

The Heartbeat Trust is the Sponsor and it will ensure that every Investigator is covered by a Clinical Trial Insurance, which shall remain in effect for the duration of the Agreement. All Investigators are qualified and practicing physicians and are thus insured by the clinical indemnity scheme.

24. CLINICAL STUDY REPORT AND PUBLICATION POLICY

24.1 Clinical study report

The study report will be drafted by the Clinical Trials Manager of the HBT in compliance with the Sponsor's SOP.

The Sponsor and the Chief Investigator must mutually agree on the final version. One copy of the final report must be dated and signed by the Chief Investigator and the Legal Representative of the Sponsor.

24.2 Publication policy

The results of this study will be published in a high-quality clinical journal. The results of this study may provide the impetus for further research into the management of asymptomatic patients with risk factors for heart failure with LCZ696.

The responsibility for publication of the data obtained from this study lies with the Investigator. Neither the Sponsor, nor any of the study funders, will have any involvement in data collection, data review, data analysis or preparation of the publication manuscript or in the decision to submit for publication. Submission of any publication manuscript may take place only with the permission of Prof. Kenneth McDonald, Consultant Cardiologist, and Dr Mark Ledwidge, Director of Research, St. Vincent's University Hospital, Dublin 4.

25. STUDY PLANNING AND SCHEDULE

The project milestones are as follows:

1. Clinical trial application to the HPRA
2. St Vincent's University Hospital Ethics application
3. Selection of suitable subjects from the STOP-HF data base and STOP-HF Service in St Michael's Hospital
4. Informed consent from subjects
5. Recruitment of patients and inclusion in the study
6. Final evaluation
7. Data analyses
8. Manuscript preparation
9. Publication and presentation of results

25.1 Timelines and study duration

Start date: FPFV: 16 December 2015

End date:

- 18-month treatment period: LPFV: December 2018. FPLV: Oct 2017 and LPLV: December 2020
- 9- month follow-up (regression) substudy: FPFV: July 2019; LPLV: May 2021

Study Report date: CSR to be made available within 12 months after study completion.

26. APPENDICES

Appendix 1. Montreal Cognitive Assessment (MoCA)

Montreal Cognitive Assessment Basic_MoCA.pdf

Appendix 2. Standardised Mini-Mental State Examination SMMSE)

Standardised MMSE.pdf

Appendix 3. EuroQoL (EQ-5D-5L) Health Questionnaire

Subject study ID:	Subject initials:	
Date of test:	Visit number:	
☐ Baseline	☐ 9 months	☐ 18 months

Under each heading, please tick the ONE box that best describes your health TODAY.

MOBILITY

I have no problems in walking about ☐

I have slight problems in walking about ☐

I have moderate problems in walking about ☐

I have severe problems in walking about ☐

I am unable to walk about ☐

SELF-CARE

I have no problems washing or dressing myself ☐

I have slight problems washing or dressing myself ☐

I have moderate problems washing or dressing myself ☐

I have severe problems washing or dressing myself ☐

I am unable to wash or dress myself ☐

USUAL ACTIVITIES *(e.g. work, study, housework, family or leisure activities)*

I have no problems doing my usual activities ☐

I have slight problems doing my usual activities ☐

I have moderate problems doing my usual activities ☐

I have severe problems doing my usual activities ☐

I am unable to do my usual activities ☐

PAIN / DISCOMFORT

I have no pain or discomfort ☐

I have slight pain or discomfort ☐

I have moderate pain or discomfort ☐

I have severe pain or discomfort ☐

I have extreme pain or discomfort ❑

ANXIETY / DEPRESSION

I am not anxious or depressed ❑

I am slightly anxious or depressed ❑

I am moderately anxious or depressed ❑

I am severely anxious or depressed ❑

I am extremely anxious or depressed ❑

- We would like to know how good or bad your health is TODAY.
- This scale is numbered from 0 to 100.
- 100 means the <u>best</u> health you can imagine.
  0 means the <u>worst</u> health you can imagine.
- Mark an X on the scale to indicate how your health is TODAY.
- Now, please write the number you marked on the scale in the box below.

YOUR HEALTH TODAY =

The best health you can imagine

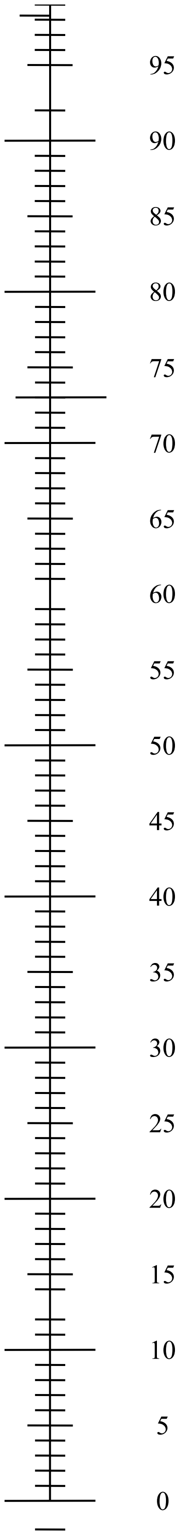

The worst health you can imagine

Appendix 4. Montreal Cognitive Assessment (MoCA) Version 3. Administration and Scoring Instructions

The Montreal Cognitive Assessment (MoCA) was designed as a rapid screening instrument for mild cognitive dysfunction. It assesses different cognitive domains: attention and concentration, executive functions, memory, language, visuoconstructional skills, conceptual thinking, calculations, and orientation.

Time to administer the MoCA is approximately 10 minutes.

The total possible score is 30 points; a score of 26 or above is considered normal.

1. **Alternating Trail Making:**

Administration: The examiner instructs the subject: "*Please draw a line, going from a number to a letter in ascending order. Begin here [point to (1)] and draw a line from 1 then to A then to 2 and so on. End here [point to (E)].*"

Scoring: Allocate one point if the subject successfully draws the following pattern: 1 –A- 2- B- 3- C- 4- D- 5- E, without drawing any lines that cross. Any error that is not immediately self-corrected earns a score of 0.

2. **Visuoconstructional Skills (Cylinder):**

Administration: The examiner gives the following instructions, pointing to the cylinder: *"Copy this drawing as accurately as you can, in the space below".*

Scoring: One point is allocated for a correctly executed drawing.

- Drawing must be three-dimensional
- All lines/ovals are drawn
- No line is added
- The horizontal lines are relatively parallel.
- The objects at the end must be ovals rather than circles.
- The horizontal lines must touch the top/bottom of the ovals.

A point is not assigned if any of the above-criteria are not met.

3. **Visuoconstructional Skills (Clock):**

Administration: Indicate the right third of the space and give the following instructions: *"Draw a clock. Put in all the numbers and set the time to ten past nine".*

Scoring: One point is allocated for each of the following three criteria:

- Contour (1 pt.): the clock face must be a circle with only minor distortion acceptable (e.g., slight imperfection on closing the circle);
- Numbers (1 pt.): all clock numbers must be present with no additional numbers; numbers must be in the correct order and placed in the approximate quadrants on the clock face; Roman numerals are acceptable; numbers can be placed outside the circle contour;
- Hands (1 pt.): there must be two hands jointly indicating the correct time; the hour hand must be clearly shorter than the minute hand; hands must be centred within the clock face with their junction close to the clock centre.

A point is not assigned for a given element if any of the above-criteria are not met.

4. **Naming:**

Administration: Beginning on the left, point to each figure and say: *"Tell me the name of this animal"*.

Scoring: One point each is given for the following responses: (1) donkey (or mule); (2) pig (or hog); (3) kangaroo.

5. **Memory:**

Administration: The examiner reads a list of 5 words at a rate of one per second, giving the following instructions: *"This is a memory test. I am going to read a list of words that you will have to remember now and later on. Listen carefully. When I am through, tell me as many words as you can remember. It doesn't matter in what order you say them."*

Mark a check in the allocated space for each word the subject produces on this first trial. When the subject indicates that (s)he has finished (has recalled all words), or can recall no more words, read the list a second time with the following instructions: *"I am going to read the same list for a second time. Try to remember and tell me as many words as you can, including words you said the first time."*

Put a check in the allocated space for each word the subject recalls after the second trial. At the end of the second trial, inform the subject that (s)he will be asked to recall these words again by saying, *"I will ask you to recall those words again at the end of the test."*

Scoring: No points are given for Trials One and Two. Scoring is based on the delayed recall trial.

6. **Attention:**

Forward Digit Span: Administration: Give the following instruction: *"I am going to say some numbers and when I am through, repeat them to me exactly as I said them"*. Read the five number sequence at a rate of one digit per second.

Backward Digit Span: Administration: Give the following instruction: *"Now I am going to say some more numbers, but when I am through you must repeat them to me in the backwards order."* Read the three number sequence at a rate of one digit per second.

Scoring: Allocate one point for each sequence correctly repeated, (N.B.: the correct response for the backwards trial is 4-7-1).

7. **Vigilance:**

Administration: The examiner reads the list of letters at a rate of one per second, after giving the following instruction: *"I am going to read a sequence of letters. Every time I say the letter A, tap your hand once. If I say a different letter, do not tap your hand"*.

Scoring: Give one point if there is zero to one errors (an error is a tap on a wrong letter or a failure to tap on letter A).

8. Serial 7s:

Administration: The examiner gives the following instruction: *"Now, I will ask you to count by subtracting 7 from 80, and then, keep subtracting 7 from your answer until I tell you to stop."* Give this instruction twice if necessary.

Scoring: This item is scored out of 3 points. Give no (0) points for no correct subtractions, 1 point for one correction subtraction, 2 points for two-to-three correct subtractions, and 3 points if the participant successfully makes four or five correct subtractions.

Count each correct subtraction of 7 beginning at 80. Each subtraction is evaluated independently; that is, if the participant responds with an incorrect number but continues to correctly subtract 7 from it, give a point for each correct subtraction. For example, a participant may respond "72 – 65 – 58 – 51 – 44" where the "72" is incorrect, but all subsequent numbers are subtracted correctly. This is one error and the item would be given a score of 3.

9. **Sentence repetition:**

Administration: The examiner gives the following instructions: "*I am going to read you a sentence. Repeat it after me, exactly as I say it [pause]:* ***She heard his lawyer was the one to sue after the accident.***"

Following the response, say: *"Now I am going to read you another sentence. Repeat it after me, exactly as I say it [pause]:* ***The little girls who were given too much candy got stomach aches."***

Scoring: Allocate 1 point for each sentence correctly repeated. Repetition must be exact. Be alert for errors that are omissions (e.g., omitting "too much") and substitutions/additions (e.g., " . . . his lawyer sued after . . ."; "the girls"), altering plurals, etc.).

10. **Verbal fluency:**

Administration: The examiner gives the following instruction: *"Tell me as many words as you can think of that begin with a certain letter of the alphabet that I will tell you in a moment. You can say any kind of word you want, except for proper nouns (like Peter or Paris), numbers, or words that begin with the same sound but have a different suffix, for example, love, lover, loving. I will tell you to stop after one minute. Are you ready? [Pause] Now, tell me as many words as you can think of that begin with the letter B. [time for 60 sec]. Stop."*

Scoring: Allocate one point if the subject generates 11 words or more in 60 sec. Record the subject's response in the bottom or side margins.

11. **Abstraction:**

Administration: The examiner asks the subject to explain what each pair of words has in common, starting with the example: *"Tell me how an orange and a banana are alike".* If the subject answers in a concrete manner, then say only one additional time: "Tell me another way in which those items are alike". If the subject does not give the appropriate response (fruit), say, *"Yes, and they are also both fruit."* Do not give any additional instructions or clarification. After the practice trial, say: *"Now, tell me how an eye and an ear are alike".*

Following the response, administer the second trial, saying: *"Now tell me how a trumpet and a piano are alike".* Do not give any additional instructions or prompts.

Scoring: Only the last two item pairs are scored. Give 1 point to each item pair correctly answered. The following responses are acceptable:

- eye-ear = sensory organs, parts of the head, parts of the body;
- trumpet-piano = musical instruments, you can play them.

The following responses are **not** acceptable: eye-ear = parts of the face.

12. **<u>Delayed recall:</u>**

Administration: The examiner gives the following instruction: *"I read some words to you earlier, which I asked you to remember. Tell me as many of those words as you can remember."* Make a check mark (√) for each of the words correctly recalled spontaneously without any cues, in the allocated space.

<u>Scoring:</u> Allocate 1 point for each word recalled freely <u>without any cues</u>.

13. **<u>Orientation:</u>**

<u>Administration:</u> The examiner gives the following instructions: *"Tell me the date today"*. If the subject does not give a complete answer, then prompt accordingly by saying: *"Tell me the [year, month, exact date, and day of the week]."* Then say: "*Now, tell me the name of this place, and which city it is in."*

<u>Scoring:</u> Give one point for each item correctly answered. The subject must tell the exact date and the exact place (name of hospital, clinic, unit). No points are allocated if subject makes an error of one day for the day and date.

TOTAL SCORE: Sum all sub-scores listed on the right-hand side. Add one point for an individual who has 12 years or fewer of formal education, for a possible maximum of 30 points. A final total score of 26 and above is considered normal.

Appendix 5. Adverse Event Handling. Investigator Responsibilities. Flow Diagram

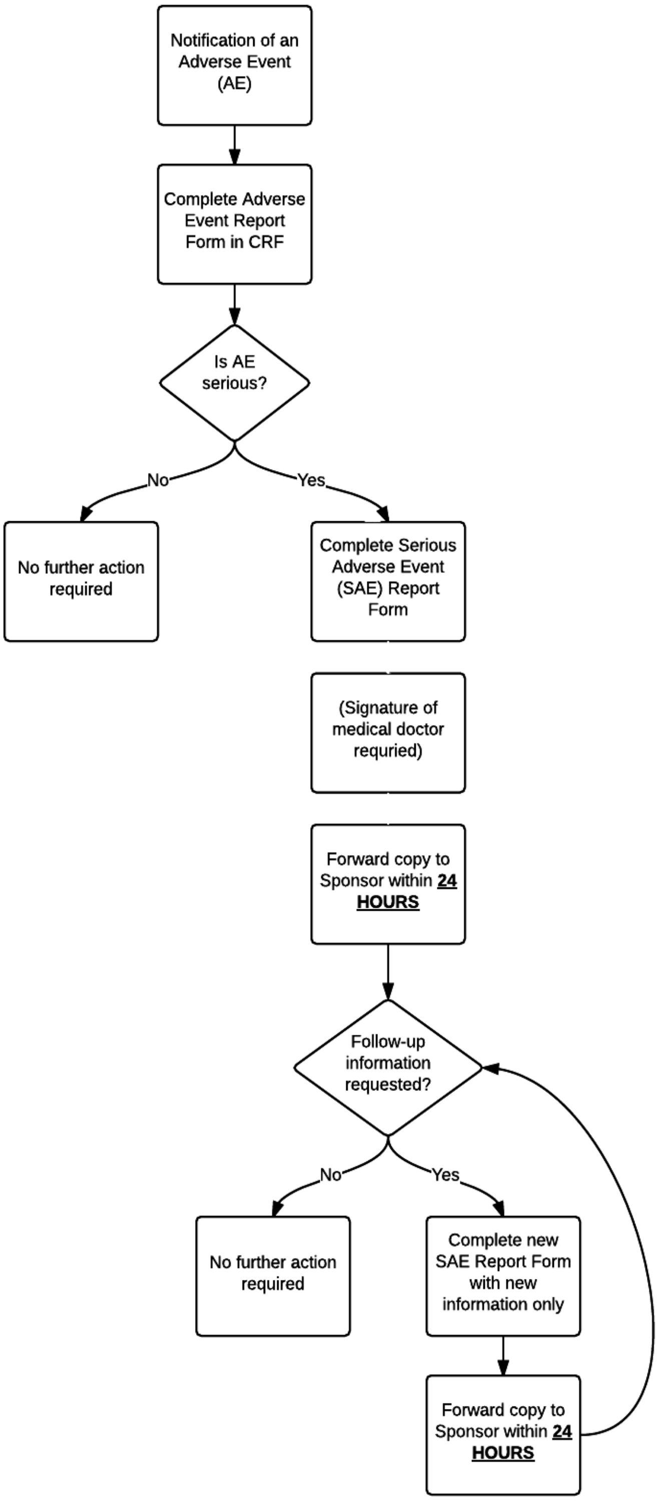

Appendix 6. Adverse Event Handling. Sponsor Responsibilities. Flow Diagram

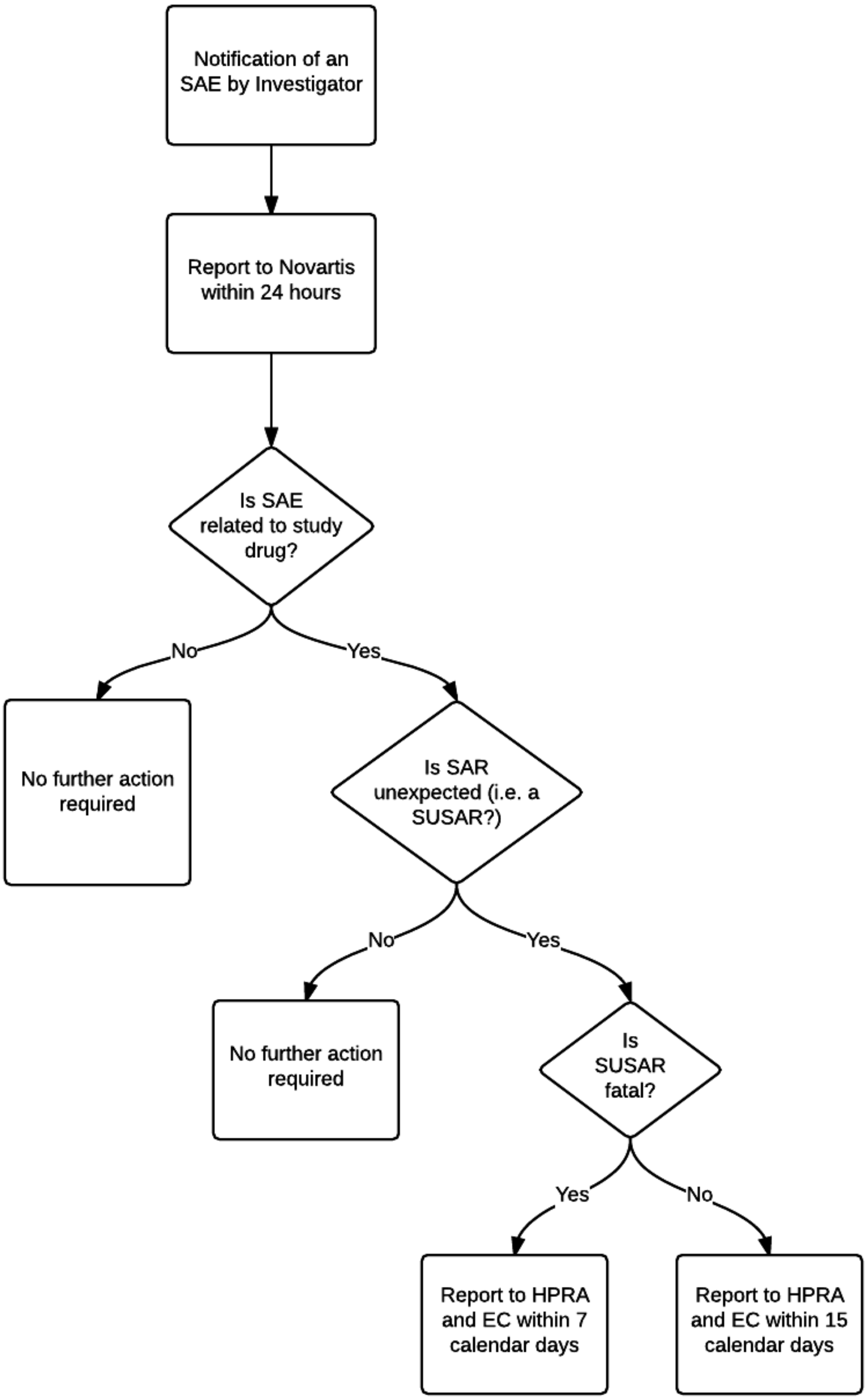

Appendix 7. Clinically notable laboratory values and vital signs

Clinically notable laboratory abnormalities for selected tests based on a percent change from baseline:

Haematology

RBC count	>50% increase	>20% decrease
Haemoglobin	>50% increase	>20% decrease
Haematocrit	>50% increase	>20% decrease
WBC count	>50% increase	>50% decrease
Platelet count	>75% increase	>50% decrease

Blood Chemistry

ALT (SGPT)	>150% increase	
AST (SGOT)	>150% increase	
BUN	>50% increase	
Creatinine	>50% increase	
Total bilirubin	>100% increase	
CPK	>300% increase	
Alkaline phosphatase	>100% increase	
Sodium	>5% decrease	
Potassium	>20% increase	>20% decrease
Chloride >10% increase,		>10% decrease
Calcium >10% increase,		>10% decrease
Uric acid >50% increase		

Appendix 8. Treatment guidelines

Treatment guidelines for hyperkalaemia (serum K+ > 5.2 mEq/L)

General principles: It is recommended that any subject with a serum K^+ > 5.2 mEq/L following randomization may require frequent checks of potassium concentration until it is clear that the potassium concentration is stable and not rising into the range of concern (> 5.5 and < 6.0 mEq/L) or potential danger (> 6.0 mEq/L).

Investigators should not randomize a subject with a serum K^+ > 5.2 mEq/L at Visit 1 (screening) and follow the other measures outlined below if K^+ increases to values > 5.2 mEq/L after randomization.

Corrective action recommended for management of hyperkalaemia

K^+ > 5.2 and ≤ 5.5 mEq/L

- If necessary, confirm potassium concentration was obtained in a non-haemolysed sampled. If it is confirmed that questionable results were obtained from haemolysed specimen, redraw blood sample for K^+ measurement and caution should be taken to minimize the incidence of haemolysis by proper blood sample collection and handling
- Reinforce low potassium diet and restriction of food/drinks with high potassium content (e.g. orange juice, melon, bananas, low-salt substitutes, etc.)
- Review medical regimen (including dietary supplements and over-the-counter medications) for agents known to cause hyperkalaemia. Consider reduction in dose or discontinuation of these agents:
    - Confirm that potassium-sparing diuretics and potassium supplements aren't being used
    - Salt substitutes
    - Non-steroidal anti-inflammatory drugs (NSAIDs)
    - Cyclo-oxygenase-2 (COX-2) inhibitors
    - Trimethoprim and trimethoprim-containing combination products such as trimethoprim/sulfamethoxazole
    - Herbal Supplements: (for xample, Noni juice, alfalfa (Medicago sativa), dandelion (Taraxacum officinale), horsetail (Equisetum arvense), nettle (Urtica dioica), milkweed, lily of the valley, Siberian ginseng, hawthorn berries)

K^+ > 5.5 and ≤ 6.0 mEq/L

- If necessary, confirm potassium concentration was obtained in a non-hemolyzed sample
- Apply all measures outlined for K^+ 5.2 and ≤ 5.5 mEq/L
- Repeat K^+ measurement after 1-3 days
- Discontinue the study drug if a repeated K^+ > 5.5 mEq/L

K^+ > 6.0 mEq/L

- Immediately discontinue study drug
- Confirm potassium concentration in a non-haemolysed sample
- Urgently evaluate subject and treat hyperkalaemia as clinically indicated
- Apply all measures outlined for K^+ > 5.2 and ≤ 6.0 mEq/L

Guidelines for the management of hypotension

1. Treatment should not be initiated if systolic BP <100mmHg at screening or baseline. A lower starting dose (Level 1) should be considered for patients with systolic BP ≥100 to 110mmHg.
2. Investigator should monitor blood pressure closely throughout the study
3. If systolic BP falls to ≤95mmHg (or symptomatic hypotension occurs at any BP):

- Correct any treatable cause, e.g. hypovolemia
- If symptomatic hypotension persists, consider down titrating or stopping other hypotensive medications, and/or study drug as appropriate.
- If symptomatic hypotension persists during two consecutive office visits or as judged by the Investigator, discontinue the study drug.

Guidelines for the management of renal dysfunction

General principles: Some subjects may develop renal dysfunction after study drug administration. Two types of response to serum creatinine increase are described:

Surveillance situation

If, at any moment after recruitment, serum creatinine increases by ≥50% from baseline (Visit 1), the Investigator will check for potentially reversible cases of renal dysfunction such as:

- NSAID intake, diuretics, antibiotics, or other treatments known to affect creatinine
- Volume decrease
- Urinary infection
- Urinary tract obstruction
- Study medication

The Investigator should consider discontinuing any medication known to affect the renal function and should repeat the serum creatinine after 5-7 days.

Action situation

If serum creatinine >3.0 mg/dL or increases by ≥100% from baseline (Visit 1), the Investigator will discontinue study drug and check for potentially reversible causes of renal dysfunction (see above). Thereafter, serum creatinine should be monitored until its resolution.

Appendix 9. PARABLE. Nine-month follow-up study

1. Objectives and endpoints

The overall objective of the nine-month follow-up study is to evaluate if the treatment effects observed during the 18-month PARABLE study persist nine-months after study drug discontinuation (see Exploratory Objective No 4 in Section 10.1).

Secondary and exploratory endpoints shall be evaluated in this sub-set of subjects (see Exploratory Endpoint No 10 Section 10.2).

2. Eligibility criteria

All PARABLE participants that completed the 18-month follow-up period, and did not discontinue the study drug prematurely, will be eligible for inclusion in the follow-up study.

3. Informed Consent

The procedure for obtaining informed consent is outlined in Section 22.5 of the main PARABLE protocol. Informed consent shall be obtained from the participant prior to any study related assessment or procedure been conducted using the Participant Information Leaflet and Informed Consent Form for the follow-up study (HBT-GCP-FM-39).

4. Assessments and procedures

The following assessments and procedures will be carried out at the nine-month follow-up visit in the same manner they were conducted during the main PARABLE study (see section 12.4) of the study protocol).

- Physical exam
  - Vital size (BP and pulse, height, weight and waist/hip circumference)
  - Biochemistry
    - Urea & Electrolytes (U&E) including eGFR
    - Liver Function Tests (LFT)
    - Alkaline Phosphatase
    - Lipid profile
    - HbA1c
    - Iron studies
  - Haematology
    - Full blood count

- Urine for second messenger cGMP and ANP*
- Blood for biomarkers*
- Serum BNP and NT-proBNP
- Ambulatory blood pressure monitoring
- 12-lead ECG
- Echo-Doppler studies

- QOL questionnaire (EuroQol) and two cognitive function assessment tools

*cryovials will be labelled with the following information:

- PAR-XXXX (where XXXX is the participants PARABLE study ID)
- The date the sample was taken
- The type of sample (e.g. whole blood, lithium heparin, urine etc)
- Timepoint: 9M-FU

REFERENCES

1. Ledwidge, M., et al., *Natriuretic peptide–based screening and collaborative care for heart failure: The stop-hf randomized trial.* JAMA, 2013. **310**(1): p. 66-74.
2. Huelsmann, M., et al., *PONTIAC (NT-proBNP Selected PreventiOn of cardiac eveNts in a populaTion of dIabetic patients without A history of Cardiac disease): A Prospective Randomized Controlled Trial.* Journal of the American College of Cardiology, 2013. **62**(15): p. 1365-1372.
3. Phelan D, et al., *Modest Elevation in BNP in Asymptomatic Hypertensive Patients Reflects Sub-Clinical Cardiac Remodeling, Inflammation and Extracellular Matrix Changes.* PLoS ONE, 2012. **7**(11): p. e49259.
4. Potter, L.R., S. Abbey-Hosch, and D.M. Dickey, *Natriuretic Peptides, Their Receptors, and Cyclic Guanosine Monophosphate-Dependent Signaling Functions.* Endocrine Reviews, 2006. **27**(1): p. 47-72.
5. Gardner, D.G., et al., *Molecular Biology of the Natriuretic Peptide System: Implications for Physiology and Hypertension.* Hypertension, 2007. **49**(3): p. 419-426.
6. Watson CW, Glezeva N, Horgan S, Gallagher J, Phelan D, McDonald K, Tolan M, Baugh B, Collier P, Ledwidge M *Atrial tissue pro-fibrotic M2 macrophage marker CD163+, gene expression of procollagen and B-Type Natriuretic peptide*. J Am Heart Assoc. 2020;9:e013416.
7. Hogg, K., K. Swedberg, and J. McMurray, *Heart failure with preserved left ventricular systolic function: epidemiology, clinical characteristics, and prognosis.* Journal of the American College of Cardiology, 2004. **43**(3): p. 317-327..
8. Querejeta, R., et al., *Increased Collagen Type I Synthesis in Patients With Heart Failure of Hypertensive Origin: Relation to Myocardial Fibrosis.* Circulation, 2004. **110**(10): p. 1263-1268.
9. Martos, R., et al., *Diastolic Heart Failure: Evidence of Increased Myocardial Collagen Turnover Linked to Diastolic Dysfunction.* Circulation, 2007. **115**(7): p. 888-895.
10. Ahmed, S.H., et al., *Matrix Metalloproteinases/Tissue Inhibitors of Metalloproteinases: Relationship Between Changes in Proteolytic Determinants of Matrix Composition and Structural, Functional, and Clinical Manifestations of Hypertensive Heart Disease.* Circulation, 2006. **113**(17): p. 2089-2096.
11. Nishikimi, T., N. Maeda, and H. Matsuoka, *The role of natriuretic peptides in cardioprotection*. Vol. 69. 2006. 318-328.
12. Solomon, S.D., et al., *The angiotensin receptor neprilysin inhibitor LCZ696 in heart failure with preserved ejection fraction: a phase 2 double-blind randomised controlled trial.* The Lancet. **380**(9851): p. 1387-1395.
