# Supplementary material for: Effect of Sacubitril/Valsartan vs Valsartan on Left Atrial Volume in Patients With Pre–Heart Failure With Preserved Ejection Fraction: The PARABLE Randomized Clinical Trial
Source: JAMA Cardiol. 2023 Mar 8;8(4):366–75. doi: 10.1001/jamacardio.2023.0065 (PMC9996460; doi:10.1001/jamacardio.2023.0065)
Supplement: Supplement 2. — eMethods 1. Trial design, Oversight, Investigational Medicinal Product and Randomisation eTable 1. PARABLE Inclusion and Exclusion Criteria eFigure 1. Design of the PARABLE trial eMethods 2. Medication Titration Protocol eTable 2. Study Titration Schedule eMethods 3. Description of Study Assessments eTable 3. Overview of Study Assessments eMethods 4. Detailed Description of Sample Size Calculations and Statistical Methods eTable 4. Detailed Cardiometabolic Medications at Baseline eTable 5. Detailed Cardiometabolic Medications at Follow up eTable 6. Additional Biochemistry at Baseline eTable 7. Additional Biochemistry at Follow Up eFigure 2. Change in per-protocol maximal left atrial volume index using cardiac MRI eFigure 3. Time to first serious adverse cardiovascular event eTable 8. Serious Adverse Event (SAE) over the study data by System-Organ-Class [file jamacardiol-e230065-s002.pdf]

Supplemental Online Content

Ledwidge M, Dodd JD, Ryan F, et al. Association of sacubitril/valsartan vs valsartan with left atrial volume in patients with pre–heart failure with preserved ejection fraction: the PARABLE randomized clinical trial. *JAMA Cardiol*. Published online March 8, 2023. doi:10.1001/jamacardio.2023.0065

eMethods 1. Trial design, Oversight, Investigational Medicinal Product and Randomisation

eTable 1. PARABLE Inclusion and Exclusion Criteria

eFigure 1. Design of the PARABLE trial

eMethods 2. Medication Titration Protocol

eTable 2. Study Titration Schedule

eMethods 3. Description of Study Assessments

eTable 3. Overview of Study Assessments

eMethods 4. Detailed Description of Sample Size Calculations and Statistical Methods

eTable 4. Detailed Cardiometabolic Medications at Baseline

eTable 5. Detailed Cardiometabolic Medications at Follow up

eTable 6. Additional Biochemistry at Baseline

eTable 7. Additional Biochemistry at Follow Up

eFigure 2. Change in per-protocol maximal left atrial volume index using cardiac MRI

eFigure 3. Time to first serious adverse cardiovascular event

eTable 8. Serious Adverse Event (SAE) over the study data by System-Organ-Class

This supplemental material has been provided by the authors to give readers additional information about their work.

© 2023 Ledwidge M et al. *JAMA Cardiology.*

Table of Contents

eMethods 1. Trial design, Oversight, Investigational Medicinal Product and Randomisation. *5*

eTable 1. PARABLE Inclusion and Exclusion Criteria 5

1.1 Diagnosis of Type 2 diabetes 7

eFigure 1. Design of the PARABLE trial 8

1.2 Trial Oversight 8

1.3 Investigational Medicinal Product 9

1.4 Randomisation 9

eMethods 2. Medication Titration Protocol *10*

eTable 2. Study Titration Schedule 10

2.1 Overview of Titration 10

2.2 Treatment guidelines for hyperkalaemia (serum K+ > 5.2 mEq/L) 12

2.3 Treatment guidelines for the management of renal dysfunction 13

2.4 Treatment guidelines for study drug restart after temporary treatment interruption 14

eMethods 3. Description of Study Assessments *15*

3.1 Laboratory parameters 15

3.2 Estimated Glomerular Filtration Rate assessments 15

eTable 3. Overview of Study Assessments 16

3.3 Physical exam 18

3.4 Vital signs 18

3.5 Height, weight and waist/hip circumference 18

3.6 Electrocardiography 18

3.7 Ambulatory blood pressure monitoring (ABPM) 18

3.8 Serum BNP and NT-proBNP 19

3.9 Echo-Doppler studies 19

3.10 Cardiac magnetic resonance imaging 21

eMethods 4. Detailed Description of Sample Size Calculations and Statistical Methods ... *23*

4.1 Covid-19 Procedures 24

eTable 4. Detailed Cardiometabolic Medications at Baseline 25

eTable 5. Detailed Cardiometabolic Medications at Follow up 26

eTable 6. Additional Biochemistry at Baseline. 27

eTable 7. Additional Biochemistry at Follow Up. 28

eFigure 2. Change in per-protocol maximal left atrial volume index using cardiac MRI. 29

eFigure 3. Time to first serious adverse cardiovascular event 30

© 2023 Ledwidge M et al. *JAMA Cardiology.*

eTable 8. Serious Adverse Event (SAE) over the study data by System-Organ-Class 31

© 2023 Ledwidge M et al. *JAMA Cardiology.*

eMethods 1. Trial design, Oversight, Investigational Medicinal Product and Randomisation

eTable 1. PARABLE Inclusion and Exclusion Criteria

**Inclusion Criteria**	**Exclusion Criteria**
To be eligible for inclusion, each patient was required to meet each of the following criteria at screening (Visit 1) and must continue to fulfil these criteria at baseline (Visit 2).<br><br>1. Age > 40yrs with cardiovascular risk factor(s) including at least one of:<br>a. History of systemic hypertension (medicated for greater than one month);<br>b. History of Type II diabetes;<br>2. Elevated NP: Elevated NP: BNP between 20 and 280pg/ml or NT-proBNP values between 100 pg/ml and 1,000 pg/ml within 6 months prior to screening or at screening<br>3. LAVI > 28 mL/m2 obtained during Doppler Echocardiography within 6 months prior to screening or at screening<br>4. Willing to give written informed consent to participate in the study and before any study related assessments are performed.	1. A history of heart failure.<br>2. Asymptomatic left ventricular systolic dysfunction defined as LVEF <50% on most recent Doppler-echocardiography measurement.<br>3. Systolic blood pressure <100mmHg<br>4. Persistent atrial fibrillation.<br>5. History of hypersensitivity, allergy or intolerance to sacubitril/valsartan, ARB or neprilysin therapy or to any of the excipients or other contraindication to their use.<br>6. Previous history of intolerance to recommended target doses for ARBs<br>7. Subjects who require treatment with both an ACE inhibitor and an ARB<br>8. Presence of severe, haemodynamically significant mitral and /or aortic valve disease.<br>9. Presence of hemodynamically significant obstructive lesions of left ventricular outflow tract, including aortic stenosis.<br>10. Conditions that are expected to compromise patient survival over the study period.<br>11. Serum potassium level > 5.2 mmol/L at screening.<br>12. Severe renal insufficiency (eGFR <30 mL per minute per 1.73 m^2).

© 2023 Ledwidge M et al. *JAMA Cardiology.*

	13. Hepatic dysfunction (aspartate aminotransferase or alanine aminotransferase values exceeding 3x upper limit of normal)<br>14. Concomitant use of aliskiren<br>15. History of angioedema.<br>16. History or evidence of drug or alcohol abuse within the last 12 months<br>17. Malignancy or presence of any other disease with a life expectancy of < 2 years<br>18. Women who are pregnant, breast-feeding, or women of child bearing potential not using estro-progestative oral or intra-uterine contraception or implants, or women using estro-progestative oral or intra-uterine contraception or implants but who consider stopping it during the planned duration of the study. A postmenopausal state is defined as no menses for 12 months without an alternative medical cause. (Contraception must be continued for one week following discontinuation of study drug).<br>19. Concomitant participation in other drug intervention trials<br>20. Participation in any investigational drug trial within one month of visit 1.<br>21. Refusal to provide informed consent<br>22. Subjects with contraindications to MRI<br>a) Brain aneurysm clip<br>b) Implanted neural stimulator<br>c) Implanted cardiac pacemaker or defibrillator incompatible with MRI<br>d) Cochlear implant<br>e) Ocular foreign body (e.g. metal foreign body)

© 2023 Ledwidge M et al. *JAMA Cardiology.*

	f) Other implanted medical devices: (e.g. Swan-Ganz catheter)<br>g) Insulin pump<br>h) Metal shrapnel or bullet.<br>i) Severe clasutrophobia<br>23. Any surgical or medical condition which might significantly alter the absorption, distribution, metabolism, or excretion of study drugs, including but not limited to any of the following:<br>a) History of major gastrointestinal tract surgery including gastrectomy, gastroenterostomy, or bowel resection.<br>b) Inflammatory bowel disease during the 12 months prior to Visit 1.<br>c) Any history of pancreatic injury, pancreatitis or evidence of impaired pancreatic function/injury as indicated by abnormal lipase or amylase.<br>d) Evidence of hepatic disease as determined by any one of the following: serum glutamic-oxaloacetic transaminase or serum glutamic pyruvic transaminase values exceeding 3 x upper limit of normal at Visit 1, a history of hepatic encephalopathy, a history of oesophageal varices, or a history of portocaval shunt.

1.1 Diagnosis of Type 2 diabetes

Type 2 diabetes was indicated on referral from the family physician. The criteria for diagnosis of type 2 diabetes, which are based on WHO criteria requires the following and is mandated in the current Health Services Executive agreement with family physicians (https://www.hse.ie/eng/about/who/gmscontracts/2019agreement/agreement-2019.pdf).

© 2023 Ledwidge M et al. *JAMA Cardiology.*

[1]. Symptoms of diabetes plus random plasma glucose concentration > 11.1 mmol/L (Note, random is defined as any time of day without regard to time since last meal). The classic symptoms of diabetes include polyuria, polydipsia, and unexplained weight loss.

or

[2]. Fasting plasma glucose ≥ 7.0 mmol/L. Fasting is defined as no caloric intake for at least 8 hours.

or

[3]. 2-hr plasma glucose > 11.1 mmol/L during a 75g Oral Glucose Tolerance Test. The test should be performed as described by World Health Organisation, using a glucose load containing the equivalent of 75 g anhydrous glucose dissolved in water.

or

[4].A HbA1c ≥ 48mmol/mol (≥ 6.5%)

eFigure 1. Design of the PARABLE trial

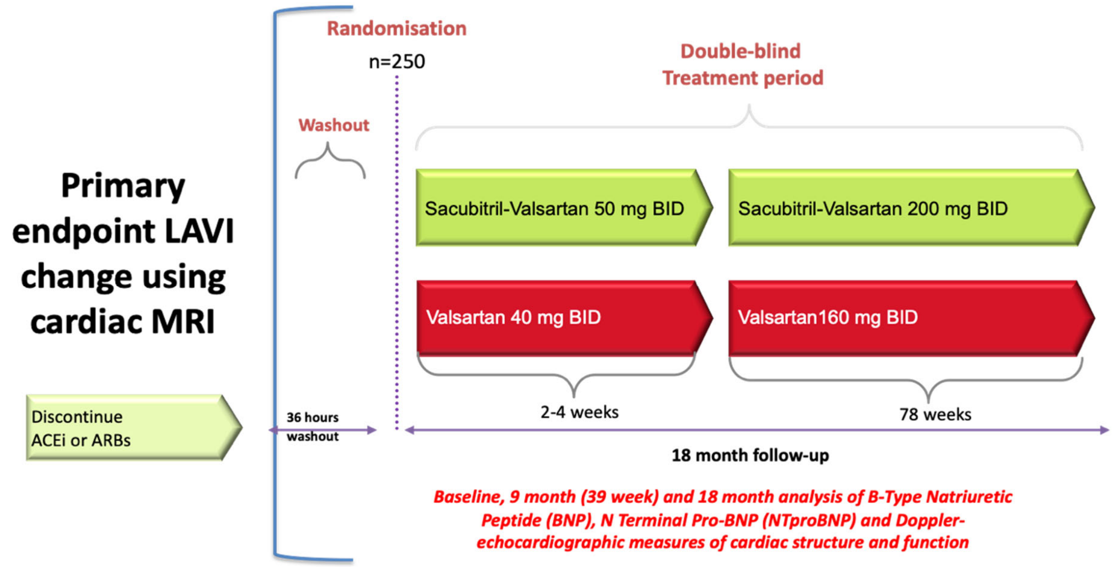

Abbreviations: LAVI, left atrial volume index; ACEi, angiotensin converting enzyme inhibitor; ARB, angiotensin receptor blocker; MRI, magnetic resonance imaging; BID, twice daily.

1.2 Trial Oversight

The Principal investigators (KMD, ML) designed and oversaw the conduct of the trial and data analysis, in collaboration with the sponsor, the Heartbeat Trust, as well as an independent data and safety monitoring committee. Data were collected, managed, and analysed by the Investigators and corroborated by an independent statistician (SZ).

© 2023 Ledwidge M et al. *JAMA Cardiology.*

All study procedures were managed by the Principal Investigators and the Sponsor. The trial was funded using a combination of grant funding from the Health Research Board of the Government of Ireland, the European Commission Framework Programme 7, the Heartbeat Trust CLG and a grant from the manufacturer of sacubitril/valsartan (Novartis). Under the terms of this grant, the study was an investigator-led, Heartbeat Trust sponsored clinical trial. Full details of the trial protocol and contractual arrangements with the funder are available online at clinicaltrials.gov (NCT04687111). Novartis had no access to the data, except for standard pharmacovigilance reporting requirements, and had no input into the data analysis or the manuscript writing. All the authors agreed to submit the manuscript for publication and vouch for the accuracy and completeness of the data and the fidelity of the trial to the protocol, the principals of Good Clinical Practice, the requirements of the General Data Protection Regulation of the European Union and requirements of the Health Research Regulations (2018) of the Government of Ireland.

Data will be made available to research collaborators on request and subject to Data Protection Agreements. This will be made for specific, novel analyses, following ethics approval and with a signed data access agreement. All requests will be made directly to the first and last authors; mark.ledwidge@ucd.ie and kenneth.mcdonald@ucd.ie.

1.3 Investigational Medicinal Product

Investigational medicinal products (sacubitril/valsartan, valsartan) were obtained free of charge from Novartis AG and were blinded along with matching dummies using over-encapsulation to ensure investigator and patient blinding in a Good Manufacturing Practice facility operated by Almac Group, Craigavon, Northern Ireland (https://www.almacgroup.com/clinical-services/).

1.4 Randomisation

Random sequence allocation, the allocation concealment mechanism and the implementation was overseen by Almac Group using WebEZE™ clinical trial management software as part of fully validated GCP compliant system offered by Almac Clinical Services, Craigavon, Northern Ireland (https://www.almacgroup.com/clinical-services/webez/). Authorised study investigators enrolled patients. The WebEZE ™ randomisation system for clinical trials (https://www.almacgroup.com/clinical-services/webez/) generated random allocation sequences to one of the two treatment arms. Assignment of clinical supplies (sacubitril/valsartan and valsartan investigational medicinal product and dummies) to patients in a double-blind, manner was maintained using WebEZE™ maintaining blinding to participants, care providers, investigators and those assessing outcome measures throughout the trial. Blinding was maintained using

© 2023 Ledwidge M et al. *JAMA Cardiology.*

investigational medication product numbers generated by WebEZE™ which were linked to the treatment group allocation and blinded to participants and study personnel. Each patient received blinded investigation medicinal product and dummy product. The link to treatment group allocation was concealed by the WebEZE™ until after the database lock and trial completion.

eMethods 2. Medication Titration Protocol

eTable 2. Study Titration Schedule

Standard dosing schedule:

Visit #	Dose level	Sacubitril/valsartan	Valsartan
1 (screening)	-	-	-
2 (Day 0 /baseline)	2	• LCZ696 100mg tablets<br>• Placebo to match Valsartan 80mg tablets	• Valsartan 80mg tablets<br>• Placebo to match LCZ696 100mg tablets
3-11 (Day 14-EOS)	3	• LCZ696 200mg tablets<br>• Placebo to match Valsartan 160mg tablets	• Valsartan 160mg tablets<br>• Placebo to match LCZ696 200mg tablets

Lower starting dose schedule

Visit #	Dose level	LCZ696	Valsartan
1 (screening)	-	-	-
2 (Day 0 / baseline)	1	• LCZ696 50mg tablets<br>• Placebo to match Valsartan 40mg tablets	• Valsartan 40mg tablets<br>• Placebo to match LCZ696 50mg tablets
3 (Day 14)	2	• LCZ696 100mg tablets<br>• Placebo to match Valsartan 80mg tablets	• Valsartan 80mg tablets<br>• Placebo to match LCZ696 100mg tablets
4-11 (Day 28-EOS)	3	• LCZ696 200mg tablets<br>• Placebo to match Valsartan 160mg tablets	• Valsartan 160mg tablets<br>• Placebo to match LCZ696 200mg tablets

2.1 Overview of Titration

© 2023 Ledwidge M et al. *JAMA Cardiology.*

A starting dose of sacubitril/valsartan 50mg twice daily or valsartan 40mg twice daily was used for subjects not currently taking an ACE inhibitor or an ARB, and/or for subjects previously taking low doses of these agents (as per Investigator judgement). A lower dosing regimen was also used for subjects with a systolic BP of ≥100mm and ≤110mmHg at screening or baseline. The dose of sacubitril/valsartan and valsartan was doubled every 2 to four weeks to the target dose of 200mg and 160mg twice daily for sacubitril/valsartan and valsartan respectively, as tolerated by the subject. All subjects were reviewed two weeks after dose titration for tolerability issues (e.g. symptomatic hypotension, hyperkalaemia, renal dysfunction). In cases of poor tolerability, temporary down-titration of study drug, co-medication or discontinuation was considered.
Every attempt was made to maintain subjects on the target dose level (i.e. sacubitril/valsartan 200mg and Valsartan 160mg) for as long a duration as possible throughout the trial. If, however, in the opinion of the Investigator, the subject did not tolerate the target dose of study drug (Phase 3), the Investigator considered whether non-disease-modifying medication (e.g., calcium channel blockers, diuretics, nitrates, alpha blockers) could be reduced, before considering reducing the dose of the study drug to the next lower dose level.

If down titration was necessary, the subject was down-titrated to the next lower dose level. The subject may have continued to receive the lower dose level for a recommended period of 1 to 4 weeks before re-challenging the subject with the next higher dose level. If the tolerability issues were not alleviated despite down-titration by one dose level, the Investigator could lower the study drug dose further to the next lower level for 1 to 4 weeks, up to temporary withdrawal of the study drug. Again, once stable, the subject was rechallenged with study drug or up-titration to the next higher dose level every 1 to 4 weeks in an attempt to bring back the subject gradually to the target study drug dose level (dose level 3). The Investigator was free to choose the next dose level for down- or up-titration according to his or her clinical judgment. Dose level 1 or 2 was maintained if the Investigator considered that the subject's condition would not allow any further up-titration to the target dose of study medication (level 3).

If needed, the study drug was stopped completely, but the subject was encouraged to continue to attend the study visits until the completion of the study. Study drug dose level adjustments were mainly based on overall safety and tolerability with special focus on a) hyperkalaemia; b) symptomatic hypotension; and c) clinically significant decrease in eGFR/increase in serum creatinine All dosage adjustments were recorded in the subjects CRF.

© 2023 Ledwidge M et al. *JAMA Cardiology.*

The use of an ACE inhibitor and ARB concomitantly with the study drug after randomisation was strictly prohibited and the Investigator completed a ‘Study Wallet Card’ for each subject. Patients were advised to carry the card with them at all times and to present the card to medical and pharmacy personnel should they require any medical care outside the study during the treatment phase of the study.

2.2 Treatment guidelines for hyperkalaemia (serum K+ > 5.2 mEq/L)

General principles: It was recommended that any subject with a serum K^+ > 5.2 mEq/L following randomization required frequent checks of potassium concentration until it was clear that the potassium concentration was stable and not rising into the range of concern (> 5.5 and < 6.0 mEq/L) or potential danger (> 6.0 mEq/L). Investigators did not randomize a subject with a serum K^+ > 5.2 mEq/L at Visit 1 (screening) and followed the other measures outlined below if K^+ increased to values > 5.2 mEq/L after randomization.

Corrective action recommended for management of hyperkalaemia:
[1] K^+ > 5.2 and ≤ 5.5 mEq/L

- If necessary, confirm potassium concentration was obtained in a non-haemolysed sampled. If it was confirmed that questionable results were obtained from haemolysed specimen, a redraw blood sample for K^+ measurement and caution was taken to minimize the incidence of haemolysis by proper blood sample collection and handling
- A low potassium diet and restriction of food/drinks with high potassium content (e.g. orange juice, melon, bananas, low-salt substitutes, etc.) was reinforced.
- The patient’s medical regimen (including dietary supplements and over-the-counter medications) was reviewed for agents known to cause hyperkalaemia. A reduction in dose or discontinuation of the following agents was considered:
    - Confirmation was performed that potassium-sparing diuretics and potassium supplements were not being used
    - Salt substitutes
    - Non-steroidal anti-inflammatory drugs (NSAIDs)
    - Cyclo-oxygenase-2 (COX-2) inhibitors
    - Trimethoprim and trimethoprim-containing combination products such as trimethoprim/sulfamethoxazole

© 2023 Ledwidge M et al. *JAMA Cardiology.*

- Herbal Supplements: (for example, Noni juice, alfalfa (Medicago sativa), dandelion (Taraxacum officinale), horsetail (Equisetum arvense), nettle (Urtica dioica), milkweed, lily of the valley, Siberian ginseng, hawthorn berries)

[2] $K^+ > 5.5$ and ≤ 6.0 mEq/L

- If necessary, potassium concentration was obtained in a non-hemolyzed sample
- All measures outlined for K^+ 5.2 and ≤ 5.5 mEq/L
- Repeat K^+ measurement after 1-3 days

  The study drug was discontinued if a repeated $K^+ > 5.5$ mEq/L

[3] $K^+ > 6.0$ mEq/L

- Immediate discontinuation of the study drug
- Potassium concentration confirmed in a non-haemolysed sample
- Urgently evaluation of subject and treatment for hyperkalaemia as clinically indicated
- All measures outlined for $K^+ > 5.2$ and ≤ 6.0 mEq/L also applied

2.1 <u>Treatment guidelines for the management of hypotension</u>

1. Treatment was not initiated if systolic BP <100mmHg at screening or baseline. A lower starting dose (Level 1) was considered for patients with systolic BP ≥100 to 110mmHg.
2. Blood pressure was monitored closely throughout the study
3. If systolic BP fell to ≤95mmHg (or symptomatic hypotension occurs at any BP):

- Correction of any treatable cause, e.g. hypovolemia
- If symptomatic hypotension persisted, consideration was given to down titrating or stopping other hypotensive medications, and/or study drug as appropriate.
- If symptomatic hypotension persisted during two consecutive office visits or as judged by the Investigator, the study drug was discontinued.

2.3 Treatment guidelines for the management of renal dysfunction

<u>General principles:</u> Subjects that developed renal dysfunction after study drug administration were treated in two ways:

Surveillance conditions

© 2023 Ledwidge M et al. *JAMA Cardiology.*

If, at any moment after recruitment, serum creatinine increased by ≥50% from baseline (Visit 1), the Investigator checked for potentially reversible cases of renal dysfunction such as:

- NSAID intake, diuretics, antibiotics, or other treatments known to affect creatinine
- Volume decrease
- Urinary infection
- Urinary tract obstruction
- Study medication

The Investigator considered discontinuing any medication known to affect renal function and repeated the serum creatinine after 5-7 days.

Action conditions

If serum creatinine >3.0 mg/dL or increased by ≥100% from baseline (Visit 1), the Investigator discontinued the study drug and checked for potentially reversible causes of renal dysfunction (see above). Thereafter, serum creatinine was monitored until it normalized.

2.4 Treatment guidelines for study drug restart after temporary treatment interruption

Study drug was reintroduced where possible in those who temporarily discontinued it as soon as medically justified in the opinion of the Investigator and at the most appropriate and allowable dose level per his/her medical judgment. If the subject did not tolerate the re-start study drug dose level, the study drug was down-titrated again (if appropriate) or discontinued again and a new attempt to up-titrate or reintroduce the study drug was considered by the Investigator as soon as medically justified in his/her medical judgment.

Additional medications or interventions were administered at the discretion of the attending physician. The use of an ACE inhibitor an ARB concomitantly with the study drug after randomisation was strictly prohibited.

The Investigator completed a ‘Wallet Card’ for each patient. Patients were advised to carry the card with them at all times and to present the card to medical personnel if they required any medical care outside the study while participating in the study.

© 2023 Ledwidge M et al. *JAMA Cardiology.*

eMethods 3 – Description of Study Assessments

3.1 Laboratory parameters

Peripheral venous blood samples were drawn at the timelines shown in Table 2. Laboratory examinations were analysed in St Michael's Hospital laboratory or St Vincent's Hospital laboratory. Methods for sampling and handling are detailed in the PARABLE laboratory manual (HBT-GCP-SOP-16 'Handling of Laboratory Samples in the PARABLE Study'). Evaluations included the following:

- Biochemistry
    - Urea & Electrolytes (U&E)
    - Liver Function Tests (LFTs)
    - Alkaline Phosphatase
    - Lipid profile
    - HbA1c
    - Iron studies
- Haematology
    - Full blood count

Sample processing and analysis was done as per local hospital laboratory standard operating procedures. The local hospital laboratory provided the report to the Investigator responsible for the clinical assessment of the results. Any abnormality was assessed for its clinical significance. Any clinically significant value was recorded as an adverse event (AE) in the clinical report form (CRF) and followed up.

3.2 Estimated Glomerular Filtration Rate assessments

The estimated glomerular filtration rate (eGFR) to determine eligibility of the subject for screening into the trial was calculated at Visit 1 (screening) from the serum creatinine concentration. The eGFR was further measured at Visit 8 (month 9) and Visit 11 (EOS). eGFR was calculated using the following formula: eGFR (mL/min/1.73 m^2) = 175 × (standardized SCr in mg/dL)-1.154 × (age in years)-0.203 × (0.742 if female) × (1.212 if black), where SCr was the standardized serum creatinine value.

© 2023 Ledwidge M et al. *JAMA Cardiology.*

eTable 3. Overview of Study Assessments.

Visit number	1	2	3	4	5	6	7	8	9	10	11	12
Assessment	Wk-2 Screening	D0 Baseline	Wk 2	Wk 4	Wk 6	Wk 13 / Mth 3	Wk 26 / Mth 6	Wk 39 / / Mth 9	Wk 52 / Mth 12	Wk 65 / Mth 15	Wk 78 / Mth 18 (EOS)	Mth 27 (Optional sub-study)
			± 3d	± 3d	± 3d	± 7d	± 7d	± 7d	± 7d	± 7d	± 7d	± 28
Assessment of eligibility	x											x
Pregnancy test (WOCBP)	x	x	x	x	x	x	x	x	x	x	x	x
Informed consent	x											x
Randomisation		x										
Medical history	x											
Physical exam	x	x	x	x	x	x	x	x	x	x	x	x
Height (H) and weight (W)		H/W						W			W	H/W
Waist/hip circumference		x						x			x	x
Vital signs (BP and pulse)	x	x	x	x	x	x	x	x	x	x	x	x
Natriuretic peptide	x[#]	x	x	x	x	x	x	x	x	x	x	x
Biomarkers		x			x	x	x	x	x	x	x	x
U&E, LFT, Alk phos	x (no alk phos)	x	x	x	x	x	x	x	x	x	x	x
Haematology	x	x						x			x	x
eGFR	x	x						x			x	x
HbA1c, lipid profile, iron studies		x						x			x	x
cGMP (urinary)		x			x	x		x			x	x
ABPM		x						x			x	x
Electrocardiography (ECG)		x						x			x	x
Doppler echocardiography	X[¥]	x[†]						x			x	x
Cardiac MRI**		x									x	
Questionnaires***		x						x			x	x
Concomitant medications	x	x	x	x	x	x	x	x	x	x	x	
Adverse events			x	x	x	x	x	x	x	x	x	x
Dispense medication		x	x	x	x	x	x	x	x	x		
Drug accountability			x	x	x	x	x	x	x	x	x	
Holter monitor (optional)											x	

© 2023 Ledwidge M et al. *JAMA Cardiology.*

Abbreviations: ABPM, ambulatory blood pressure monitoring; Alk Phos, alkaline phosphatase; BP, blood pressure; cGMP, cyclic guanosine monophosphate; ECG, electrocardiogram; eGFR, estimated glomerular filtration rate; EOS, end of study; HbA1c, glycated haemoglobin; LFT, liver function tests; U&E, urea and electrolytes; WOCBP, women of child bearing potential.

*Source: results of these assessments were filed in the Subject Study File on the original source document (questionnaires) or a copy of the source document (e.g. laboratory report, Doppler Echo report, ECG (this must be a copy of the original), ABPM printout etc.)

¥ Echo was possibly needed at Visit 1 if one hasn't been performed in the previous six months.

† If an Echo was performed at screening, it was not repeated at baseline, unless the investigator decided otherwise.

** If the patient had a cardiac MRI in the previous six weeks, then it was not repeated at baseline

*** The Mini Mental State Exam and Montreal Cognitive Assessment questionnaires will also be administered five years after the patient's end of study visit.

Natriuretic peptide levels were needed at Visit 1 if one hadn't been performed in the previous six months

© 2023 Ledwidge M et al. *JAMA Cardiology.*

3.3 Physical exam

A physical exam included the examination of general appearance and a cardiac exam at every visit. Information was recorded in the CRF. Important findings present prior to the start of study drug were included in the relevant section of the subject's CRF. Significant findings made after the start of study drug which met the definition of an AE were recorded on an AE form in the subject's CRF.

3.4 Vital signs

Vital signs were assessed at every visit. This included blood pressure (BP) and pulse measurements. BP was measured by using a standard sphygmomanometer with an appropriate size cuff and the non-dominant arm in the sitting position after 5 minutes of rest. This BP (and not 24h ABPM) was used to confirm eligibility and inform dosing decisions.

3.5 Height, weight and waist/hip circumference

Height in centimetres (cm) was measured at Visit 2 (baseline). Body weight (to the nearest 0.1 kilogram [kg] in indoor clothing without shoes) and waist/hip circumference (at natural waistline located above the belly button and below the rib cage; started at one hip and wrapped around the rear, making sure the tape was over the largest part of the buttocks) (to the nearest centimetre [cm] in indoor clothing) was measured at Visit 2 (baseline), Visit 8 (month 9) and Visit 11 (EOS).

3.6 Electrocardiography

A standard 12-lead electrocardiograph (ECG) (after 5 minutes rest) was performed (at Visit 2 (baseline), Visit 8 (month 9) and Visit 11 (EOS)) using appropriate procedures (i.e. paper speed 25 mm/sec, amplitude 10 mm/mV, at least 10 seconds recording available for measurement) and the use of an automatic recorder with the capacity for digital signal processing. The heart rate was measured, and relevant abnormalities regarding cardiac rhythm, conduction and repolarisation parameters such as QT prolongation or atrio-ventricular block, were recorded. A copy of the print-out was kept in the subject's study file. The results were assessed within the CRF as within normal limits, abnormal but not clinically significant or abnormal and clinically significant.

3.7 Ambulatory blood pressure monitoring (ABPM)

ABPM was measured at Visit 2 (baseline), Visit 8 (month 9) and Visit 11 (EOS).

The device employed for ABPM analysis was the SpaceLab 90207 oscillometric device. Depending on arm circumference, two standard cuff sizes were used with application to the non-dominant arm. Subjects were instructed to go about their usual activities with minimal restrictions. The systolic

© 2023 Ledwidge M et al. *JAMA Cardiology.*

blood pressure (BP), diastolic BP, and heart rate of each subject was automatically measured every 30 minutes for 24 consecutive hours with a properly calibrated SpaceLab 90207 device. The device was fitted and removed by a cardiovascular technician/nurse blinded to study treatment allocation. If quality criteria were not met, one repeat measurement was allowed during the following 1–2 month period. Nocturnal dipping was defined as subjects with a more than 10% fall in nocturnal BP relative to daytime values. Non-dipping was defined as a less than 10% nocturnal fall in BP.

3.8 Serum BNP and NT-proBNP

NP measurements required for eligibility were based on levels measured within the last 6 months or at screening. All serum BNP assessments from the peripheral circulation were measured using the Alere Triage® BNP test, a POC fluorescence immunoassay. Several drops of Ethylenediaminetetraacetic acid (EDTA) anticoagulated whole blood were added to the sample port of the test device which was then inserted into the Triage meter. The Alere Triage ® Point-of-care BNP assay had a sensitivity limit ≤ 5 pg/mL (Alere Inc.). Although the Alere Triage ® BNP assay used in our study was not specific for active BNP 1-32, which is virtually undetectable in peripheral blood, it cross-reacts with proteolyzed BNP fragments (e.g. BNP3-32) and proBNP, especially the glycosylated form, which, in turn, are elevated in left ventricular dysfunction and heart failure. We obtained BNP levels from patients at point of care at baseline and follow up Doppler-echocardiography.

Serum NT-proBNP from the peripheral circulation was measured from EDTA plasma samples from patients at point of care at baseline and follow up Doppler-echocardiography. Samples were frozen at -80°C and were thawed for measurements using assays conducted according to the manufacturer's instructions on an ARCHITECT i2000SR analyzer (Abbott Diagnostics, Abbott Park, IL). NT-proBNP was measured using the Alere NT-proBNP for ARCHITECT chemiluminescent microparticle immunoassay with maximum coefficient of variation of 20% across a range of 8.2 to 35,000.0 pg/mL and a reported limit of detection (LoD) of 4.9 pg/mL (Manufacturers package insert, revised October 2017).

3.9 Echo-Doppler studies

Echocardiography was performed at Visit 2 (baseline), 8 (month 9) and 11 (EOS) to assess myocardial function. This was performed by a designated experienced echocardiographer who was blinded to treatment assignment. Left atrial volume indexed (LAVI) measurements required for eligibility (LAVI >28 mL/m^2) were based on echocardiograms performed within the last 6 months. If a LAVI measurement from the last six months was not available, an additional echocardiography was performed at the screening visit. If an echo was performed at the screening visit, this was used for

© 2023 Ledwidge M et al. *JAMA Cardiology.*

the baseline visit (unless using clinical judgement, the investigator decided that another echo was necessary). A baseline echo had to be performed within 90 days prior to baseline visit.

The following parameters were reported:

- Left ventricular ejection fraction
- LAVI
- E/A ratio
- E wave deceleration time
- E/E’
- Isovolumetric relaxation time
- Pulmonary venous flow analysis
- Tissue Doppler studies

Standard two-dimensional, targeted M-Mode and Doppler Echocardiographic measurements were obtained with the subject lying in the left lateral position. M-mode measurements were taken according to the guidelines from the American Society of Echocardiography. Left ventricular ejection fraction was calculated using the Simpson’s biplane method. The following pulsed Doppler measurements from 3 consecutive cardiac cycles were obtained in the apical view with a cursor at mitral valve inflow: maximal early (E) and late (A) transmitral velocities in diastole and E wave deceleration time. Isovolumetric relaxation time were measured in the apical four chamber view by continuous wave Doppler placed between the mitral inflow area and left ventricular outflow tract. Left ventricular filling pressures were noninvasively assessed through analysis of the ratio of peak E-wave (E) to maximum velocity of lateral mitral valve annulus (E’) ratio (E’/E) measured at the lateral mitral valve annulus. An experienced sonographer blinded to treatment allocation carried out all echocardiography imaging and reporting.

Tissue Doppler imaging was used to record longitudinal velocities of the mitral anulus. The velocities were recorded from the apical four-chamber view by placing a 2-5mm sample volume of the lateral portion of the mitral anulus. The E' velocity was recorded and the ratio between E and E' (E/E') calculated.

In assessing changes over 18 months of left ventricular structure and function as part of the secondary objectives of the study, a E/E'> 15 was taken as evidence of diastolic dysfunction. An E/E' < 8 excluded the diagnosis. An E/E' ratio ranging from 8 to 15 was considered suggestive but non-diagnostic evidence of left ventricular diastolic dysfunction and needed other imaging evidence of

© 2023 Ledwidge M et al. *JAMA Cardiology.*

diastolic dysfunction. These consisted of blood flow Doppler evaluation of mitral valve or pulmonary veins, echo measures of LV mass index or LAVI or electrocardiographic evidence of atrial fibrillation.

3.10 Cardiac magnetic resonance imaging

Cardiac MRI was performed on 1.5 T scanners (Avanto, Siemens Healthcare, Forcheim, Germany or Signa HDX, General Electric Healthcare, Milwaukee, Wis, USA); All images were acquired using an 8-channel phased array surface coil during mild expiration and electrocardiographic vector-gated triggering. Cine images was acquired in the 4-, 3-, 2-chamber and contiguous short axis views using a balanced steady state free precession (SSFP) sequence. The short axis sequence covered the entire left atrium and left ventricle. Cine parameters were repetition time, 3.5 ms; echo time, 1.4 ms; matrix,192x192; field of view, 28x34 cm; flip angle, 60-70 degrees; slice thickness 6-8 mm, slice gap 1mm.

Cardiac MRI Image Analysis

Image analysis was performed on cardiac MRI specialized software (Syngo Via, Siemens Healthcare Erlangen, Germany). Quantitative image analysis was performed by two cardiothoracic imaging fellowship trained readers with > 5 years attending experience (JD, DM). For the purposes of the measurement of the primary and secondary MRI endpoints, measurements were performed using standard techniques according to the Society for Cardiovascular Magnetic Resonance guidelines for reporting cardiovascular magnetic resonance examinations [Schulz-Menger et al. Journal of Cardiovascular Magnetic Resonance (2020) 22:19 https://doi.org/10.1186/s12968-020-00610-6).

As mentioned above, all study personnel and observers were blinded to randomization arm and patient details. For LV analysis, contours of end-diastolic and -systolic endocardial as well as epicardial borders were semiquantitatively marked on the stack of short-axis images. Papillary muscles were excluded when measuring LV myocardial mass (LVMM) and included when measuring LV volumes. Basal slices were included if at least half of the LV circumference included the myocardium. Inclusion or exclusion of apical slices depended on visibility of the myocardium. Values of LV stroke volume (LVSV) were calculated as the difference between end-diastolic (LVEDV) and end-systolic volumes (LVESV). The LV ejection fraction (LVEF) was computed as LVSV/LVEDV x 100. LVMM was calculated at end diastole using 1.05 g/cm^3 as the specific density of the myocardium. LV volumes and LVMM indexed to the body surface area (BSA) were calculated using the Du Bois formula: BSA = 0.007184 x (height in cm)$^{0.725}$ x (weight in kg)$^{0.425}$. Left atrial volume index was measured using the short axis slices of the entire left atrium. The left atrial border was manually

traced in left atrial end-systole and end-diastole for each slice. The border were defined by the left atrial wall, the mitral annular plane and the ostia of the pulmonary veins. The left atrial appendage and the pulmonary veins were not included in the analysis. Maximum LA volume was measured in the frame just before mitral valve opening. The minimum LA volume was measured in the frame just after the P wave at mitral valve closure. The LA diastolic and systolic volumes were automatically calculated by the software and indexed manually to BSA. Furthermore, the LA ejection fraction was automatically calculated by the software using the formula: [(LA diastolic volume - LA systolic volume)/LA diastolic volume] x 100. Left atrial and left ventricular volumes were calculated using summation of area (slice thickness + interslice gap) for each slice (Simpson's method).

In *post-hoc* analyses, arterial elastance index was calculated according to the methodology of Wohlfart et al. to provide a measure of total arterial load, incorporating mean and pulsatile components.[35] This was calculated as end systolic pressure divided by stroke volume index (0.9 x SBP/SVi). Other measures of arterial elastance were calculated (SBP/SVi and Pulse pressure / SVi). Systemic Vascular Resistance (SVR) was calculated as =mean BP x 80/cardiac output and Total Arterial Compliance (TAC) as =SV/pulse pressure. The ratio of echo-Doppler and tissue-Doppler early diastolic velocities (E/e') was used to estimate LV end diastolic pressure (LVPd) (=11.96 + 0.596 x E/e'). Left ventricular end diastolic chamber stiffness index (LVSId) was estimated by the ratio of LVEDP and LV end diastolic volume, indexed to body surface area. Left atrial end diastolic chamber stiffness index (LVSId) was estimated by the ratio of LVEDP and LAVi max. The LV end diastolic volume standardised to a filling pressure of 30mmHg (EDV30) was calculated in accordance with the methods of Klotz, Burkhoff et al. (https://pubmed.ncbi.nlm.nih.gov/16428349/ and https://www.nature.com/articles/nprot.2007.270). All post-hoc outcome measures were calculated using results of volumetric cardiac MRI where possible.

© 2023 Ledwidge M et al. *JAMA Cardiology.*

eMethods 4. Detailed Description of Sample Size Calculations and Statistical Methods

Based on an analysis of a cohort within the STOP-HF follow up programme with elevated LAVI >28 mL/ m^2, EF>50% and BNP >20pg/mL, we anticipated a baseline mean LAVI of 36.2 ± 6.5 mL/m^2. This is similar to the baseline level observed in the PARAMOUNT study,[18] wherein a 36-week treatment period with sacubitril/valsartan was associated with a difference in LAVI of 2.6 mL/m^2. The expected effect size was assumed to be 2.6/6.5 = 0.38 and using an alpha value of 5% (two-tailed), beta = 20% and an independent t-test to assess the change in LAVI over time, the required number of subjects to detect this effect was 222 (111 per study arm), or 248 after accounting for a 10% dropout rate. Accordingly, 250 patients were recruited to the study. In a review of the expected effect size, once blinded echocardiography was available for 125 patients at the 9-month time point, it was noted that the actual standard deviation of baseline to 9-month change scores was 4.7 mL/m^2. This resulted in a change of expected effect size to at least (2.0/5.0) = 0.40. An effect size of 0.4 would require 96 patients in each group, suggesting that, based on the assumptions above, the study was adequately powered to detect changes in LAVi on echocardiography. In addition, the primary endpoint was measured more precisely using cardiac MRI. The study also had at least 80% power with a two-tailed alpha = 5% to detect a 6 g/m^2 difference in LVMI change by cardiac MRI and a 2-unit difference in E/e' or e' using tissue doppler measurements.

Demographics and clinical characteristics were summarised with descriptive statistics; continuous variables are presented as mean (SD) or median with interquartile ranges and binary variables are presented as *n* (%). Statistical tests for differences on continuous variables between groups were Student *t*-test or Wilcoxon signed–rank test, depending on whether the variables were normally distributed. The Shapiro–Wilk test was used to test whether variables could be considered normally distributed with alpha set at 0.05. Normal distribution for BNP and NT-proBNP was approximated by taking logarithm of positively skewed BNP and NT-proBNP for analysis. Statistical tests for differences on binary variables between groups were the Chi-squared test or Fisher's exact test, depending on whether the *n* in any cell in the 2 × 2 summary table was less than 5. Missing data were addressed using multiple imputation using chained equations (using R software's "mice" package) with 10 imputations and 10 iterations per imputation and results were presented (primary and secondary end points) as pooled results from the multiple imputed data sets. Repeated changes (e.g. ABPM, NTproBNP, Doppler-echocardiography) from baseline to 18 months were analysed using ANOVA with repeated measures mixed models.

© 2023 Ledwidge M et al. *JAMA Cardiology.*

4.1 Covid-19 Procedures

A significant proportion of the PARABLE trial was conducted during the Covid-19 pandemic, affecting 48 patients following the first lockdown in Ireland on 14th March 2020. In accordance with European Medicines Agency and Federal Drugs Administration guidance (https://www.fda.gov/media/136238/download, (https://ec.europa.eu/health/ sites/default/files/files/eudralex/ vol-10/guidanceclinicaltrials_covid19_en.pdf), the Sponsor and Principal Investigators took decisions to allow the trial to continue based on benefit-risk considerations, on contingency provisions taken nationally and locally within the hospital, and priority given to the impact on the health and safety of the trial participants. Furthermore, local and national guidance required the majority of PARABLE patients to "cocoon" during this period. Patients were reluctant to attend the hospital in person, preferring to have care administered remotely. This, however, resulted in protocol deviations, difficulties accessing diagnostics in a timely manner and patient withdrawals. Before the completion of the trial, data lock and unblinding of the final dataset, we pre-specified a per-protocol analysis of the primary endpoint and analysis of the MACE endpoint prior to the first lockdown in Ireland (March 14th 2020).

© 2023 Ledwidge M et al. *JAMA Cardiology.*

eTable 4. Detailed Cardiometabolic Medications at Baseline

	Sacubitril/valsartan N=122	Valsartan N=128	N
	A N=122	B N=128	N
Aliskiren, n (%)	0 (100%)	0 (100%)	250
Digoxin, n (%)	0 (0.00%)	1 (0.78%)	250
Ivabradine, n (%)	1 (0.82%)	3 (2.34%)	250
Anti-arrythmic, n (%)	6 (4.92%)	5 (3.91%)	250
Alpha-blocker, n (%)	21 (17.2%)	25 (19.5%)	250
Beta-blocker, n (%)	70 (57.4%)	69 (53.9%)	250
CCB, n (%)L	49 (40.2%)	61 (47.7%)	250
Statin, n (%)	98 (80.3%)	102 (79.7%)	250
Other Dyslipidaemic, n (%)	5 (4.10%)	9 (7.03%)	250
Thiazide Diuretic, n (%)	33 (27.0%)	41 (32.0%)	250
Aldosterone Antagonist, n (%)	12 (9.84%)	7 (5.47%)	250
Loop Diuretic, n (%)	5 (4.10%)	8 (6.25%)	250
Aspirin, n (%)	74 (60.7%)	86 (67.2%)	250
Other Antiplatelet, n (%)	8 (6.56%)	11 (8.59%)	250
DOAC, n (%)	11 (9.02%)	8 (6.25%)	250
Warfarin, n (%)	3 (2.46%)	0 (0.00%)	250
Insulin, n (%)	6 (4.92%)	5 (3.91%)	250
Oral Antidiabetic, n (%)	28 (23.0%)	23 (18.0%)	250
Metformin, n (%)	25 (20.5%)	20 (15.6%)	250
Sulphonylurea, n (%)	7 (5.74%)	7 (5.47%)	250
DPP4 inhibitor, n (%)	4 (3.28%)	5 (3.91%)	250
Gliptin, n (%)	0 (0.00%)	1 (0.78%)	250
SGLT2 Inhibitor, n (%)	2 (1.64%)	1 (0.78%)	250
Pioglitizone, n (%)	1 (0.82%)	0 (0.00%)	250

Abbreviations: CCB, calcium channel blocker; DOAC, direct acting oral anticoagulant; DPP4, Dipeptidyl-peptidase 4; SGLT2, Sodium-glucose transport protein 2.

© 2023 Ledwidge M et al. *JAMA Cardiology.*

eTable 5. Detailed Cardiometabolic Medications at Follow up

	**Sacubitril/valsartan N=117**	**Valsartan N=122**	*P* Value	N
Aliskiren, n (%)	0 (0%)	0 (0%)	.	239
Digoxin, n (%)	0 (0.00%)	1 (0.82%)	1.000	239
Ivabradine, n (%)	1 (0.85%)	3 (2.46%)	0.622	239
Anti-arrythmic, n (%)	6 (5.13%)	10 (8.20%)	0.490	239
Alpha-blocker, n (%)	17 (14.5%)	19 (15.6%)	0.964	239
Beta-blocker, n (%)	59 (50.4%)	55 (45.1%)	0.485	239
CCB, n (%)L	40 (34.2%)	46 (37.7%)	0.666	239
Statin, n (%)	83 (70.9%)	83 (68.0%)	0.728	239
Other Dyslipidaemic, n (%)	6 (5.13%)	11 (9.02%)	0.359	239
Thiazide Diuretic, n (%)	10 (8.55%)	28 (23.0%)	0.004	239
Aldosterone Antagonist, n (%)	5 (4.27%)	6 (4.92%)	1.000	239
Loop Diuretic, n (%)	2 (1.71%)	5 (4.10%)	0.447	239
Aspirin, n (%)	60 (51.3%)	64 (52.5%)	0.958	239
Other Antiplatelet, n (%)	4 (3.42%)	8 (6.56%)	0.415	239
DOAC, n (%)	13 (11.1%)	13 (10.7%)	1.000	239
Warfarin, n (%)	1 (0.85%)	0 (0.00%)	0.490	239
Insulin, n (%)	4 (3.42%)	5 (4.10%)	1.000	239
Oral Antidiabetic, n (%)	22 (18.8%)	20 (16.4%)	0.749	239
Metformin, n (%)	22 (18.8%)	22 (18.0%)	1.000	239
Sulphonylurea, n (%)	8 (6.84%)	8 (6.56%)	1.000	239
DPP4 inhibitor, n (%)	6 (5.13%)	5 (4.10%)	0.943	239
Gliptin, n (%)	1 (0.85%)	1 (0.82%)	1.000	239
SGLT2 Inhibitor, n (%)	2 (1.71%)	2 (1.64%)	1.000	239
Pioglitizone, n (%)	1 (0.85%)	0 (0.00%)	0.490	239

By the end of the titration phase (week 6), the mean dose of sacubitril/valsartan tolerated was 100/92mg ± 15/14mg BID, with 106 (88.5%) achieving target dose (103/97mg BID). The mean dose of valsartan was 153 mg ± 23 mg BID with 106 (82.8%) achieving target dose (160mg BID). At the end of the study, the mean dose of sacubitril/valsartan tolerated was 100/92mg ± 16/15mg, and the mean dose of valsartan was 154 mg ± 23 mg.

Abbreviations: CCB, calcium channel blocker; DOAC, direct acting oral anticoagulant; DPP4, Dipeptidyl-peptidase 4; SGLT2, Sodium-glucose transport protein 2.

© 2023 Ledwidge M et al. *JAMA Cardiology.*

eTable 6. Additional Biochemistry at Baseline.

	Sacubitril/valsartan N=122	Valsartan N=128	N
Sodium, mmol/L	138 [137;140]	138 [136;140]	248
Potassium, mmol/L	4.20 [4.00;4.40]	4.20 [3.90;4.40]	248
Chloride, mmol/L	103 [100;105]	103 [101;105]	248
Urea, mmol/L	6.70 [5.90;8.00]	7.05 [6.00;8.50]	248
Creatinine, μmol/L	76.0 [65.0;94.2]	79.5 [65.0;93.2]	248
eGFR, mL/min	78.0 [64.0;90.0]	77.0 [65.8;90.0]	248
Albumin, g/L	42.0 [40.0;44.0]	42.0 [40.0;44.0]	247
Bilirubin, mg/L	13.0 [11.0;16.0]	13.0 [11.0;17.0]	247
Alkaline phosphatase, u/L	66.0 [54.0;76.0]	63.0 [52.0;75.2]	247
GGT, u/L	22.0 [16.0;30.5]	21.0 [16.0;33.0]	247
ALT, u/L	22.0 [18.0;28.0]	21.0 [18.0;29.0]	247
AST, u/L	24.0 [21.0;28.0]	24.0 [22.0;29.2]	244
Haemoglobin, g/dL	13.4 [12.5;14.4]	13.5 [12.5;14.4]	247
Ferritin, μg/L	104 [58.5;142]	91.0 [45.5;144]	241

Abbreviations: ALT, alanine aminotransferase; AST, aspartate aminotransferase;eGFR, estimated glomerular filtration rate; GGT, gamma glutamyl transferase.

© 2023 Ledwidge M et al. *JAMA Cardiology.*

eTable 7. Additional Biochemistry at Follow Up.

	**Sacubitril/valsartan N=116**	**Valsartan N=120**	*P* Value	N
Sodium, mmol/L	138 [137;140]	138 [137;140]	*0.740*	236
Potassium, mmol/L	4.20 [4.00;4.40]	4.20 [3.90;4.50]	*0.767*	236
Chloride, mmol/L	104 [103;106]	104 [101;105]	*0.154*	236
Urea, mmol/L	6.85 [5.70;8.72]	7.50 [6.30;9.33]	*0.050*	236
Creatinine, μmol/L	78.0 [66.0;95.2]	85.5 [68.0;98.0]	*0.235*	236
eGFR, mL/min	77.0 [61.0;90.0]	70.0 [58.0;86.0]	*0.114*	236
Change in eGFR, mL/min	0.00 [-7.00;4.00]	-3.00 [-13.00;2.00]	*0.013*	234
HBA1c	40.0 [37.0;44.0]	40.0 [37.5;43.5]	*0.909*	233
Albumin, g/L	41.0 [39.0;42.0]	41.0 [39.0;43.0]	*0.401*	235
Bilirubin, mg/L	13.0 [10.0;16.0]	13.0 [10.0;16.0]	*0.809*	235
Alkaline phosphatase, u/L	62.5 [52.0;76.0]	62.0 [52.0;78.5]	*0.735*	235
GGT, u/L	21.0 [16.0;29.0]	21.0 [16.0;28.0]	*0.942*	235
ALT, u/L	21.0 [16.0;25.0]	22.0 [18.0;27.0]	*0.132*	235
AST, u/L	22.0 [19.0;26.0]	24.0 [21.0;28.0]	*0.014*	233
Haemoglobin, g/dL	13.4 [12.4;14.1]	13.2 [12.3;14.2]	*0.518*	230
Change in Haemoglobin, g/dL	-0.20 [-0.60;0.40]	-0.30 [-0.80;0.20]	*0.114*	228
Ferritin, μg/L	100 [47.0;181]	122 [57.0;194]	*0.210*	233

Abbreviations: ALT, alanine aminotransferase; AST, aspartate aminotransferase;eGFR, estimated glomerular filtration rate; GGT, gamma glutamyl transferase.

© 2023 Ledwidge M et al. *JAMA Cardiology.*

eFigure 2. Change in per-protocol maximal left atrial volume index using cardiac MRI

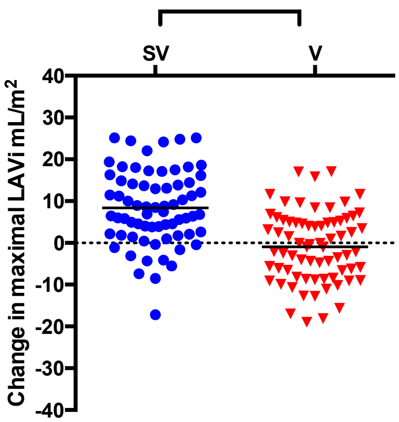

There were delays in obtaining baseline cardiac MRI analyses in 45 people, which further resulted in analyses being carried out in a different magnet and different institution. Furthermore, lockdown restrictions due to Covid 19 resulted in 48 people delaying or cancelling follow up cardiac MRI. In the per-protocol analysis of n=148 patients, the result was the same as with the intention to treat result (p<0.0001). Median cardiac MRI-estimated change in maximal LAVi was greater in patients assigned to receive sacubitril/valsartan (7.4 mL/m^2; 95% CI, 6.2, 10.5) versus valsartan (-1.04 mL/m^2; 95% CI, -2.9, 1.0) *P*<.0001. *P* value indicates between-group comparison of change values over time, adjusted for age, gender, hypertension, diabetes, obesity, vascular disease and maximal LAVi.

Abbreviations: LAVi, left atrial volume index; SV, sacubitril/valsartan; V, valsartan.

© 2023 Ledwidge M et al. *JAMA Cardiology.*

eFigure 3. Time to first serious adverse cardiovascular event

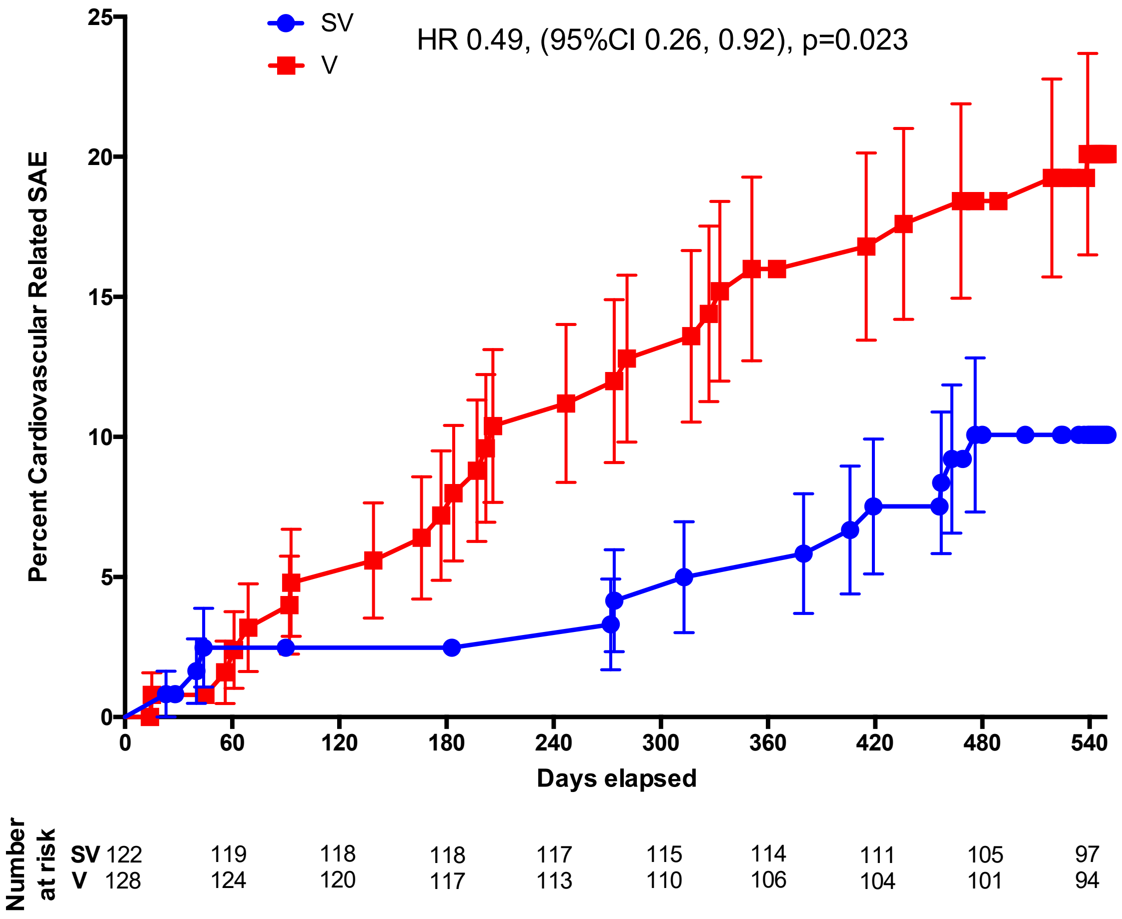

Sacubitril/valsartan (blue circles) was associated with an unadjusted hazard ratio for cardiovascular death and major adverse cardiovascular events of 0.49 (95%CI 0.26 to 0.92, *P*=.023) versus valsartan (red squares).

Abbreviations: CV, cardiovascular; SAE, serious adverse event; SV, sacubitril/valsartan; V, valsartan.

© 2023 Ledwidge M et al. *JAMA Cardiology.*

eTable 8. Serious Adverse Event (SAE) over the study data by System-Organ-Class

**System Organ Class (SOC)** / Preferred term (PT)	**n**	**sacubitril/valsartan (n=122)**	**Valsartan (n=128)**
**Blood and lymphatic disorders**	-	-	-
Anaemia	1	1	
Total	*1*	*1*	*0*
**Cardiovascular disorders**	-	-	-
Acute coronary syndrome	1		1
Acute myocardial infarction	2	1	1
Atrial fibrillation/flutter	15	5	10
Bradycardia	1	1	
Cardiac failure	2	2	
Cardiac failure acute	1		1
Cardiac failure congestive	1		1
Coronary artery disease	2		2
Myocardial infarction	3	2	1
Myocardial ischaemia	1		1
Palpitations	1	1	
Trifascicular block	1		1
Unstable angina	1		1
Cerebrovascular accident	1	1	
Haemorrhagic stroke	1		1
Presyncope	3	2	1
Syncope	2		2
Transient global amnesia	1		1
Transient ischaemic attack	3	1	2
Aortic aneurysm	1	1	
Haematoma	1	1	
Orthostatic hypotension	2		2
Peripheral artery aneurysm	1	1	
Total	*48*	*19*	*29*
**Ear and labyrinth disorders**	-	-	-
Vertigo	1		1

© 2023 Ledwidge M et al. *JAMA Cardiology.*

Total	*1*	0	1
**Eye disorders**	-	-	-
Macular hole	1		1
Total	*1*	0	*1*
**Gastrointestinal disorders**	-	-	-
Abdominal pain upper	1		1
Diarrhoea	1		1
Diverticular disease	1	1	
Total	*3*	*1*	*2*
**General disorders and administration site conditions**	-	-	-
Chest pain	2	1	1
Total	*2*	*1*	*1*
**Hepatobiliary disorders**	-	-	-
Cholelithiasis	1	1	
Cholecystitis	1	1	
Total	*2*	*2*	*0*
**Infections and infestations**	-	-	-
Cellulitis	1		1
Clostridium difficile infection	1	1	
Community acquired pneumonia	1		1
Diverticulitis	3	3	
Herpes zoster	1	1	
Infected dermal cyst	1	1	
Influenza	1	1	
Lower respiratory tract infection	3	1	2
Osteomyelitis	1	1	
Pneumonia	2	1	1
Pyelonephritis	2		2
Sepsis	1		1
Urinary tract infection	1		1
Urosepsis	1	1	
Vestibular neuronitis	1	1	

© 2023 Ledwidge M et al. *JAMA Cardiology.*

Total	*21*	*12*	*9*
**Injury, poisoning and procedural complications**	-	-	-
Ankle fracture	1		1
Foot fracture	1	1	
Pubis fracture	1	1	
Toxicity to various agents	2		2
Urethral injury	1		1
Wound dehiscence	1		1
Total	*7*	*2*	*5*
**Metabolism and nutrition disorder**	-	-	-
Diabetes mellitus	1	1	
Hypoglycaemia	1	1	
Hypokalaemia	1	1	
Hyponatraemia	2	2	
Total	*5*	*5*	*0*
**Musculoskeletal and connective tissue disorders**	-	-	-
Flank pain	1		1
Lumbar spinal stenosis	1		1
Osteoarthritis	3		3
Rheumatoid arthritis	1	1	
Spinal osteoarthritis	1		1
Spinal column stenosis	1	1	
Total	*8*	*2*	*6*
**Neoplasms benign, malignant and unspecified (incl cysts and polyps)**	-	-	-
Carcinoid tumour of the caecum	1	1	
Chronic lymphocytic leukaemia	1		1
Colon cancer	1	1	
Colorectal cancer stage IV	1		1
Lung adenocarcinoma	1	1	

© 2023 Ledwidge M et al. *JAMA Cardiology.*

Malignant urinary tract neoplasm	1		1
Meningioma	1		1
Myeloproliferative neoplasm	1		1
Papillary thyroid carcinoma	1	1	
Prostate cancer	2		2
Total	*11*	*4*	*7*
**Nervous system disorders**	-	-	-
Dementia Alzheimers type	1		1
Muscular weakness	1		1
Total	*2*	*0*	*2*
**Psychiatric disorders**	-	-	-
Confusional state	1	1	
Total	*1*	*1*	
**Renal and urinary disorders**	-	-	-
Acute kidney injury	4		4
Renal impairment	1	1	
Total	*5*	*1*	*4*
**Respiratory, thoracic and mediastinal disorders**	-	-	-
Epistaxis	1	1	
Interstitial lung disease	1	1	
Pulmonary oedema	1	1	
Respiratory failure	1		1
Total	*4*	*3*	*1*
**Skin and subcutaneous tissue disease**	-	-	-
Angioedema	1	1	
Total	*1*	*1*	
**Surgical and medical procedures**			
Spinal decompression	1		1
Total	*1*	0	*1*
**TOTAL no of SAEs (cumulative)**	**124**	**55**	**69**

© 2023 Ledwidge M et al. *JAMA Cardiology.*

© 2023 Ledwidge M et al. *JAMA Cardiology.*
