# Supplementary material for: Effect of Sacubitril/Valsartan vs Valsartan on Left Atrial Volume in Patients With Pre–Heart Failure With Preserved Ejection Fraction: The PARABLE Randomized Clinical Trial
Source: JAMA Cardiol. 2023 Mar 8;8(4):366–75. doi: 10.1001/jamacardio.2023.0065 (PMC9996460; doi:10.1001/jamacardio.2023.0065)
Supplement: Supplement 3. — Data sharing statement [file jamacardiol-e230065-s003.pdf]

Data Sharing Statement

Ledwidge. Association of Sacubitril/Valsartan vs Valsartan With Left Atrial Volume in Patients With Pre-Heart Failure With Preserved Ejection Fraction. *JAMA Cardiol*. Published March 08, 2023. doi:10.1001/jamacardio.2023.0065

Data
Data available: Yes
Data types: Other (please specify)
Additional Information: Data will be made available to research collaborators on request and subject to Data Protection Agreements.
How to access data: On request from the first and last authors; mark.ledwidge@ucd.ie and kenneth.mcdonald@ucd.ie
When available: With publication

Supporting Documents
Document types: Statistical/analytic code, Informed consent form
How to access documents: On request from the first and last authors; mark.ledwidge@ucd.ie and kenneth.mcdonald@ucd.ie
When available: With publication

Additional Information
Who can access the data: Data will be made available to research collaborators on request and subject to Data Protection Agreements.
Types of analyses: Specified purpose, related to novel analyses
Mechanisms of data availability: Following ethics approval and with a signed data access agreement
Any additional restrictions: None
